# The tRNA-Cys-GCA Derived tsRNAs Suppress Tumor Progression of Gliomas via Regulating VAV2

**DOI:** 10.1155/2022/8708312

**Published:** 2022-11-15

**Authors:** Jian Ren, Xiaoling Wu, Fei-Fei Shang, Yingqiang Qi, Zhurong Tang, Chunjie Wen, Weiguo Cao, Quan Cheng, Lihong Tan, Huan Chen, Hong-Hao Zhou, Hecun Zou

**Affiliations:** ^1^Institute of Life Sciences, Chongqing Medical University, Chongqing 400016, China; ^2^Department of Neurology, The Affiliated Hospital of Chongqing Medical and Pharmaceutical College, Chongqing 401331, China; ^3^Chongqing University Cancer Hospital, Chongqing 400030, China; ^4^Xiangya Hospital, Central South University, Changsha, 410008 Hunan, China

## Abstract

The tsRNAs (tRNA-derived small RNAs) are new types of small noncoding RNAs derived from tRNAs. Gliomas are well-known malignant brain tumors. The study focused on tsRNA characterizations within gliomas. Datasets processing, bioinformatics analyses, and visualizations were performed with the packages of Python and R. Cell proliferations were demonstrated via CCK8 assays and colony formation assays, and *in vivo* xenograft experiments. Dual-luciferase reporter assay was performed to confirm the binding of tsRNA with its targets. Via using bioinformatics approaches, the hundreds of tsRNAs with available expression abundance were identified in gliomas dataset, most of them derived from D-loop or T-loop fragments of tRNAs. Among tsRNAs derived from tRNA-Cys-GCA, tRFdb-3003a and tRFdb-3003b (tRFdb-3003a/b) were remarkably down-regulated in gliomas. The survival outcome of gliomas patients with low tRFdb-3003a/b expressions was notably worse than that of high-expression patients. In glioma cells, tRFdb-3003a could suppress cells proliferation and colony formation ability. *In vivo*, tRFdb-3003a suppressed the tumor growth of xenograft gliomas. Enrichment analyses displayed the tRFdb-3003a-related mRNAs were enriched in the specific GO terms, spliceosome and autophagy pathways, and three GSEA molecular signatures. Mechanically, 3'-UTR regions of *VAV2* mRNA were predicted to contain the binding positions of tRFdb-3003a/b, tRFdb-3003a and tRFdb-3003b was effective to reduce the relative luciferase activity of cells with *VAV2* wild-type reporter. Overexpression of tRFdb-3003a/b could down-regulated the expression levels of VAV2 protein and mRNA in glioma cells. The tRNA-Cys-GCA derived tRFdb-3003a and tRFdb-3003b might act as key player in tumor progressions of gliomas; tRFdb-3003a/b might directly bind to VAV2 and regulate VAV2 expressions in gliomas.

## 1. Introduction

The tRNA (transfer RNA) derived small RNAs (tsRNAs) are new types of small noncoding RNAs (sncRNA) that derived from tRNAs, and also known as tRNA-derived fragments (tRFs) [1]. The identifications of tsRNA have been raised sharply during last few years, largely due to the rapid development of next-generation-sequencing approaches to molecular biology and biochemistry [2]. A tsRNA is about 25 nucleotides in length and derives from the fragment of precursor or mature tRNAs at multiple loci [3]. The categories of tsRNA are based on their loci that corresponding to its parental tRNA sequences, and may be divided to several different types, include tRF-5, tRF-3, and i-tRF, these fragments comprise part of whole mature tRNAs; others like 5′U-tRFs and tRF-1 are originated from the immediate transcript of tRNA precursors [3, 4]. A growing number of studies believe that the production of tRNA into specific fragments is a rigorous process, not only as a consequence of degradation. The fragments of tRNA may be context-dependent, including genders, races, diseases, and tissues of individual. The dependencies urgently require some efforts, such as experimental and computational approaches, to understand the regulative functions of tsRNA [2, 5].

Deep analysis of some sequence data have excavated numerous miRNA isoforms [6], likewise, tsRNAs are also the type of small noncoding RNAs with substantial sequencing abundance. Great efforts tried to characterize and generalize the tsRNAs that found from some higher-organisms into a repository, and constructed the tRFdb database [7]. Recent researches have linked tRNA fragments to several human carcinoma diseases, such as colon carcinoma [8], breast, and pancreas cancers [9, 10]. Increasing evidence suggested that vital tsRNAs might function as possible candidates for diagnostic and prognostic biomarker, even contributed to the development and progression of human cancers [11]. Gliomas, especially astrocytoma, are well-known malignant brain tumors [12, 13]. According to epidemiological statistics, mortality due to gliomas is the highest among brain and central nervous system (CNS) cancers [14]. The pathogeneses of glioma are terribly complex that involve the abnormal activations of oncogenes and inactivation of tumor suppressor genes [15, 16], the tsRNAs may be function as new potential diagnostic and prognostic biomarkers for gliomas.

In the present study, we aimed at identifying a tsRNA profile within the sncRNA-sequencing data of glioma tissues through the reliable tsRNA mining pipelines, and then reveal the expression patterns and biological roles of tRNA-Cys-GCA derived tsRNAs (tRFdb-3003a and tRFdb-3003b) in gliomas. In addition, a relationship between tRFdb-3003a/b and its target genes is also investigated in order to display the underlying mechanism.

## 2. Methods

### 2.1. Data Preprocessing and tsRNA Identification

The small noncoding RNA sequence data of gliomas on the Cancer Genome Atlas (TCGA), the RNA sequence datasets of LGG (low grade gliomas), and the corresponding patients information were downloaded from GDC data portal [17]. The primary clinical information and molecular pathology characteristics of glioma patients were listed in Table S1. The genes expression profile of GSE4290 was retrieved from the GEO databases, the genes expression microarray is based on Affymetrix Human Genome U133-Plus-2.0 Array (Affymetrix, USA) [18]. The sequencing data of human tsRNA genes were taken from tRFdb (http://genome.bioch.virginia.edu/trfdb/) and tRFexplorer (https://trfexplorer.cloud/) databases [7, 19]. The GRCh37/hg19 genome annotation and corresponding sequence of tRNA genes in humans were taken from GtRNAdb database (http://gtrnadb.ucsc.edu/), the custom annotations of the GRCh37 reference genome containing most known tsRNAs were assembled [20].

The datasets preprocessing and flow-chart for tsRNA characterizations were displayed in Figure S1A, the reliable pipelines were employed to precisely obtain the estimates of tsRNA expressions from sncRNA-sequencing data as what mentioned before [19, 21]. Briefly, the clean reads of sncRNA-sequencing data were aligned to the reference GRCh37 genome and the sequences of custom tsRNA annotation files via using Bowtie method [22]. After alignments, only the aligned reads can be quantified to count the number of read belonging to each tsRNA candidates with HTSeq method [23], other ambiguous reads were filtered for the more conservative analyses. Finally, the expression values of tsRNA were calculated as reads per million mapped reads (RPM) of raw counts, and these tsRNAs with average expression levels less than one log2(RPM) were removed to discard random degradation fragments [19, 24]. The data analyses were performed with Python and R software.

### 2.2. Data-Frame Analysis and Visualizations

The RNA expression data of TCGA were obtained and processed via TCGAbiolinks packages of Bioconductor [25]. Hierarchical cluster analyses were performed on tsRNA expressions and main pathological characteristics, and the heatmaps were plotted by pheatmap package (https://cran.r-project.org/web/packages/pheatmap/). Survival curve analyses with Kaplan-Meier methods plus log-rank tests were conducted through the survminer and survival packages within R software [26]. Pearson correlations were evaluated for each pair of tsRNA and candidate mRNAs, the scatter diagrams were drawn via using ggpubr and ggplot2 packages (https://ggplot2-book.org). The enrichment analysis was conducted to provide biology views regarding tRFdb-3003a/b-related mRNAs, the MSigDB database (https://www.gsea-msigdb.org/gsea/msigdb/) were applied as potential genes sets for the gene-sets enrichment analysis (GSEA) [27]. Gene ontology (GO), KEGG pathway, and GSEA analyses were conducted by using clusterProfiler and enrichplot packages (https://yulab-smu.github.io/clusterProfiler-book/). The potential binding interactions between tsRNAs and target-genes were predicted through tRFtarget website with RNAhybrid plus IntaRNA tools [28]. An interaction relationship of top fifty targets and tRFdb-3003a/b was organized and visualized as a network-map by applying Cytoscape software.

### 2.3. Gliomas Tissues Specimens

Forty glioma tissues were obtained from patients diagnosed with gliomas undergoing surgical resections at the Department of Neurosurgery of Xiangya Hospital of Central South University from July 2015 to December 2018. After excisions, tissues were immediately frozen in liquid nitrogen for subsequent use. Twelve non-tumor brain tissues were obtained from the adult patients with craniocerebral injuries, which required partial resections of brain tissues as decompression treatment to reduce intracranial pressures. This study was approved by the Ethics Committees of Central South University and the patients provided written informed consents [12].

### 2.4. RNA Extraction and qRT-PCR Analysis

Total RNA was extracted from tissues or cultured cells using the TRIzol reagent (Invitrogen, USA). One microgram total RNAs of each sample was reversely transcribed into cDNA under standard condition via using PrimeScript RT reagent Kit with gDNA Eraser (Takara, Japan). Quantitative real-time polymerase chain reaction (qRT-PCR) was performed with the SYBR® Premix DimerEraser™ (Takara, Japan) on the LightCycler® 480 system (Roche Diagnostics, Switzerland). The Bulge-Loop miRNA qRT-PCR Stater Kit (Ribobio, China) with specific stem-loop RT primers was used to quantify the expression of tsRNAs. ACTB (*β*-actin) was used as the internal control for mRNA template normalizations, and RNU6 (U6) for tsRNAs template normalizations. Relative quantification of gene expression was calculated by the comparative cycle-threshold methods [15]. Stem-loop primers for tRFdb-3003a/b and U6 were designed and synthesized by RiboBio BioTech (Guangzhou, China). The general primers for VAV2 and ACTB were designed and synthesized by Sangon Biotech (Shanghai, China), and the primers sequences were listed in Table S2.

### 2.5. Cyto-Biology Assays

Human glioma cell lines T98G and U251 were purchased from the Cell Bank of Chinese Academy of Sciences (Shanghai, China). The cells were cultured in Dulbecco's Modified Eagle's Medium (DMEM; Gibco) supplemented with 10% fetal bovine serum (FBS; Gibco) at 37°C in a humidified incubator with 5% CO_2_. The cell lines at 55~70% confluences were transfected with micromolar either mimic for tRFdb-3003a/b (tRF3003a-mimic, tRF3003b-mimic), or inhibitor for tRFdb-3003a (tRF3003a-inhibitor), or negative controls (tRF-NC mimic, tRF-NC inhibitor) using the Lipofectamine RNAimax reagent (Invitrogen, USA). The mimic and inhibitor for tRFdb-3003a/b and negative control were purchased from RiboBio (Guangzhou, China). The micrON™ tRF-3003a agomir and negative control were also purchased from RiboBio (Guangzhou, China). Cell proliferation assays were performed with Cell Counting Kit-8 (CCK-8; Dojindo Laboratories, Japan), the absorbance (A) in each well was measured at 450 nm with a Microplate Reader (BioTek, VT, USA). Cell proliferation was also assessed by colony formation assay. Visible colonies were manually counted and photographed.

### 2.6. *In Vivo* Xenograft Experiments

The mice experiments were performed according to the procedure described previously [8]. Briefly, five-week-old male athymic BALB/C mice were maintained under specific pathogen-free (SPF) conditions and manipulated according to protocols approved by the Central South University Experimental Animal Care Commission. All experimental procedures involving animals were complied with the Guide for the Care and Use of Laboratory Animals. U87 cells transfected with tRF-3003a agomir or tRF-NC agomir were harvested and implanted subcutaneously into the flanks of each nude mouse, one injection per mouse. After three weeks of continuous monitoring, twelve xenograft mice were sacrificed, and their tumor tissues were excised and used to perform immunostaining analysis. For staining, five microns sections were cut, dehydrated, deparaffinated, and rehydrated. HE (Hematoxylin-eosin) staining was performed according to the standard protocols. The images were captured using a Leica microscope (Heidelberg, Germany).

### 2.7. Dual-Luciferase Reporter Assay

The wild-type or mutant sequences of VAV2 3'-UTR (3′-untranslated regions) containing putative tRFdb-3003a and tRFdb-3003b binding sites were subcloned into pmir-RB-Report vector (RiboBio, China). Glioma cells were cotransfected with pmir-RB-Report vector plus or without tRFdb-3003a/b mimic. The luciferase activity was measured using the Dual-luciferase Reporter Assay System (Promega, USA).

### 2.8. Protein Extraction and Western Blot

Total protein was extracted from cultured cells using RIPA buffer (Beyotime, China) and the protein concentration was determined using a BCA Protein Assay Kit (Bio-Rad). The specific primary antibody (anti-VAV2 antibody diluted at 1 : 500, Santa Cruz, USA; anti-*β*-actin antibody diluted at 1 : 1000, Sigma-Aldrich, USA). The *β*-actin served as an endogenous protein for normalization. The protein bands were visualized using ChemiDocTM imaging system and Image Lab software (Bio-Rad, California, USA).

### 2.9. Statistical Analysis

The statistical analysis was performed with the R software, GraphPad Prism v8.0 was also used for graphing and analysis. Data were exhibited as means ± standard deviation. The *χ*^2^ test was used to compare the categorical variables. Regarding the numerical variables, statistical significance of differences between two groups was assessed using *Student*'s *T*-test; and comparisons of multiple groups were made by ANOVA analysis. All experiments were performed in triplicates and *P* values less than 0.05 were considered a statistically significant difference.

## 3. Results

### 3.1. The Identification of tsRNAs in Gliomas Data

Firstly, we have gathered more than thousand human tsRNAs sequences from tRFexplorer [19] and tRFdb databases [7], the tsRNAs sequence could re-mapped to specific region of parental tRNAs. The statistical analyses of all tsRNAs have showed that most of tRF-5 and tRF-3 are separately cleaved at the D-loop and T-loop of mature tRNA, others 5′U-tRFs and tRF-1 are derived from the immediate transcript of tRNA precursors. With computational approaches, we have identified nearly three hundred tsRNAs and characterized their expression profiles within the sncRNA-sequence data of TCGA-LGG datasets, a flow chart of the identification procedure is displayed in Figure S1A. After screening, nearly two hundred tsRNAs with available expression abundances were determined within glioma specimens (Table S3 and Table S4), of which about 33% tsRNAs derived from the T-loop fragment (tRF-3) and 26% tsRNAs from the D-loop fragment (tRF-5) of mature tRNA (Figure S1C). As for the classification of chromosome loci, it showed that more than forty percent of tsRNAs could be re-mapped to the human chr-1 locus, and twenty percent re-mapped to the chr-6 and chr-17 loci (Figure S1B).

**Figure 1:**
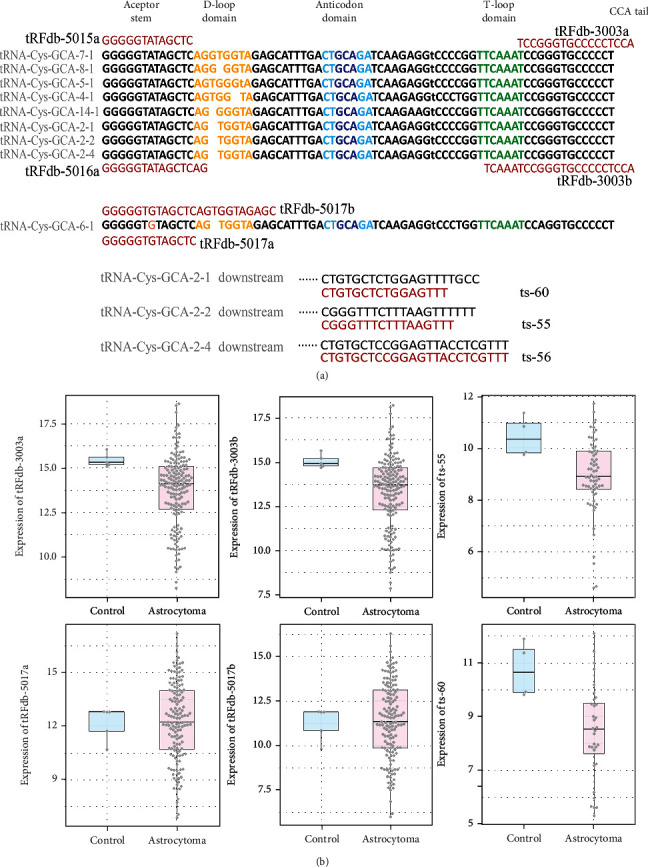
(a) Representative images of the sequence alignments between tsRNA fragments and candidate tRNA-Cys-GCA sources. (b) The expression patterns of several tRNA-Cys-GCA derived tsRNA fragments (tRFdb-3003a, tRFdb-3003b, ts-55, tRFdb-5017a, tRFdb-5017b, and ts-60) in glioma samples (astrocytoma vs nontumor controls) of TCGA-LGG datasets.

**Figure 2:**
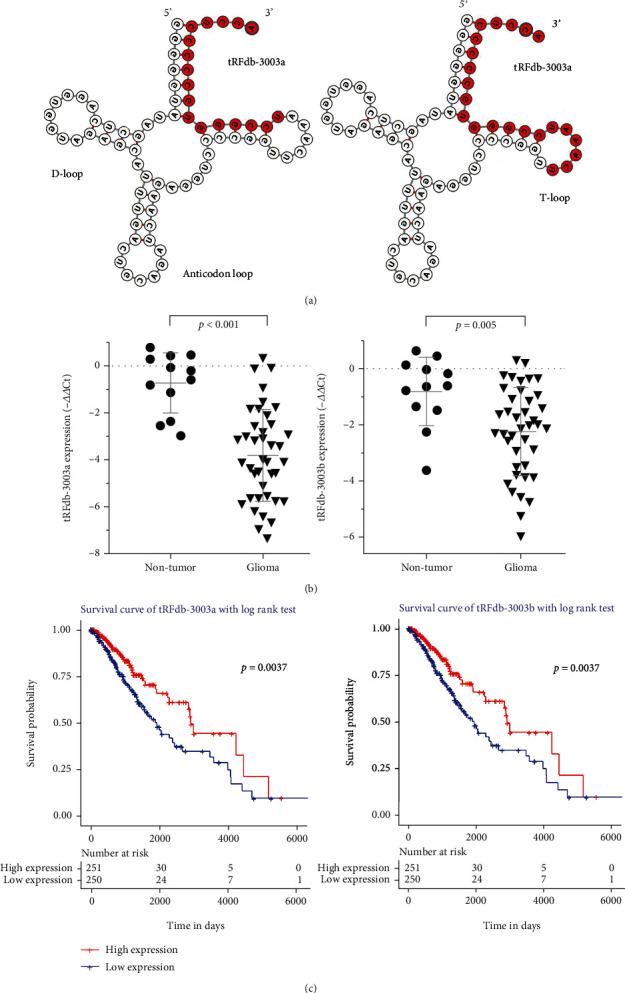
The clinical significance of tRFdb-3003a and tRFdb-3003b in gliomas. (a) Secondary structure of tRNA-Cys-GCA-7-1 and its derived fragments (tRFdb-3003a and tRFdb-3003b, from OncotRF database). (b) The qRT-PCR analysis of relative tRFdb-3003a and tRFdb-3003b expression between glioma tissues and nontumor controls. (c) Kaplan–Meier survival curve analysis with a log-rank test based on tRFdb-3003a and tRFdb-3003b expression within glioma samples (*n* = 568) of TCGA-LGG datasets.

Among them, the tsRNAs derived from tRNA-Cys-GCA (cysteinyl transfer-RNA) caught our attention. As shown in Figure 1(a), nine tsRNAs have been identified that may be derived from tRNA-Cys-GCA genes, there are four tRF-5 fragments (tRFdb-5015a, tRFdb-5016a, tRFdb-5017a, and tRFdb-5017b) can re-map to the D-loop fragments of several distinct tRNA-Cys-GCA isoforms, two tRF-3 (tRFdb-3003a and tRFdb-3003b) can re-map to the T-loop fragments of tRNA-Cys-GCA isoforms (Figure 2(a)) [29], other tRF-1 (ts-55, ts-56 and ts-60) that re-mapping to the downstream region (30 bases) of tRNA-Cys-GCA precursor.

### 3.2. The Significance of tsRNAs Derived from tRNA-Cys-GCA in Gliomas

To understand the role of tsRNAs on gliomas, the expression pattern of selected tsRNAs were analyzed in the specimens of TCGA-LGG dataset. It displayed that the expressions of four tsRNAs (including tRFdb-3003/b, ts-55 and ts-60) were down-regulated in astrocytoma specimens compared to non-tumors (Figure 1(b), all *P* < 0.05), although, no significant difference for other tsRNAs was observed between astrocytomas and non-tumors (both *P* > 0.05). As we known, astrocytomas are the main subtype of gliomas. The clinical tissues detections also displayed the expressions of tRFdb-3003a and tRFdb-3003b was remarkably down-regulated in gliomas compared to non-tumors (Figure 2(b)). These indicated that tRNA-Cys-GCA derived tsRNAs may act as key player in gliomas, notably in astrocytomas.

Subsequently, we have studied the relationships between tsRNA expressions and clinical survival of glioma patients by Kaplan–Meier curve analyses. Base on median values of tsRNA expressions, primary glioma patients were divided into low-expression group and high-expression group. As shown in Figure 2(c), the overall survival of glioma patients with low tRFdb-3003a expression (*P* = 0.0037) was notably worse than that of the high-expression patients; overall survival of patients with low tRFdb-3003b expression was also worse than that of high-expression patients (*P* = 0.0037). These data implied that down-regulated tRFdb-3003a and tRFdb-3003b were associated with poor survival outcome of gliomas.

**Figure 3:**
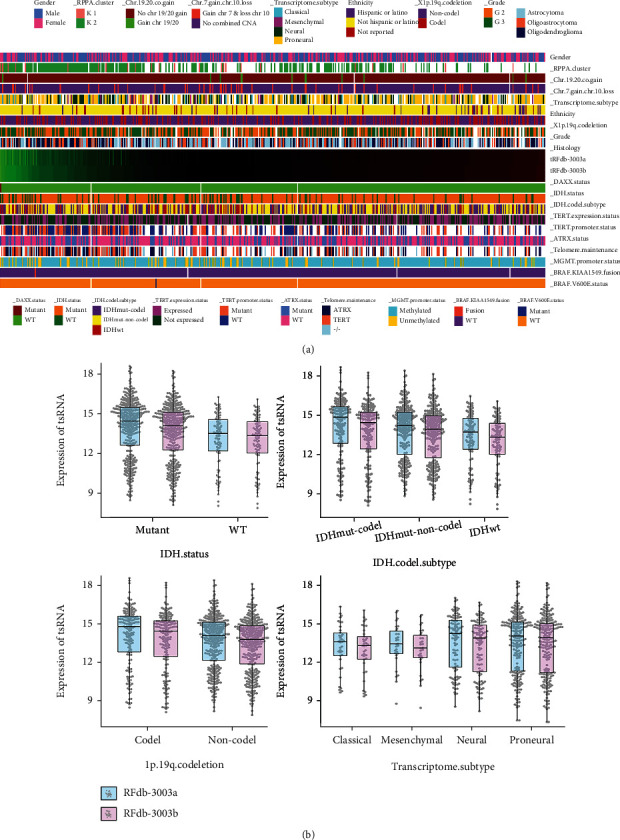
The correlation analysis of tRFdb-3003a/b expression with the primary pathology characteristics. (a) Hierarchical clustering heatmap of tRFdb-3003a/b expression and the pathology characteristics parameters. (b) The bee-swarm plus box plots between tRFdb-3003a/b expression with four pathology parameters (such as *IDH* status, *IDH*-codel subtype, 1p19q codeletion, and transcriptome subtype).

According to the CNS WHO classification, the primary molecular pathological characteristics of gliomas patient were showed in Table S1 [13]. As shown in Figures 3(a) and 3(b), Pearson correlation of tRFdb-3003a/b expression with pathological characteristics was analyzed by *χ*^2^ test, and was visualized via Hierarchical cluster heatmaps. The expressions of tRFdb-3003a/b was significantly correlated with *IDH*-mutants status in glioma patients (both *P* < 0.05), tRFdb-3003a and tRFdb-3003b may tend to raise in the patients with IDH-mutants. Moreover, high tRFdb-3003a/b expression was correlated with 1p19q-codeletion in glioma patients (both *P* < 0.05). As for transcriptome classifications, tRFdb-3003a and tRFdb-3003b may be more expressed in the proneural subtype of gliomas; although, no significant correlation was found between tRFdb-3003a/b expressions and other pathological characteristics such as sex, grades, *MGMT* promoter methylation, BRAF, and TERT status (all *P* > 0.05). As might be expected, heterozygous mutations in the catalytic arginine residues of isocitrate dehydrogenase gene (IDH1/2) are common in gliomas and acute myeloid leukemia, and contribute to the pathogenesis of several tumors [30]. Additionally, the IDH-mutants and 1p19q-codeletion have been explored as the diagnostic and prognostic biomarkers for gliomas [13]. These indicated that tRNA-Cys-GCA derived tRFdb-3003a and tRFdb-3003b may play important role in tumor progressions of gliomas.

### 3.3. The Biological Effect of tRFdb-3003a on Glioma Cell Lines

**Figure 4:**
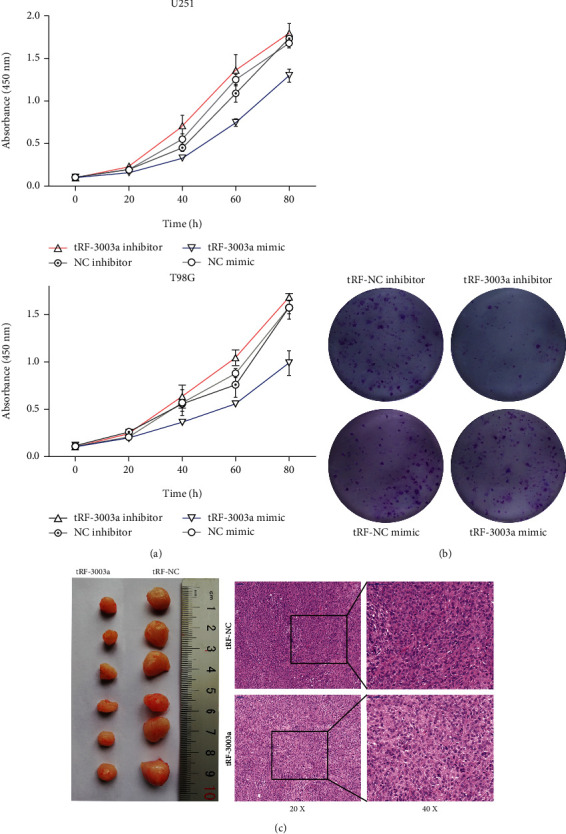
The effects of tRFdb-3003a on cell proliferation and tumor growth of gliomas. (a) CCK-8 assays and (b) colony formation assays were performed to measure the cell proliferation of gliomas *in vitro*. (c) Tumor tissues were excised and photographed within xenograft experiments, the representative images of HE staining. The tRF-3003a mean as tRFdb-3003a, tRF-NC mean as the negative control of tsRNA fragments.

To further explore the biological effect of tRFdb-3003a on gliomas. Specific tRFdb3003a-mimic was used to upregulated tRFdb-3003a expression, and tRFdb3003a-inhibitor was used to inhibit the tRFdb-3003a within glioma cell. The proliferations of glioma cells were detected through CCK8 and colony formation assays. It displayed that the proliferations were evidently suppressed within tRFdb3003a-mimic transfected cells compared to negative controls (both *P* < 0.05; Figure 4(a)). Consistently, the number of colonies was significantly decreased after tRFdb-3003a overexpression in glioma cells (Figure 4(b)). *In vivo* xenograft experiments, the volumes and weights of xenograft tumors in tRF3003a-angmir group were markedly smaller than those in control group (Figure 4(c)-left), the pathological staining revealed some apoptotic morphological changes and showed that tumor growth was decreased in the tRF3003a-angmir group compared to control group (Figure 4(c)-right). These findings implied that tRFdb-3003a might be efficient to regulate glioma cells proliferations *in vitro* and tumor growth of gliomas *in vivo*.

**Figure 5:**
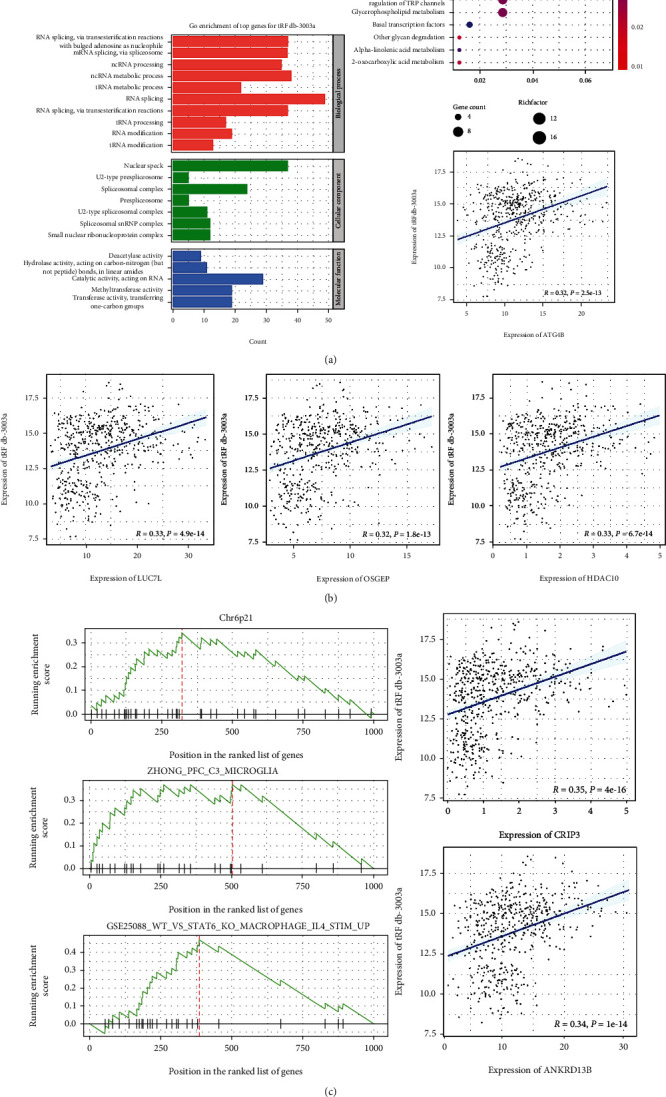
The enrichment analyses of tRFdb-3003a -related genes within TCGA-LGG datasets. (a) The top gene ontology (GO) terms, including biological process, cellular component and molecular function, as well as the top KEGG pathway for tRFdb-3003a-related genes. (b) The correlation scatter-plots of tRFdb-3003a and its correlated-genes (LUC7L, OSGEP, HDAC10, and ATG4B). (c) GSEA (gene set enrichment analysis) plots of three molecular signatures (chr6p21, microglia, and macrophage IL4 immune signature), the scatter plots for tRFdb-3003a and its correlated-genes (CRIP3, ANKRD13B).

Additionally, the enrichment analysis was performed to provide the biology point of view regarding tRFdb-3003a/b. TCGA-LGG mRNA expression dataset was used to find the correlated-mRNAs of tRFdb-3003a and tRFdb-3003b by using Spearman-correlations. As shown in Figure 5 and Figure S2, within the GO analyses, most tRFdb-3003a-related mRNAs were enriched in some GO terms including RNA splicing and metabolic process (OSGEP, LUC7L), enzymatic activity (HDAC10), and nuclear speck (OSGEP). The dot-plot displayed that tRFdb-3003a-related mRNAs were enriched in several KEGG pathways including autophagy and spliceosome pathway (ATG4B). For gene-set enrichment analysis, three molecular signatures (chr6p21, microglia, and macrophage immune signature) were found to associate with tRFdb-3003a expression, their signature genes, CRIP3 and ANKRD13B, were both the tRFdb-3003a correlated-mRNAs. The enrichment analysis of tRFdb-3003b was also performed and showed in supplementary materials (Figure S2).

### 3.4. The tRFdb-3003a/b May Bind and Regulate VAV2 Expressions within Gliomas

**Figure 6:**
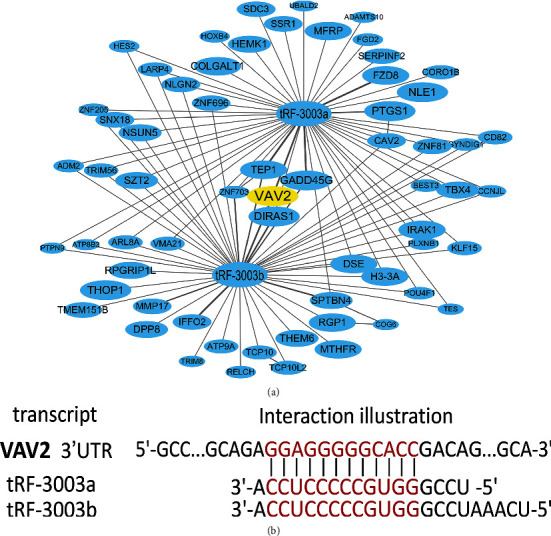
(a) The interaction networks that involved tRFdb-3003a/b and their target genes based on tRFtarget database, the nodes represent target genes, and lines represent the interaction relationships. (b) The putative binding sites of tRFdb-3003a/b within the 3'-UTR regions of *VAV2* based on bioinformatics prediction.

**Figure 7:**
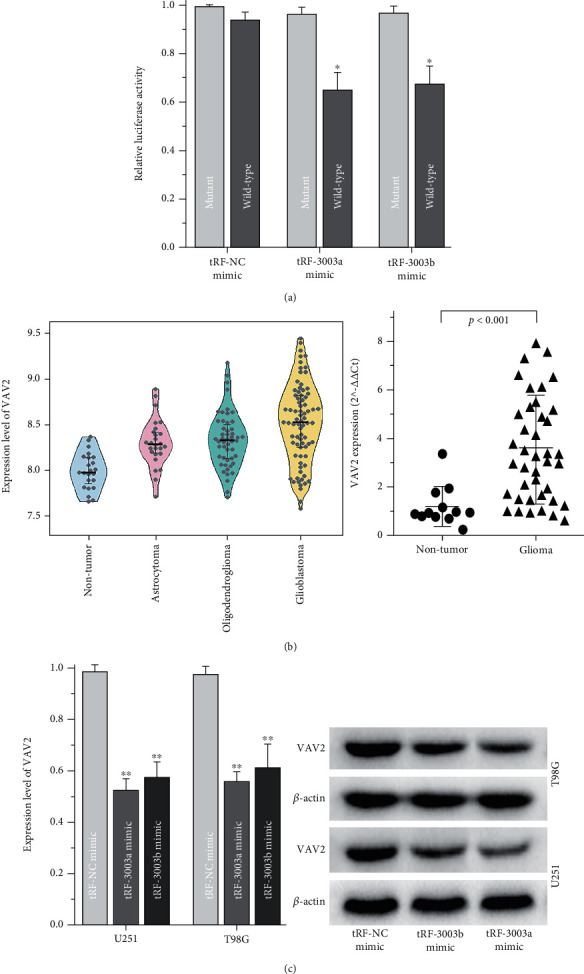
VAV2 is regulated by tRFdb-3003a and tRFdb-3003b in gliomas. (a) The luciferase reporter assay of glioma cells cotransfected with pmir-RB-VAV2 wild-type or mutant report vector, together with tRFdb-3003a/b mimics or controls. (b) The expression pattens of VAV2 within gliomas of GSE4290 datasets and clinical tissue specimens. (c) Expression levels of VAV2 mRNA (left) and protein (right) in glioma cells transfected with tRFdb-3003a/b mimics or controls. The tRF-3003a/b mean as tRFdb-3003a/b, tRF-NC mean as negative control of tsRNA fragments. ^∗∗^*P* < 0.01, ^∗^*P* < 0.05.

Recent studies have reported that tsRNA could bind to Argonautes proteins and silence gene expression by targeting the 3′-untranslated regions (3'-UTRs) of mRNA genes in a manner similar to microRNAs. With bioinformatics methods, the potential target-mRNAs of tRFdb-3003a/b were predicted and picked out. The interactional relationship of tRFdb-3003a/b and the target-mRNAs were showed in Figure 6(a). Among target-mRNAs, we selected a molecular with good binding-score, *VAV2*, which is a protooncogene of the VAV family [31]. The positions in 3′-UTR regions of *VAV2*-mRNA contain potential binding-sites of tRFdb-3003a/b (Figure 6(b)). Luciferase reporter assays were performed to confirm the binding affinity, it demonstrated that relative luciferase activities were reduced in the cotransfection of pmirRB-VAV2 wildtype-reporters with tRFdb3003a/b-mimic (both *P* < 0.05, Figure 7(a)), however no significance in that mutant-reporters. In addition, the analyses of GSE4290 dataset and clinical specimens demonstrated that VAV2 was upregulated in gliomas compared to non-tumors (Figure 7(b)), as noted above, tRFdb-3003a and tRFdb-3003b were down-regulated in gliomas (Figure 1(b)). Furthermore, *in vitro* experiments showed that expression levels of VAV2 protein and mRNA were decreased in glioma cells transfected with tRFdb3003a/b-mimic (Figure 7(c)). These data indicated that tRFdb-3003a/b could directly bind to the 3'-UTR regions of VAV2 and regulate VAV2 expressions within gliomas.

## 4. Discussion

The tRNA-derived small RNAs (tsRNAs) are new types of small noncoding-RNA derived from tRNAs, also known as tRNA-derived fragments [1]. In current researches, we firstly identified the tsRNAs expression profiles in the sncRNA-sequence data of glioma tissues, most of tsRNAs were derived from the T-loop fragment (tRF-3) and the D-loop fragment (tRF-5) of mature tRNAs. Among these tsRNAs, we selected out nine tsRNAs derived from tRNA-Cys-GCA, which is a cysteinyl-transfer-RNA. There are four tRF-5 can re-map to the D-loop fragment of mature tRNA-Cys-GCA, and two tRF-3 (tRFdb-3003a, tRFdb-3003b) can re-map to the T-loop fragment of tRNA-Cys-GCA, other tRF-1 fragments (ts-55, ts-56 and ts-60) that re-mapping to the downstream region of tRNA-Cys-GCA precursors. In which, the expressions of several tsRNAs (such as ts-55, ts-60, tRFdb-3003a, and tRFdb-3003b) were down-regulated in glioma samples compared to non-tumors. Kaplan–Meier analysis showed that the survival of glioma patients with low tRFdb-3003a/b expression was notably worse than that of the high-expression patients; it indicated that down-regulated tRFdb-3003a and tRFdb-3003b were associated with poor survival outcome of gliomas. Further analysis found that tRFdb-3003a/b expressions were significantly correlated with IDH-mutants status and 1p19q-codeletion in glioma samples, tRFdb-3003a and tRFdb-3003b might tend to increase in gliomas with IDH-mutants. These results implied that tRNA-Cys-GCA derived tsRNAs (tRFdb-3003a and tRFdb-3003b) might play important role in tumor progression of gliomas.

Recent researches have linked tsRNAs to several human carcinoma diseases, for instance, colon carcinoma [8], breast and pancreas cancer [9, 10], *etc*. Pan's study has identified an inflammatory cytokine-regulated tsRNA tRF-21 was significantly lower in pancreatic ductal adenocarcinoma (PDAC), and patients with low tRF-21 expressions had a poor prognosis [9]. In PDAC cells, tRF-21 could promote PDAC cell malignant phenotypes via AKT2/1-mediated hnRNP L-DDX17 interaction activity. Moreover, treatment of mouse PDAC xenografts with tRF-21 mimics repressed tumor growth and metastasis, these results demonstrate that tRF-21 has a tumor-suppressive effect for PDAC [9]. In breast cancers, Mo et al. have determined the expression profile of tsRNAs between tumors and controls via sequencing analyses, and found a fragment of tRNA-Val-CAC, 5′-tiRNA^Val^, which is down-regulated in breast cancer tissues [10]. Further studies have demonstrated that 5′-tiRNA^Val^ could suppressed cell viability and colony formations, reduced the migration and invasion of cancer cells, these suggest that 5′-tiRNA^Val^ may serve as a potential antioncogene effect in the development of breast cancers [10]. In the present studies, cyto-biology experiments were performed to explore the biological functions of tRFdb-3003a within glioma cells. It showed that tRFdb-3003a overexpressions suppressed cells proliferations and colony formation of gliomas *in vitro*; and suppressed tumor growth of xenograft gliomas *in vivo*. Additionally, the enrichment analyses displayed tRFdb-3003a was associated with some gene ontology terms, including RNA splicing and metabolic process, enzymatic activity, and nuclear speck; tRFdb-3003a-correlated mRNAs might refer to autophagy and spliceosome pathway, along with three GSEA molecular signatures (chr6p21, microglia, and macrophage immune signature). These data implied that down-regulated tRFdb-3003a could act as anti-oncogene role in glioma progression via specific signal pathways.

Recent studies have suggested that tsRNAs might bind to Argonaute-1/2 protein and act a key player in genes silencing via targeting the 3′-UTR regions of specific mRNA in the manner similar to microRNAs [2], although the biology roles of tsRNA are complicated and require investigation thoroughly. For instance, a tsRNA derived from tRNA-His-GTG, 5′-tiRNA-His-GTG has been found to directly binding the 3'-untranslated regions of *LATS2* gene, which encoded the component of hippo signaling pathway, and suppressed cells proliferations and promoted cells apoptosis through regulating YAP protein activity in colorectal cancers [8]. Bioinformatics analysis showed the interaction relationships of tRFdb-3003a/b and their predicted target-mRNAs, and found tRFdb-3003a/b may target to the 3′-UTR regions of *VAV2*, which was overexpressed in gliomas. Dual-luciferase reporter assays demonstrated that tRFdb-3003a/b could directly bind to the 3′-UTR regions of VAV2 and reduce the relative luciferase activities. Moreover, overexpression of tRFdb-3003a/b could decrease the expressions of VAV2 protein and mRNA within glioma cells. Numerous studies discriminated that *VAV2* is a protooncogene of the VAV family, and has been found critical to Rho GTPase activity in tumor development and maintenance [31]. These findings demonstrated tRFdb-3003a and tRFdb-3003b might directly bind to VAV2 and regulate VAV2 expression in gliomas.

In conclusion, our studies identified many tsRNAs, especially nine tsRNAs derived from tRNA-Cys-GCA, in the sncRNA-sequence data of glioma tissues. Among them, several tsRNAs (ts-55, ts-60, and tRFdb-3003a/b) were remarkably down-regulated in gliomas, the expressions of tRFdb-3003a and tRFdb-3003b were associated with survival outcome and IDH-mutants status of glioma patients. Overexpression of tRFdb-3003a suppressed cells proliferation and tumor growth of gliomas. Down-regulated tRFdb-3003a might function as anti-oncogene role within glioma progression through specific signaling pathways. Moreover, tRFdb-3003a/b could directly bind to the 3′-UTR regions of VAV2 and regulate VAV2 expression in gliomas. These indicated that tRNA-Cys-GCA derived tsRNAs (tRFdb-3003a and tRFdb-3003b) might regulate tumor- progression of gliomas.

## Data Availability

All data and materials used or analyzed during the current study are available from the corresponding author upon reasonable request.

## Supplementary Materials

Supplementary Materials Figure S1: a summary of tsRNAs identification and characterization. (A) the flow chart of data processing and tsRNAs identification pipeline. (C) The characterization of tsRNA corresponding derived tRNA gene sources and (B) the chromosome locations statistical analysis. Figure S2: the enrichment analyses of tRFdb-3003b -related genes within TCGA-LGG datasets. (A) The top gene ontology (GO) terms, including biological process, CC (cellular component) and MF (molecular function), as well as the top KEGG pathway for tRFdb-3003b-related genes. (B) The correlation scatter-plots of tRFdb-3003b and its correlated-genes (ATG4B, LUC7L, D2HGDH, and HDAC10). (C-left) GSEA (gene set enrichment analysis) plots of three molecular signatures (chr6p21, microglia, and STIM treatment response signature), (C-right) the scatter plots for tRFdb-3003b and its correlated-genes (CRIP3 and ANKRD13B). Table S1: the primary clinical and molecular pathology characteristics parameters in glioma samples. Table S2: the primers used in quantitative real-time PCR assay. Table S3: a summary of the identified tsRNAs with available expression abundance in glioma datasets. Table S4: the expression profiles of tRNA-Cys-GCA derived tsRNAs with in glioma samples.
