# Supplementary material for: The tRNA-Cys-GCA Derived tsRNAs Suppress Tumor Progression of Gliomas via Regulating VAV2
Source: Dis Markers. 2022 Nov 15;2022:8708312. doi: 10.1155/2022/8708312 (PMC9681550; doi:10.1155/2022/8708312)
Supplement: Supplementary Materials — Figure S1: a summary of tsRNAs identification and characterization. (A) the flow chart of data processing and tsRNAs identification pipeline. (C) The characterization of tsRNA corresponding derived tRNA gene sources and (B) the chromosome locations statistical analysis. Figure S2: the enrichment analyses of tRFdb-3003b -related genes within TCGA-LGG datasets. (A) The top gene ontology (GO) terms, including biological process, CC (cellular component) and MF (molecular function), as well as the top KEGG pathway for tRFdb-3003b-related genes. (B) The correlation scatter-plots of tRFdb-3003b and its correlated-genes (ATG4B, LUC7L, D2HGDH, and HDAC10). (C-left) GSEA (gene set enrichment analysis) plots of three molecular signatures (chr6p21, microglia, and STIM treatment response signature), (C-right) the scatter plots for tRFdb-3003b and its correlated-genes (CRIP3 and ANKRD13B). Table S1: the primary clinical and molecular pathology characteristics parameters in glioma samples. Table S2: the primers used in quantitative real-time PCR assay. Table S3: a summary of the identified tsRNAs with available expression abundance in glioma datasets. Table S4: the expression profiles of tRNA-Cys-GCA derived tsRNAs with in glioma samples. [file 8708312.f1.zip › Supplemental Material (1).pdf]

Supplementary Materials

Figure titles and legends in the supplemental information files

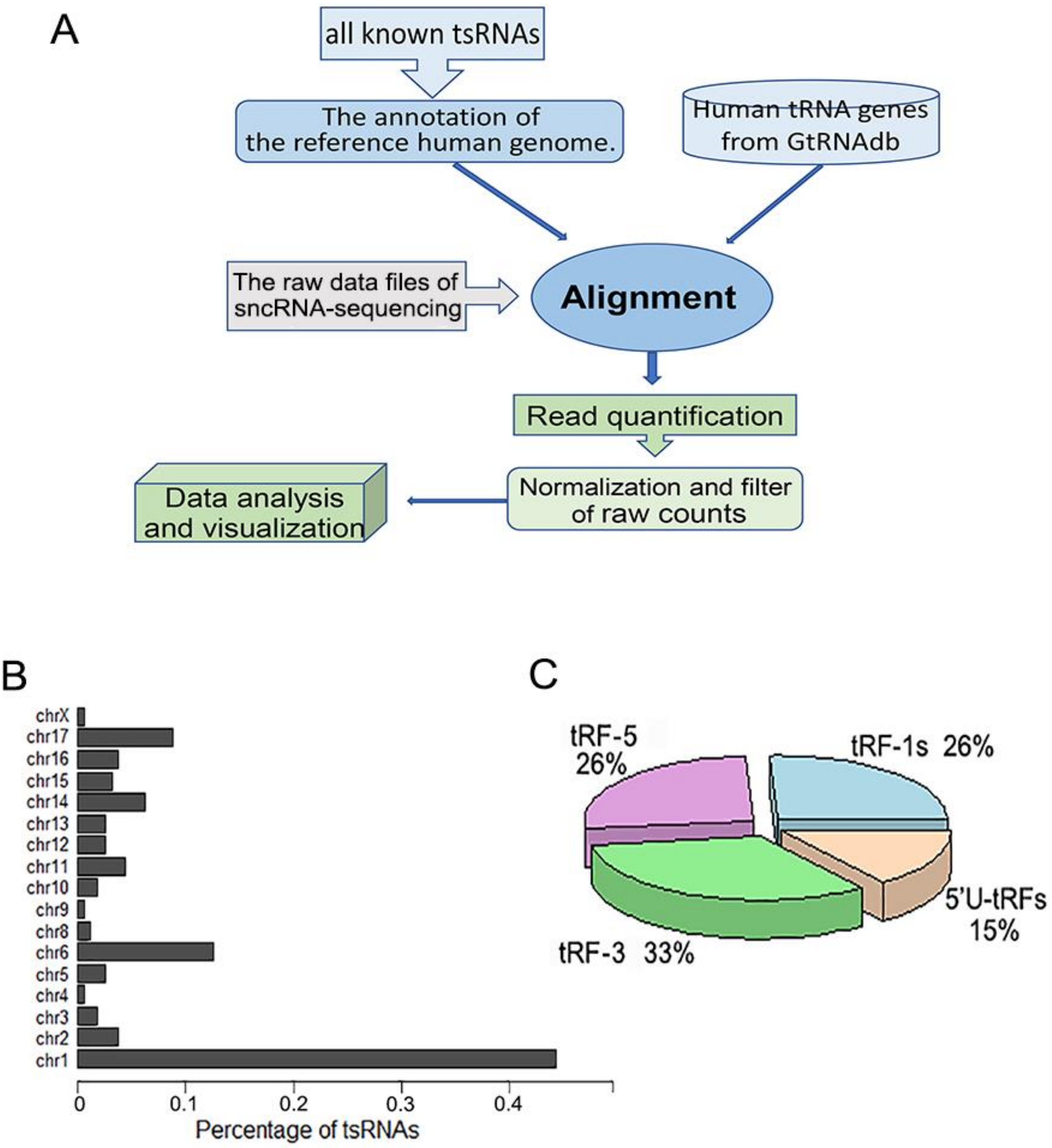

Figure S1. A summary of tsRNAs identification and characterization. (**A**) the flow chart of data processing and tsRNAs identification pipeline. (C) The characterization of tsRNA corresponding derived tRNA gene sources and (**B**) the chromosome locations statistical analysis.

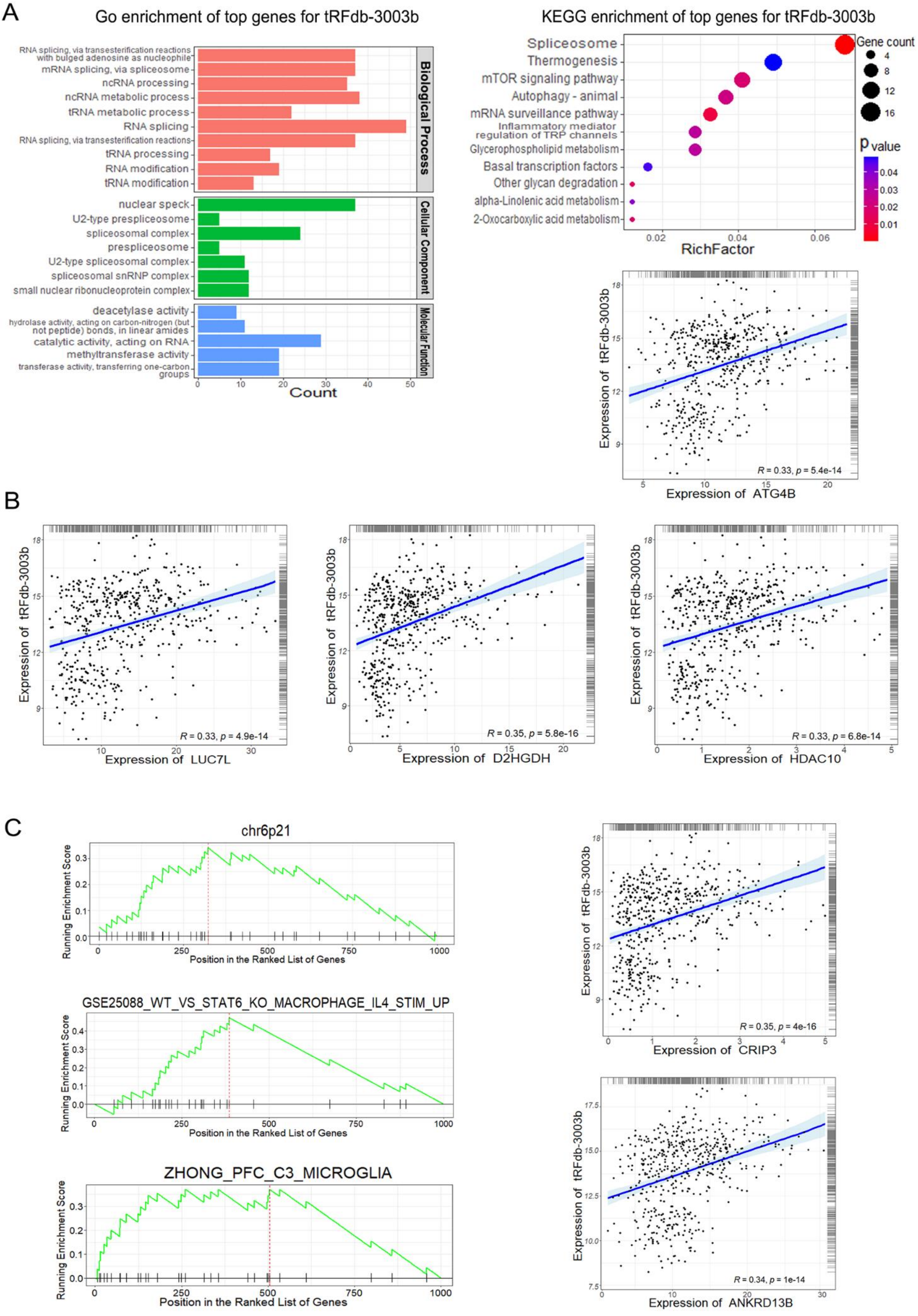
A
Go enrichment of top genes for tRFdb-3003b
RNA splicing, via transesterification reactions with bulged adenosine as nucleophile
mRNA splicing, via spliceosome
ncRNA processing
ncRNA metabolic process
tRNA metabolic process
RNA splicing
RNA splicing, via transesterification reactions
tRNA processing
RNA modification
tRNA modification
Biological Process
nuclear speck
U2-type prespliceosome
spliceosomal complex
prespliceosome
U2-type spliceosomal complex
spliceosomal snRNP complex
small nuclear ribonucleoprotein complex
Cellular Component
deacetylase activity
hydrolase activity, acting on carbon-nitrogen (but not peptide) bonds, in linear amides
catalytic activity, acting on RNA
methyltransferase activity
transferase activity, transferring one-carbon groups
Molecular Function
Count
KEGG enrichment of top genes for tRFdb-3003b
Spliceosome
Thermogenesis
mTOR signaling pathway
Autophagy - animal
mRNA surveillance pathway
Inflammatory mediator regulation of TRP channels
Glycerophospholipid metabolism
Basal transcription factors
Other glycan degradation
alpha-Linolenic acid metabolism
2-Oxocarboxylic acid metabolism
RichFactor
Gene count
p value
Expression of tRFdb-3003b
Expression of ATG4B
R = 0.33, p = 5.4e-14
B
Expression of tRFdb-3003b
Expression of LUC7L
R = 0.33, p = 4.9e-14
Expression of D2HGDH
R = 0.35, p = 5.8e-16
Expression of HDAC10
R = 0.33, p = 6.8e-14
C
chr6p21
Running Enrichment Score
Position in the Ranked List of Genes
GSE25088_WT_VS_STAT6_KO_MACROPHAGE_IL4_STIM_UP
ZHONG_PFC_C3_MICROGLIA
Expression of tRFdb-3003b
Expression of CRIP3
R = 0.35, p = 4e-16
Expression of ANKRD13B
R = 0.34, p = 1e-14

Figure S2. The enrichment analyses of tRFdb-3003b -related genes within TCGA-LGG datasets. (**A**) The top gene ontology (GO) terms, including biological process, CC (cellular component) and MF (molecular function), as well as the top KEGG pathway for tRFdb-3003b-related genes. (**B**) The correlation scatter-plots of tRFdb-3003b and its correlated-genes (ATG4B, LUC7L, D2HGDH, and HDAC10). (**C-left**) GSEA (gene set enrichment analysis) plots of three molecular signatures (chr6p21, microglia, and STIM treatment response signature), (**C-right**) the scatter plots for tRFdb-3003b and its correlated-genes (CRIP3 and ANKRD13B).

Table titles and footnotes in the supplemental information files

Table S1. The primary clinical and molecular pathology characteristics parameters in glioma samples.

Table S2. The primers used in quantitative real-time PCR assay.

Primer names	Primer sequences (5′-3′)
VAV2 forward	CCTCAAGGACATCAACTTCCG
VAV2 reverse	ACCGCGGAGATGACCTTTCCA
ACTB forward	CATGTACGTTGCTATCCAGGC
ACTB reverse	CTCCTTAATGTCACGCACGAT
U6 forward	GGAACGATACAGAGAAGATTAGC
U6 reverse	TGGAACGCTTCACGAATTTGCG

Table S3. A summary of the identified tsRNAs with available expression abundance in glioma datasets.

Table S4. The expression profiles of tRNA-Cys-GCA derived tsRNAs with in glioma samples.

All data and materials used or analyzed during the current study are available from the corresponding author upon reasonable request. The supplemental materials have been deposited at: https://www.aliyundrive.com/s/ziwCstxgNKy.
