# Supplementary material for: The tRNA-Cys-GCA Derived tsRNAs Suppress Tumor Progression of Gliomas via Regulating VAV2
Source: Dis Markers. 2022 Nov 15;2022:8708312. doi: 10.1155/2022/8708312 (PMC9681550; doi:10.1155/2022/8708312)
Supplement: Supplementary Materials — Figure S1: a summary of tsRNAs identification and characterization. (A) the flow chart of data processing and tsRNAs identification pipeline. (C) The characterization of tsRNA corresponding derived tRNA gene sources and (B) the chromosome locations statistical analysis. Figure S2: the enrichment analyses of tRFdb-3003b -related genes within TCGA-LGG datasets. (A) The top gene ontology (GO) terms, including biological process, CC (cellular component) and MF (molecular function), as well as the top KEGG pathway for tRFdb-3003b-related genes. (B) The correlation scatter-plots of tRFdb-3003b and its correlated-genes (ATG4B, LUC7L, D2HGDH, and HDAC10). (C-left) GSEA (gene set enrichment analysis) plots of three molecular signatures (chr6p21, microglia, and STIM treatment response signature), (C-right) the scatter plots for tRFdb-3003b and its correlated-genes (CRIP3 and ANKRD13B). Table S1: the primary clinical and molecular pathology characteristics parameters in glioma samples. Table S2: the primers used in quantitative real-time PCR assay. Table S3: a summary of the identified tsRNAs with available expression abundance in glioma datasets. Table S4: the expression profiles of tRNA-Cys-GCA derived tsRNAs with in glioma samples. [file 8708312.f1.zip › Supplemental Table S1.pdf]

Table S1. The primary clinical and molecular pathology characteristics parameters in glioma samples.

barcode	sample	paper_Histology	_X1p.19q.codeletion	Transcriptome.Subtype	gender	_Telomere.Maintenance	_MGMT.promoter.status	paper_IDH.codel.subtype	paper_IDH.status
TCGA-DU-7309-01A-11R-2090-07	TCGA-DU-7309-01A	oligodendroglioma	non-codel	NE	female	ATRX	Methylated	IDHmut-non-cod	Mutant
TCGA-QH-A6X5-01A-12R-A32Q-07	TCGA-QH-A6X5-01A	oligoastrocytoma	codel	PN	female	NA	Methylated	IDHmut-codel	Mutant
TCGA-CS-6186-01A-12R-2027-07	TCGA-CS-6186-01A	oligoastrocytoma	non-codel	CL	male	TERT	Unmethylate	IDHwt	WT
TCGA-DU-7294-01A-11R-2027-07	TCGA-DU-7294-01A	oligodendroglioma	codel	PN	female	TERT	Methylated	IDHmut-codel	Mutant
TCGA-FG-A70Z-01A-12R-A33Z-07	TCGA-FG-A70Z-01A	oligoastrocytoma	non-codel	CL	female	NA	Methylated	IDHwt	WT
TCGA-E1-A7YS-01A-11R-A34F-07	TCGA-E1-A7YS-01A	NA	codel	PN	male	NA	Methylated	IDHmut-codel	Mutant
TCGA-DU-5871-01A-12R-1708-07	TCGA-DU-5871-01A	oligoastrocytoma	non-codel	PN	female	ATRX	Methylated	IDHmut-non-cod	Mutant
TCGA-DB-A75L-01A-11R-A32Q-07	TCGA-DB-A75L-01A	astrocytoma	non-codel	NE	female	NA	Methylated	IDHmut-non-cod	Mutant
TCGA-R8-A6ML-01A-11R-A32Q-07	TCGA-R8-A6ML-01A	oligodendroglioma	codel	PN	male	NA	Methylated	IDHmut-codel	Mutant
TCGA-FG-5962-01B-11R-1896-07	TCGA-FG-5962-01B	oligodendroglioma	codel	NE	male	TERT	Methylated	IDHmut-codel	Mutant
TCGA-DU-7304-02A-12R-A36H-07	TCGA-DU-7304-02A	NA	NA	NA	male	NA	NA	NA	NA
TCGA-DU-6407-01A-13R-1708-07	TCGA-DU-6407-01A	oligodendroglioma	non-codel	PN	female	ATRX	Methylated	IDHmut-non-cod	Mutant
TCGA-DU-A5TT-01A-11R-A28M-07	TCGA-DU-A5TT-01A	oligodendroglioma	non-codel	NA	male	TERT	Methylated	IDHwt	WT
TCGA-HT-A61A-01A-11R-A29R-07	TCGA-HT-A61A-01A	oligodendroglioma	non-codel	NA	female	TERT	Methylated	IDHmut-non-cod	Mutant
TCGA-P5-A5EW-01A-11R-A27Q-07	TCGA-P5-A5EW-01A	astrocytoma	non-codel	PN	female	ATRX	Methylated	IDHmut-non-cod	Mutant
TCGA-DU-5870-02A-12R-A36H-07	TCGA-DU-5870-02A	NA	NA	NA	female	NA	NA	NA	NA
TCGA-CS-4943-01A-01R-1470-07	TCGA-CS-4943-01A	astrocytoma	non-codel	PN	male	ATRX	Methylated	IDHmut-non-cod	Mutant
TCGA-HT-7607-01A-11R-2090-07	TCGA-HT-7607-01A	astrocytoma	codel	NE	female	TERT	Methylated	IDHmut-codel	Mutant
TCGA-VV-A86M-01A-11R-A36H-07	TCGA-VV-A86M-01A	astrocytoma	non-codel	NA	female	NA	Methylated	IDHmut-non-cod	Mutant
TCGA-S9-A7QW-01A-11R-A34F-07	TCGA-S9-A7QW-01A	astrocytoma	non-codel	PN	female	NA	Methylated	IDHmut-non-cod	Mutant
TCGA-HW-A5KM-01A-11R-A27Q-07	TCGA-HW-A5KM-01A	astrocytoma	non-codel	ME	male	-/-	Methylated	IDHmut-non-cod	Mutant
TCGA-DH-A7UR-01A-11R-A33Z-07	TCGA-DH-A7UR-01A	oligodendroglioma	codel	NA	female	NA	Methylated	IDHmut-codel	Mutant
TCGA-DB-A64V-01A-11R-A29R-07	TCGA-DB-A64V-01A	oligodendroglioma	codel	NA	male	TERT	Methylated	IDHmut-codel	Mutant
TCGA-QH-A65R-01A-21R-A31N-07	TCGA-QH-A65R-01A	oligodendroglioma	codel	PN	female	NA	Methylated	IDHmut-codel	Mutant
TCGA-F6-A8O4-01A-11R-A36H-07	TCGA-F6-A8O4-01A	astrocytoma	non-codel	NA	male	NA	Methylated	IDHmut-non-cod	Mutant
TCGA-CS-6188-01A-11R-1896-07	TCGA-CS-6188-01A	astrocytoma	non-codel	CL	male	TERT	Unmethylate	IDHwt	WT
TCGA-TQ-A7RO-01A-11R-A33Z-07	TCGA-TQ-A7RO-01A	NA	codel	PN	male	NA	Methylated	IDHmut-codel	Mutant

TCGA-TM-A84I-01A-11R-A36H-07	TCGA-TM-A84I-01A	astrocytoma	non-codel	NA	male	NA	Methylated	IDHmut-non-cod	Mutant
TCGA-QH-A6X4-01A-51R-A32Q-07	TCGA-QH-A6X4-01A	oligoastrocytoma	codel	PN	male	NA	Methylated	IDHmut-codel	Mutant
TCGA-DU-7301-01A-11R-2090-07	TCGA-DU-7301-01A	oligodendroglioma	non-codel	NA	male	ATRX	Methylated	IDHmut-non-cod	Mutant
TCGA-FG-A4MT-01A-11R-A26U-07	TCGA-FG-A4MT-01A	oligodendroglioma	non-codel	PN	female	ATRX	Methylated	IDHmut-non-cod	Mutant
TCGA-FG-A4MW-01A-11R-A26U-07	TCGA-FG-A4MW-01A	oligoastrocytoma	non-codel	CL	male	TERT	Methylated	IDHwt	WT
TCGA-HW-7490-01A-11R-2027-07	TCGA-HW-7490-01A	astrocytoma	non-codel	NA	male	ATRX	Methylated	IDHmut-non-cod	Mutant
TCGA-S9-A6WL-01A-21R-A33Z-07	TCGA-S9-A6WL-01A	astrocytoma	codel	NE	male	NA	Methylated	IDHmut-codel	Mutant
TCGA-HT-7689-01A-11R-2256-07	TCGA-HT-7689-01A	oligodendroglioma	non-codel	PN	female	ATRX	Methylated	IDHmut-non-cod	Mutant
TCGA-DB-5274-01A-01R-1470-07	TCGA-DB-5274-01A	oligoastrocytoma	codel	PN	female	TERT	Methylated	IDHmut-codel	Mutant
TCGA-RY-A843-01A-11R-A36H-07	TCGA-RY-A843-01A	NA	non-codel	NA	male	NA	Methylated	IDHmut-non-cod	Mutant
TCGA-S9-A7IX-01A-12R-A34F-07	TCGA-S9-A7IX-01A	astrocytoma	non-codel	CL	male	NA	Unmethylate	IDHwt	WT
TCGA-HT-7882-01A-11R-2403-07	TCGA-HT-7882-01A	oligodendroglioma	non-codel	ME	male	TERT	Methylated	IDHwt	WT
TCGA-QH-A6CZ-01A-11R-A32Q-07	TCGA-QH-A6CZ-01A	oligoastrocytoma	codel	PN	male	NA	Methylated	IDHmut-codel	Mutant
TCGA-FG-A87N-01A-11R-A36H-07	TCGA-FG-A87N-01A	astrocytoma	non-codel	NA	female	NA	Methylated	IDHmut-non-cod	Mutant
TCGA-HT-7877-01A-11R-2403-07	TCGA-HT-7877-01A	oligodendroglioma	codel	NE	female	TERT	Methylated	IDHmut-codel	Mutant
TCGA-S9-A6WD-01A-12R-A33Z-07	TCGA-S9-A6WD-01A	oligodendroglioma	codel	PN	male	NA	Methylated	IDHmut-codel	Mutant
TCGA-QH-A6CS-01A-11R-A31N-07	TCGA-QH-A6CS-01A	astrocytoma	non-codel	NA	male	NA	Unmethylate	IDHwt	WT
TCGA-P5-A77W-01A-11R-A32Q-07	TCGA-P5-A77W-01A	NA	codel	PN	female	NA	Methylated	IDHmut-codel	Mutant
TCGA-DB-A4XG-01A-11R-A27Q-07	TCGA-DB-A4XG-01A	oligodendroglioma	codel	PN	male	TERT	Methylated	IDHmut-codel	Mutant
TCGA-DB-A64L-01A-11R-A29R-07	TCGA-DB-A64L-01A	oligodendroglioma	codel	NE	female	TERT	Methylated	IDHmut-codel	Mutant
TCGA-VM-A8CF-01A-11R-A36H-07	TCGA-VM-A8CF-01A	NA	non-codel	NA	female	NA	Methylated	IDHmut-non-cod	Mutant
TCGA-TM-A7C3-01A-11R-A32Q-07	TCGA-TM-A7C3-01A	astrocytoma	non-codel	CL	female	NA	Methylated	IDHwt	WT
TCGA-CS-6667-01A-12R-2027-07	TCGA-CS-6667-01A	astrocytoma	non-codel	NA	female	-/-	Methylated	IDHmut-non-cod	Mutant
TCGA-CS-6666-01A-11R-1896-07	TCGA-CS-6666-01A	astrocytoma	non-codel	PN	male	ATRX	Methylated	IDHmut-non-cod	Mutant
TCGA-DU-A5TR-01A-11R-A28M-07	TCGA-DU-A5TR-01A	oligoastrocytoma	non-codel	ME	male	ATRX	Methylated	IDHmut-non-cod	Mutant
TCGA-VM-A8C9-01A-11R-A36H-07	TCGA-VM-A8C9-01A	astrocytoma	non-codel	NA	female	NA	Unmethylate	IDHwt	WT
TCGA-RY-A83Z-01A-11R-A36H-07	TCGA-RY-A83Z-01A	NA	non-codel	NA	female	NA	Methylated	IDHmut-non-cod	Mutant
TCGA-F6-A8O3-01A-11R-A36H-07	TCGA-F6-A8O3-01A	oligodendroglioma	codel	NA	male	NA	Methylated	IDHmut-codel	Mutant
TCGA-S9-A89Z-01A-11R-A36H-07	TCGA-S9-A89Z-01A	astrocytoma	non-codel	NA	male	NA	Methylated	IDHmut-non-cod	Mutant
TCGA-DU-6403-01A-11R-1708-07	TCGA-DU-6403-01A	oligoastrocytoma	non-codel	PN	female	TERT	Unmethylate	IDHwt	WT
TCGA-FG-6688-01A-11R-1896-07	TCGA-FG-6688-01A	astrocytoma	non-codel	NA	female	TERT	Methylated	IDHwt	WT
TCGA-DU-A7TC-01A-21R-A34R-07	TCGA-DU-A7TC-01A	astrocytoma	non-codel	NE	male	NA	Methylated	IDHmut-non-cod	Mutant
TCGA-FG-A4MX-01A-11R-A26U-07	TCGA-FG-A4MX-01A	astrocytoma	non-codel	NA	male	ATRX	Methylated	IDHmut-non-cod	Mutant

TCGA-TM-A7C4-01A-11R-A32Q-07	TCGA-TM-A7C4-01A	astrocytoma	non-codel	NE	female	NA	Methylated	IDHmut-non-cod	Mutant
TCGA-S9-A6UB-01A-21R-A33Z-07	TCGA-S9-A6UB-01A	oligodendroglioma	codel	PN	male	NA	Methylated	IDHmut-codel	Mutant
TCGA-HT-7473-01A-11R-2027-07	TCGA-HT-7473-01A	oligoastrocytoma	non-codel	NA	male	ATRX	Unmethylate	IDHmut-non-cod	Mutant
TCGA-TQ-A7RV-01A-21R-A34F-07	TCGA-TQ-A7RV-01A	NA	non-codel	PN	male	ATRX	Methylated	IDHmut-non-cod	Mutant
TCGA-DU-A7TD-01A-12R-A34F-07	TCGA-DU-A7TD-01A	oligoastrocytoma	non-codel	CL	male	NA	Unmethylate	IDHwt	WT
TCGA-FG-8186-01A-11R-2256-07	TCGA-FG-8186-01A	oligoastrocytoma	codel	NA	female	TERT	Methylated	IDHmut-codel	Mutant
TCGA-DU-8161-01A-11R-2256-07	TCGA-DU-8161-01A	oligoastrocytoma	non-codel	CL	female	TERT	Unmethylate	IDHwt	WT
TCGA-QH-A6X9-01A-12R-A32Q-07	TCGA-QH-A6X9-01A	oligodendroglioma	non-codel	PN	female	NA	Methylated	IDHmut-non-cod	Mutant
TCGA-S9-A6U9-01A-11R-A32Q-07	TCGA-S9-A6U9-01A	astrocytoma	non-codel	NA	male	NA	Methylated	IDHmut-non-cod	Mutant
TCGA-HT-8563-01A-11R-2404-07	TCGA-HT-8563-01A	astrocytoma	non-codel	ME	female	ATRX	Unmethylate	IDHmut-non-cod	Mutant
TCGA-DB-A64W-01A-11R-A29R-07	TCGA-DB-A64W-01A	oligoastrocytoma	codel	PN	female	TERT	Methylated	IDHmut-codel	Mutant
TCGA-TQ-A7RN-01A-11R-A33Z-07	TCGA-TQ-A7RN-01A	NA	codel	PN	male	NA	Methylated	IDHmut-codel	Mutant
TCGA-DH-A7UV-01A-12R-A34F-07	TCGA-DH-A7UV-01A	astrocytoma	non-codel	PN	male	NA	Methylated	IDHmut-non-cod	Mutant
TCGA-FG-7637-01A-11R-2090-07	TCGA-FG-7637-01A	oligoastrocytoma	non-codel	PN	male	-/-	Methylated	IDHmut-non-cod	Mutant
TCGA-HT-7874-01A-11R-2403-07	TCGA-HT-7874-01A	oligodendroglioma	codel	NE	female	TERT	Methylated	IDHmut-codel	Mutant
TCGA-DB-5280-01A-01R-1470-07	TCGA-DB-5280-01A	oligoastrocytoma	non-codel	PN	male	ATRX	Methylated	IDHmut-non-cod	Mutant
TCGA-E1-A7YD-01A-11R-A34F-07	TCGA-E1-A7YD-01A	NA	non-codel	CL	male	NA	Unmethylate	IDHwt	WT
TCGA-HT-7858-01A-11R-2403-07	TCGA-HT-7858-01A	astrocytoma	non-codel	PN	male	ATRX	Methylated	IDHmut-non-cod	Mutant
TCGA-HT-7695-01A-11R-2256-07	TCGA-HT-7695-01A	oligodendroglioma	codel	NE	female	TERT	Methylated	IDHmut-codel	Mutant
TCGA-HT-7480-01A-11R-2090-07	TCGA-HT-7480-01A	oligodendroglioma	codel	PN	male	TERT	Methylated	IDHmut-codel	Mutant
TCGA-P5-A5F0-01A-11R-A28M-07	TCGA-P5-A5F0-01A	oligodendroglioma	codel	NE	male	TERT	Methylated	IDHmut-codel	Mutant
TCGA-TM-A84B-01A-11R-A36H-07	TCGA-TM-A84B-01A	astrocytoma	non-codel	NA	male	NA	Unmethylate	IDHwt	WT
TCGA-E1-5322-01A-01R-1470-07	TCGA-E1-5322-01A	oligoastrocytoma	non-codel	PN	female	NA	Methylated	IDHmut-non-cod	Mutant
TCGA-HT-8108-01A-11R-2404-07	TCGA-HT-8108-01A	oligodendroglioma	non-codel	PN	female	ATRX	Methylated	IDHmut-non-cod	Mutant
TCGA-DB-A75M-01A-11R-A32Q-07	TCGA-DB-A75M-01A	astrocytoma	non-codel	NA	male	NA	Methylated	IDHmut-non-cod	Mutant
TCGA-DU-6410-01A-11R-1896-07	TCGA-DU-6410-01A	oligodendroglioma	codel	PN	male	TERT	Methylated	IDHmut-codel	Mutant
TCGA-TM-A84S-01A-11R-A36H-07	TCGA-TM-A84S-01A	oligodendroglioma	codel	NA	male	NA	Methylated	IDHmut-codel	Mutant
TCGA-E1-A7YU-01A-11R-A34R-07	TCGA-E1-A7YU-01A	NA	non-codel	NE	male	NA	Methylated	IDHmut-non-cod	Mutant
TCGA-DU-8167-01A-11R-2256-07	TCGA-DU-8167-01A	oligoastrocytoma	non-codel	PN	female	-/-	Methylated	IDHmut-non-cod	Mutant
TCGA-HT-7879-01A-11R-2403-07	TCGA-HT-7879-01A	oligoastrocytoma	non-codel	PN	male	ATRX	Methylated	IDHmut-non-cod	Mutant
TCGA-HT-7608-01A-11R-2090-07	TCGA-HT-7608-01A	oligoastrocytoma	codel	NE	male	TERT	Methylated	IDHmut-codel	Mutant
TCGA-S9-A7J2-01A-11R-A34F-07	TCGA-S9-A7J2-01A	oligodendroglioma	codel	PN	male	NA	Methylated	IDHmut-codel	Mutant
TCGA-DU-A7TI-01A-11R-A33Z-07	TCGA-DU-A7TI-01A	NA	non-codel	NE	male	NA	Methylated	NA	NA

TCGA-HT-8110-01A-11R-2404-07	TCGA-HT-8110-01A	astrocytoma	non-codel	ME	male	TERT	Methylated	IDHwt	WT
TCGA-HT-A5R5-01A-11R-A28M-07	TCGA-HT-A5R5-01A	oligodendroglioma	non-codel	NE	female	ATRX	Methylated	IDHmut-non-cod	Mutant
TCGA-QH-A6X3-01A-21R-A32Q-07	TCGA-QH-A6X3-01A	oligoastrocytoma	non-codel	PN	male	NA	Methylated	IDHmut-non-cod	Mutant
TCGA-WY-A85E-01A-11R-A36H-07	TCGA-WY-A85E-01A	NA	non-codel	NA	female	NA	Methylated	IDHmut-non-cod	Mutant
TCGA-HT-7855-01A-11R-2403-07	TCGA-HT-7855-01A	astrocytoma	non-codel	PN	male	ATRX	Methylated	IDHmut-non-cod	Mutant
TCGA-HT-7854-01A-11R-2256-07	TCGA-HT-7854-01A	astrocytoma	non-codel	NE	male	TERT	Unmethylate	IDHwt	WT
TCGA-HW-8321-01A-11R-2404-07	TCGA-HW-8321-01A	astrocytoma	non-codel	PN	male	-/-	Methylated	IDHmut-non-cod	Mutant
TCGA-R8-A6MK-01A-11R-A32Q-07	TCGA-R8-A6MK-01A	oligodendroglioma	codel	NA	male	NA	Methylated	IDHmut-codel	Mutant
TCGA-HT-7902-01A-12R-2403-07	TCGA-HT-7902-01A	oligoastrocytoma	non-codel	NE	female	ATRX	Methylated	IDHmut-non-cod	Mutant
TCGA-DU-A7TG-01A-21R-A34R-07	TCGA-DU-A7TG-01A	oligodendroglioma	non-codel	NE	male	NA	Methylated	IDHmut-non-cod	Mutant
TCGA-TQ-A7RJ-01A-11R-A33Z-07	TCGA-TQ-A7RJ-01A	NA	non-codel	NA	female	NA	Methylated	IDHmut-non-cod	Mutant
TCGA-FN-7833-01A-11R-2090-07	TCGA-FN-7833-01A	oligoastrocytoma	non-codel	NA	male	ATRX	Methylated	IDHmut-non-cod	Mutant
TCGA-RY-A83Y-01A-11R-A36H-07	TCGA-RY-A83Y-01A	NA	codel	NA	male	NA	Methylated	IDHmut-codel	Mutant
TCGA-KT-A74X-01A-11R-A32Q-07	TCGA-KT-A74X-01A	oligoastrocytoma	codel	PN	male	NA	Methylated	IDHmut-codel	Mutant
TCGA-TM-A84M-01A-11R-A36H-07	TCGA-TM-A84M-01A	oligodendroglioma	codel	NA	male	NA	Methylated	IDHmut-codel	Mutant
TCGA-DU-5852-01A-11R-1708-07	TCGA-DU-5852-01A	oligoastrocytoma	non-codel	CL	female	NA	Methylated	IDHwt	WT
TCGA-FG-5963-02A-12R-A29R-07	TCGA-FG-5963-02A	NA	NA	NA	male	NA	NA	NA	NA
TCGA-DU-6392-01A-11R-1708-07	TCGA-DU-6392-01A	astrocytoma	non-codel	PN	female	NA	Unmethylate	IDHwt	WT
TCGA-S9-A7R7-01A-11R-A34R-07	TCGA-S9-A7R7-01A	astrocytoma	non-codel	ME	male	NA	Methylated	IDHmut-non-cod	Mutant
TCGA-HT-8564-01A-11R-2404-07	TCGA-HT-8564-01A	astrocytoma	non-codel	NE	male	-/-	Unmethylate	IDHwt	WT
TCGA-QH-A6CU-01A-11R-A31N-07	TCGA-QH-A6CU-01A	oligodendroglioma	codel	NA	female	NA	Methylated	IDHmut-codel	Mutant
TCGA-DU-6393-01A-11R-1708-07	TCGA-DU-6393-01A	oligodendroglioma	codel	PN	male	TERT	Methylated	IDHmut-codel	Mutant
TCGA-TQ-A7RP-01A-21R-A34F-07	TCGA-TQ-A7RP-01A	NA	non-codel	NE	male	NA	Methylated	IDHwt	WT
TCGA-HT-7476-01A-11R-2027-07	TCGA-HT-7476-01A	astrocytoma	non-codel	NE	male	ATRX	Methylated	IDHmut-non-cod	Mutant
TCGA-DU-5853-01A-11R-1896-07	TCGA-DU-5853-01A	oligoastrocytoma	non-codel	PN	male	ATRX	Methylated	IDHmut-non-cod	Mutant
TCGA-DB-A64R-01A-11R-A29R-07	TCGA-DB-A64R-01A	oligodendroglioma	codel	PN	female	TERT	Methylated	IDHmut-codel	Mutant
TCGA-HT-7604-01A-11R-2090-07	TCGA-HT-7604-01A	astrocytoma	non-codel	PN	male	ATRX	Methylated	IDHmut-non-cod	Mutant
TCGA-TQ-A7RK-02A-11R-A36H-07	TCGA-TQ-A7RK-02A	NA	NA	NA	male	NA	NA	NA	NA
TCGA-DB-A64Q-01A-11R-A29R-07	TCGA-DB-A64Q-01A	oligoastrocytoma	codel	PN	female	TERT	Methylated	IDHmut-codel	Mutant
TCGA-FG-A713-01A-11R-A32Q-07	TCGA-FG-A713-01A	oligoastrocytoma	codel	NE	female	NA	Methylated	IDHmut-codel	Mutant
TCGA-FG-6691-01A-11R-1896-07	TCGA-FG-6691-01A	astrocytoma	non-codel	PN	female	ATRX	Unmethylate	IDHmut-non-cod	Mutant
TCGA-FG-A87Q-01A-11R-A36H-07	TCGA-FG-A87Q-01A	astrocytoma	non-codel	NA	female	NA	Methylated	IDHwt	WT
TCGA-DH-A66G-01A-21R-A31N-07	TCGA-DH-A66G-01A	oligodendroglioma	non-codel	NA	female	NA	Methylated	IDHmut-non-cod	Mutant

TCGA-FG-A60L-01A-12R-A31N-07	TCGA-FG-A60L-01A	astrocytoma	non-codel	NA	female	NA	Methylated	IDHmut-non-cod	Mutant
TCGA-HT-7857-01A-11R-2403-07	TCGA-HT-7857-01A	astrocytoma	non-codel	ME	female	ATRX	Unmethylate	IDHwt	WT
TCGA-S9-A7R1-01A-12R-A34R-07	TCGA-S9-A7R1-01A	oligodendroglioma	codel	NE	male	NA	Methylated	IDHmut-codel	Mutant
TCGA-HT-7616-01A-11R-2256-07	TCGA-HT-7616-01A	oligodendroglioma	codel	ME	male	TERT	Methylated	IDHmut-codel	Mutant
TCGA-HT-7474-01A-11R-2027-07	TCGA-HT-7474-01A	oligoastrocytoma	non-codel	NE	male	ATRX	Methylated	IDHmut-non-cod	Mutant
TCGA-WY-A859-01A-12R-A36H-07	TCGA-WY-A859-01A	NA	non-codel	NA	female	NA	Methylated	IDHmut-non-cod	Mutant
TCGA-P5-A737-01A-11R-A32Q-07	TCGA-P5-A737-01A	NA	codel	PN	male	NA	Methylated	IDHmut-codel	Mutant
TCGA-QH-A6X8-01A-12R-A32Q-07	TCGA-QH-A6X8-01A	oligodendroglioma	codel	PN	female	NA	Methylated	IDHmut-codel	Mutant
TCGA-DU-8166-01A-11R-2256-07	TCGA-DU-8166-01A	oligoastrocytoma	non-codel	PN	female	ATRX	Methylated	IDHmut-non-cod	Mutant
TCGA-S9-A7IY-01A-11R-A34F-07	TCGA-S9-A7IY-01A	oligoastrocytoma	codel	NA	male	NA	Methylated	IDHmut-codel	Mutant
TCGA-P5-A72U-01A-31R-A32Q-07	TCGA-P5-A72U-01A	oligodendroglioma	non-codel	CL	female	NA	Methylated	IDHwt	WT
TCGA-HT-7481-01A-11R-2027-07	TCGA-HT-7481-01A	oligodendroglioma	codel	NE	male	TERT	Methylated	IDHmut-codel	Mutant
TCGA-DU-A7TJ-01A-11R-A34R-07	TCGA-DU-A7TJ-01A	astrocytoma	non-codel	CL	male	NA	Methylated	IDHwt	WT
TCGA-DH-A7UT-01A-12R-A34F-07	TCGA-DH-A7UT-01A	astrocytoma	non-codel	NA	male	NA	Methylated	IDHmut-non-cod	Mutant
TCGA-FG-7636-01A-11R-2090-07	TCGA-FG-7636-01A	astrocytoma	non-codel	PN	male	ATRX	Methylated	IDHmut-non-cod	Mutant
TCGA-DU-A6S8-01A-12R-A32Q-07	TCGA-DU-A6S8-01A	oligodendroglioma	codel	PN	female	NA	Methylated	IDHmut-codel	Mutant
TCGA-DU-5874-01A-11R-1708-07	TCGA-DU-5874-01A	oligodendroglioma	codel	PN	female	TERT	Methylated	IDHmut-codel	Mutant
TCGA-HT-8018-01A-11R-2404-07	TCGA-HT-8018-01A	oligoastrocytoma	non-codel	NE	female	-/-	Methylated	IDHmut-non-cod	Mutant
TCGA-DU-7012-01A-11R-2027-07	TCGA-DU-7012-01A	astrocytoma	non-codel	ME	female	TERT	Methylated	IDHwt	WT
TCGA-FG-A6J1-01A-11R-A31N-07	TCGA-FG-A6J1-01A	oligodendroglioma	codel	PN	female	NA	Methylated	IDHmut-codel	Mutant
TCGA-S9-A7IS-01A-11R-A34F-07	TCGA-S9-A7IS-01A	astrocytoma	non-codel	PN	female	NA	Methylated	IDHmut-non-cod	Mutant
TCGA-DH-5143-01A-01R-1470-07	TCGA-DH-5143-01A	oligoastrocytoma	non-codel	NE	male	-/-	Methylated	IDHmut-non-cod	Mutant
TCGA-P5-A733-01A-11R-A32Q-07	TCGA-P5-A733-01A	astrocytoma	non-codel	NA	female	NA	Methylated	IDHmut-non-cod	Mutant
TCGA-DH-A66F-01A-11R-A29R-07	TCGA-DH-A66F-01A	oligodendroglioma	codel	PN	male	TERT	Methylated	IDHmut-codel	Mutant
TCGA-DU-A5TP-01A-11R-A28M-07	TCGA-DU-A5TP-01A	astrocytoma	non-codel	ME	male	ATRX	Methylated	IDHmut-non-cod	Mutant
TCGA-HT-8105-01A-11R-2404-07	TCGA-HT-8105-01A	oligodendroglioma	codel	PN	male	TERT	Methylated	IDHmut-codel	Mutant
TCGA-DU-6407-02A-12R-A36H-07	TCGA-DU-6407-02A	NA	NA	NA	female	NA	NA	NA	NA
TCGA-CS-6668-01A-11R-1896-07	TCGA-CS-6668-01A	oligodendroglioma	codel	PN	female	TERT	Methylated	IDHmut-codel	Mutant
TCGA-HT-7483-01A-11R-2027-07	TCGA-HT-7483-01A	oligoastrocytoma	non-codel	PN	male	ATRX	Unmethylate	IDHmut-non-cod	Mutant
TCGA-HT-7694-01A-11R-2256-07	TCGA-HT-7694-01A	oligodendroglioma	codel	NE	male	TERT	Methylated	IDHmut-codel	Mutant
TCGA-HT-A614-01A-11R-A29R-07	TCGA-HT-A614-01A	oligoastrocytoma	non-codel	PN	male	ATRX	Methylated	IDHmut-non-cod	Mutant
TCGA-IK-7675-01A-11R-2090-07	TCGA-IK-7675-01A	oligodendroglioma	non-codel	PN	male	ATRX	Methylated	IDHmut-non-cod	Mutant
TCGA-DB-A64S-01A-11R-A29R-07	TCGA-DB-A64S-01A	oligoastrocytoma	non-codel	NA	male	ATRX	Unmethylate	IDHmut-non-cod	Mutant

TCGA-FG-A6J3-01A-11R-A31N-07	TCGA-FG-A6J3-01A	astrocytoma	non-codel	ME	female	NA	Methylated	IDHmut-non-cod	Mutant
TCGA-DU-6397-02A-12R-A36H-07	TCGA-DU-6397-02A	NA	NA	NA	male	NA	NA	NA	NA
TCGA-TQ-A7RF-01A-11R-A33Z-07	TCGA-TQ-A7RF-01A	NA	non-codel	NA	female	NA	Unmethylate	IDHmut-non-cod	Mutant
TCGA-QH-A6CX-01A-11R-A32Q-07	TCGA-QH-A6CX-01A	astrocytoma	non-codel	CL	male	NA	Unmethylate	IDHwt	WT
TCGA-TM-A84L-01A-11R-A36H-07	TCGA-TM-A84L-01A	oligoastrocytoma	non-codel	NA	male	NA	Methylated	IDHmut-non-cod	Mutant
TCGA-E1-A7Z2-01A-21R-A34R-07	TCGA-E1-A7Z2-01A	NA	non-codel	NE	female	NA	Unmethylate	IDHwt	WT
TCGA-HT-7472-01A-11R-2027-07	TCGA-HT-7472-01A	oligodendroglioma	non-codel	PN	male	ATRX	Methylated	IDHmut-non-cod	Mutant
TCGA-DB-A4X9-01A-11R-A26U-07	TCGA-DB-A4X9-01A	oligoastrocytoma	non-codel	PN	female	-/-	Methylated	IDHmut-non-cod	Mutant
TCGA-DU-6397-01A-11R-1708-07	TCGA-DU-6397-01A	oligodendroglioma	codel	PN	male	TERT	Methylated	IDHmut-codel	Mutant
TCGA-TQ-A7RR-01A-21R-A34F-07	TCGA-TQ-A7RR-01A	NA	non-codel	NE	male	NA	Unmethylate	IDHmut-non-cod	Mutant
TCGA-DU-5847-01A-11R-1708-07	TCGA-DU-5847-01A	astrocytoma	non-codel	ME	female	TERT	Methylated	IDHwt	WT
TCGA-HT-7609-01A-11R-2090-07	TCGA-HT-7609-01A	oligoastrocytoma	non-codel	PN	male	-/-	Methylated	IDHmut-non-cod	Mutant
TCGA-E1-A7YI-01A-11R-A34F-07	TCGA-E1-A7YI-01A	NA	non-codel	PN	female	NA	Methylated	IDHmut-non-cod	Mutant
TCGA-DB-A4XB-01A-11R-A26U-07	TCGA-DB-A4XB-01A	astrocytoma	non-codel	PN	male	-/-	Methylated	IDHmut-non-cod	Mutant
TCGA-QH-A6CW-01A-11R-A32Q-07	TCGA-QH-A6CW-01A	oligoastrocytoma	non-codel	NA	male	NA	Methylated	IDHmut-non-cod	Mutant
TCGA-E1-A7Z6-01A-11R-A34R-07	TCGA-E1-A7Z6-01A	astrocytoma	non-codel	NA	female	NA	Methylated	IDHmut-non-cod	Mutant
TCGA-S9-A6TS-01A-12R-A33Z-07	TCGA-S9-A6TS-01A	astrocytoma	non-codel	NE	female	NA	Methylated	IDHmut-non-cod	Mutant
TCGA-FG-5965-02A-11R-A29R-07	TCGA-FG-5965-02A	NA	NA	NA	female	NA	NA	NA	NA
TCGA-S9-A89V-01A-11R-A36H-07	TCGA-S9-A89V-01A	astrocytoma	non-codel	NA	male	NA	Methylated	IDHwt	WT
TCGA-TM-A7C5-01A-11R-A32Q-07	TCGA-TM-A7C5-01A	oligoastrocytoma	codel	PN	male	NA	Methylated	IDHmut-codel	Mutant
TCGA-DH-A7US-01A-11R-A33Z-07	TCGA-DH-A7US-01A	oligodendroglioma	codel	PN	male	NA	Methylated	IDHmut-codel	Mutant
TCGA-P5-A736-01A-11R-A32Q-07	TCGA-P5-A736-01A	NA	non-codel	NA	female	NA	Methylated	IDHmut-non-cod	Mutant
TCGA-DU-6394-01A-11R-1708-07	TCGA-DU-6394-01A	oligodendroglioma	codel	PN	male	TERT	Methylated	IDHmut-codel	Mutant
TCGA-HT-A74K-01A-11R-A32Q-07	TCGA-HT-A74K-01A	NA	codel	PN	female	NA	Methylated	IDHmut-codel	Mutant
TCGA-TQ-A7RI-01A-11R-A33Z-07	TCGA-TQ-A7RI-01A	NA	codel	PN	female	NA	Methylated	IDHmut-codel	Mutant
TCGA-DU-6400-01A-12R-1708-07	TCGA-DU-6400-01A	oligodendroglioma	codel	PN	female	TERT	Methylated	IDHmut-codel	Mutant
TCGA-S9-A6WP-01A-12R-A34F-07	TCGA-S9-A6WP-01A	oligoastrocytoma	codel	PN	male	NA	Methylated	IDHmut-codel	Mutant
TCGA-P5-A781-01A-11R-A32Q-07	TCGA-P5-A781-01A	NA	codel	NE	female	NA	Methylated	IDHmut-codel	Mutant
TCGA-DU-A7T6-01A-11R-A33Z-07	TCGA-DU-A7T6-01A	oligodendroglioma	codel	PN	female	NA	Methylated	IDHmut-codel	Mutant
TCGA-DH-A669-01A-12R-A31N-07	TCGA-DH-A669-01A	oligodendroglioma	codel	PN	male	TERT	Methylated	IDHmut-codel	Mutant
TCGA-P5-A730-01A-11R-A32Q-07	TCGA-P5-A730-01A	oligoastrocytoma	codel	NA	male	NA	Methylated	IDHmut-codel	Mutant
TCGA-HT-7478-01A-11R-2027-07	TCGA-HT-7478-01A	astrocytoma	non-codel	ME	male	ATRX	Unmethylate	IDHmut-non-cod	Mutant
TCGA-S9-A6WM-01A-12R-A33Z-07	TCGA-S9-A6WM-01A	astrocytoma	non-codel	CL	female	NA	Unmethylate	IDHwt	WT

TCGA-TQ-A7RW-01A-11R-A33Z-07	TCGA-TQ-A7RW-01A	NA	non-codel	PN	male	NA	Methylated	IDHmut-non-cod	Mutant
TCGA-E1-A7Z3-01A-11R-A34R-07	TCGA-E1-A7Z3-01A	NA	non-codel	NE	female	NA	Methylated	IDHmut-non-cod	Mutant
TCGA-DU-8168-01A-11R-2256-07	TCGA-DU-8168-01A	oligodendroglioma	codel	PN	female	TERT	Methylated	IDHmut-codel	Mutant
TCGA-P5-A5F1-01A-11R-A28M-07	TCGA-P5-A5F1-01A	astrocytoma	non-codel	NA	male	ATRX	Unmethylate	IDHmut-non-cod	Mutant
TCGA-HT-7880-01A-11R-2403-07	TCGA-HT-7880-01A	oligoastrocytoma	non-codel	NE	male	ATRX	Methylated	IDHmut-non-cod	Mutant
TCGA-TQ-A7RG-01A-11R-A33Z-07	TCGA-TQ-A7RG-01A	NA	codel	PN	male	NA	Methylated	IDHmut-codel	Mutant
TCGA-HT-7692-01A-12R-2256-07	TCGA-HT-7692-01A	oligoastrocytoma	codel	PN	male	TERT	Methylated	IDHmut-codel	Mutant
TCGA-HT-8558-01A-21R-2404-07	TCGA-HT-8558-01A	oligodendroglioma	non-codel	NE	female	-/-	Unmethylate	IDHwt	WT
TCGA-P5-A5F6-01A-11R-A28M-07	TCGA-P5-A5F6-01A	oligodendroglioma	non-codel	ME	male	-/-	Unmethylate	IDHwt	WT
TCGA-DH-5142-01A-01R-1470-07	TCGA-DH-5142-01A	astrocytoma	non-codel	PN	male	ATRX	Methylated	IDHmut-non-cod	Mutant
TCGA-DB-5273-01A-01R-1470-07	TCGA-DB-5273-01A	astrocytoma	non-codel	NA	male	ATRX	Unmethylate	IDHmut-non-cod	Mutant
TCGA-FG-A4MT-02A-11R-A29R-07	TCGA-FG-A4MT-02A	NA	NA	NA	female	NA	NA	NA	NA
TCGA-DU-A7TA-01A-11R-A33Z-07	TCGA-DU-A7TA-01A	oligodendroglioma	non-codel	PN	male	NA	Methylated	IDHmut-non-cod	Mutant
TCGA-RY-A83X-01A-11R-A36H-07	TCGA-RY-A83X-01A	NA	codel	NA	female	NA	Methylated	IDHmut-codel	Mutant
TCGA-HT-7610-01A-21R-2090-07	TCGA-HT-7610-01A	oligoastrocytoma	non-codel	NE	female	ATRX	Methylated	IDHmut-non-cod	Mutant
TCGA-DU-A5TY-01A-11R-A28M-07	TCGA-DU-A5TY-01A	astrocytoma	non-codel	CL	female	TERT	Methylated	IDHwt	WT
TCGA-P5-A72W-01A-11R-A32Q-07	TCGA-P5-A72W-01A	astrocytoma	non-codel	PN	male	NA	Unmethylate	IDHmut-non-cod	Mutant
TCGA-QH-A6XA-01A-12R-A32Q-07	TCGA-QH-A6XA-01A	oligoastrocytoma	non-codel	PN	female	NA	Methylated	IDHmut-non-cod	Mutant
TCGA-S9-A6U2-01A-21R-A33Z-07	TCGA-S9-A6U2-01A	oligodendroglioma	codel	PN	female	NA	Methylated	IDHmut-codel	Mutant
TCGA-TM-A84H-01A-11R-A36H-07	TCGA-TM-A84H-01A	oligoastrocytoma	non-codel	NA	female	NA	Methylated	IDHmut-non-cod	Mutant
TCGA-DB-A64P-01A-11R-A29R-07	TCGA-DB-A64P-01A	oligodendroglioma	codel	PN	male	TERT	Methylated	IDHmut-codel	Mutant
TCGA-HT-A74O-01A-11R-A32Q-07	TCGA-HT-A74O-01A	astrocytoma	non-codel	NA	male	NA	Methylated	IDHmut-non-cod	Mutant
TCGA-FG-8185-01A-11R-2256-07	TCGA-FG-8185-01A	astrocytoma	non-codel	PN	male	ATRX	Methylated	IDHmut-non-cod	Mutant
TCGA-E1-5307-01A-01R-1896-07	TCGA-E1-5307-01A	astrocytoma	non-codel	PN	female	ATRX	Methylated	IDHmut-non-cod	Mutant
TCGA-DU-7013-01A-11R-2027-07	TCGA-DU-7013-01A	astrocytoma	non-codel	CL	male	TERT	Unmethylate	IDHwt	WT
TCGA-DU-6399-01A-12R-1708-07	TCGA-DU-6399-01A	oligodendroglioma	non-codel	PN	male	ATRX	Methylated	IDHmut-non-cod	Mutant
TCGA-HT-7611-01A-11R-2403-07	TCGA-HT-7611-01A	oligoastrocytoma	non-codel	NA	male	ATRX	Methylated	IDHmut-non-cod	Mutant
TCGA-DB-A4XA-01A-11R-A26U-07	TCGA-DB-A4XA-01A	oligoastrocytoma	codel	PN	male	TERT	Methylated	IDHmut-codel	Mutant
TCGA-HT-A5R9-01A-11R-A28M-07	TCGA-HT-A5R9-01A	oligodendroglioma	codel	PN	female	TERT	Methylated	IDHmut-codel	Mutant
TCGA-DB-A4XC-01A-11R-A26U-07	TCGA-DB-A4XC-01A	oligoastrocytoma	non-codel	NE	male	ATRX	Methylated	IDHmut-non-cod	Mutant
TCGA-E1-5318-01A-01R-1470-07	TCGA-E1-5318-01A	oligodendroglioma	codel	PN	female	TERT	Methylated	IDHmut-codel	Mutant
TCGA-DU-6402-01A-11R-1708-07	TCGA-DU-6402-01A	astrocytoma	non-codel	CL	male	TERT	Unmethylate	IDHwt	WT
TCGA-WY-A85D-01A-11R-A36H-07	TCGA-WY-A85D-01A	NA	non-codel	NA	male	NA	Unmethylate	IDHmut-non-cod	Mutant

TCGA-FG-5963-01A-11R-1708-07	TCGA-FG-5963-01A	astrocytoma	non-codel	ME	male	ATRX	Unmethylate	IDHwt	WT
TCGA-DU-7007-01A-11R-2027-07	TCGA-DU-7007-01A	astrocytoma	non-codel	ME	male	ATRX	Methylated	IDHmut-non-cod	Mutant
TCGA-DB-A64X-01A-11R-A29R-07	TCGA-DB-A64X-01A	astrocytoma	non-codel	NA	female	-/-	Methylated	IDHmut-non-cod	Mutant
TCGA-DH-A669-02A-11R-A31N-07	TCGA-DH-A669-02A	NA	NA	NA	male	NA	NA	NA	NA
TCGA-VM-A8CB-01A-11R-A36H-07	TCGA-VM-A8CB-01A	oligodendroglioma	codel	NA	male	NA	Methylated	IDHmut-codel	Mutant
TCGA-HT-7856-01A-11R-2403-07	TCGA-HT-7856-01A	oligodendroglioma	codel	NE	male	TERT	Methylated	IDHmut-codel	Mutant
TCGA-HW-A5KL-01A-11R-A27Q-07	TCGA-HW-A5KL-01A	astrocytoma	non-codel	PN	female	ATRX	Methylated	IDHmut-non-cod	Mutant
TCGA-TM-A7CF-02A-11R-A32Q-07	TCGA-TM-A7CF-02A	NA	NA	NA	female	NA	NA	NA	NA
TCGA-HT-8015-01B-11R-A28M-07	TCGA-HT-8015-01B	astrocytoma	non-codel	NE	male	-/-	Unmethylate	IDHwt	WT
TCGA-S9-A6TU-01A-12R-A32Q-07	TCGA-S9-A6TU-01A	astrocytoma	non-codel	PN	male	NA	Methylated	IDHmut-non-cod	Mutant
TCGA-HT-7602-01A-21R-2090-07	TCGA-HT-7602-01A	oligodendroglioma	non-codel	NA	male	-/-	Methylated	IDHmut-non-cod	Mutant
TCGA-CS-5396-01A-02R-1470-07	TCGA-CS-5396-01A	oligodendroglioma	codel	PN	female	TERT	Methylated	IDHmut-codel	Mutant
TCGA-S9-A6WG-01A-11R-A33Z-07	TCGA-S9-A6WG-01A	astrocytoma	non-codel	ME	male	NA	Methylated	IDHmut-non-cod	Mutant
TCGA-S9-A6WQ-01A-12R-A34F-07	TCGA-S9-A6WQ-01A	oligoastrocytoma	non-codel	PN	female	NA	Methylated	IDHmut-non-cod	Mutant
TCGA-HT-7606-01A-11R-2090-07	TCGA-HT-7606-01A	astrocytoma	non-codel	PN	female	-/-	Unmethylate	IDHmut-non-cod	Mutant
TCGA-HT-7470-01A-12R-2090-07	TCGA-HT-7470-01A	oligodendroglioma	non-codel	NE	male	ATRX	Methylated	IDHmut-non-cod	Mutant
TCGA-HT-7468-01A-11R-2027-07	TCGA-HT-7468-01A	oligodendroglioma	codel	PN	male	TERT	Methylated	IDHmut-codel	Mutant
TCGA-HT-7875-01A-11R-2403-07	TCGA-HT-7875-01A	oligodendroglioma	codel	PN	male	TERT	Methylated	IDHmut-codel	Mutant
TCGA-VM-A8CA-01A-11R-A36H-07	TCGA-VM-A8CA-01A	oligodendroglioma	non-codel	NA	male	NA	Methylated	IDHmut-non-cod	Mutant
TCGA-FG-7643-01A-11R-2090-07	TCGA-FG-7643-01A	oligoastrocytoma	non-codel	NE	female	TERT	Methylated	IDHwt	WT
TCGA-DU-6404-01A-11R-1708-07	TCGA-DU-6404-01A	oligodendroglioma	non-codel	CL	female	-/-	Unmethylate	IDHwt	WT
TCGA-TM-A84F-01A-11R-A36H-07	TCGA-TM-A84F-01A	astrocytoma	non-codel	NA	male	NA	Methylated	IDHmut-non-cod	Mutant
TCGA-P5-A731-01A-11R-A32Q-07	TCGA-P5-A731-01A	oligoastrocytoma	non-codel	NE	female	NA	Methylated	IDHmut-non-cod	Mutant
TCGA-E1-5304-01A-01R-1470-07	TCGA-E1-5304-01A	astrocytoma	non-codel	PN	male	ATRX	Unmethylate	IDHmut-non-cod	Mutant
TCGA-E1-A7YQ-01A-11R-A34R-07	TCGA-E1-A7YQ-01A	NA	non-codel	ME	female	NA	Unmethylate	IDHwt	WT
TCGA-DU-A7TB-01A-11R-A33Z-07	TCGA-DU-A7TB-01A	oligodendroglioma	non-codel	PN	male	NA	Unmethylate	IDHwt	WT
TCGA-HT-7485-01A-11R-2027-07	TCGA-HT-7485-01A	astrocytoma	non-codel	NA	male	ATRX	Methylated	IDHmut-non-cod	Mutant
TCGA-HT-A5R7-01A-11R-A28M-07	TCGA-HT-A5R7-01A	astrocytoma	non-codel	NE	female	ATRX	Methylated	IDHmut-non-cod	Mutant
TCGA-FG-6690-01A-11R-1896-07	TCGA-FG-6690-01A	oligodendroglioma	non-codel	PN	male	ATRX	Methylated	IDHmut-non-cod	Mutant
TCGA-DU-7292-01A-11R-2027-07	TCGA-DU-7292-01A	astrocytoma	non-codel	NE	male	-/-	Methylated	IDHwt	WT
TCGA-HT-7680-01A-11R-2256-07	TCGA-HT-7680-01A	astrocytoma	non-codel	NA	female	-/-	Unmethylate	IDHwt	WT
TCGA-CS-6670-01A-11R-1896-07	TCGA-CS-6670-01A	oligodendroglioma	codel	PN	male	NA	Methylated	IDHmut-codel	Mutant
TCGA-HT-7690-01A-11R-2256-07	TCGA-HT-7690-01A	oligoastrocytoma	non-codel	PN	male	ATRX	Methylated	IDHmut-non-cod	Mutant

TCGA-DU-7011-01A-11R-2027-07	TCGA-DU-7011-01A	oligoastrocytoma	non-codel	NE	male	NA	Unmethylate	IDHmut-non-cod	Mutant
TCGA-DB-A75K-01A-11R-A32Q-07	TCGA-DB-A75K-01A	oligoastrocytoma	codel	PN	female	NA	Methylated	IDHmut-codel	Mutant
TCGA-TQ-A7RM-01A-11R-A33Z-07	TCGA-TQ-A7RM-01A	NA	non-codel	PN	female	NA	Methylated	IDHmut-non-cod	Mutant
TCGA-DH-A66B-01A-11R-A29R-07	TCGA-DH-A66B-01A	astrocytoma	non-codel	NA	male	-/-	Methylated	IDHmut-non-cod	Mutant
TCGA-DB-A4XE-01A-11R-A27Q-07	TCGA-DB-A4XE-01A	oligoastrocytoma	non-codel	PN	female	ATRX	Methylated	IDHmut-non-cod	Mutant
TCGA-E1-A7YY-01A-11R-A34R-07	TCGA-E1-A7YY-01A	NA	non-codel	NE	female	NA	Methylated	IDHmut-non-cod	Mutant
TCGA-TQ-A7RH-01A-12R-A34F-07	TCGA-TQ-A7RH-01A	NA	non-codel	PN	male	NA	Methylated	IDHmut-non-cod	Mutant
TCGA-DU-6406-01A-11R-1708-07	TCGA-DU-6406-01A	oligoastrocytoma	non-codel	CL	female	NA	Unmethylate	IDHwt	WT
TCGA-DU-7014-01A-11R-2027-07	TCGA-DU-7014-01A	oligodendroglioma	non-codel	PN	male	NA	Methylated	NA	NA
TCGA-P5-A735-01A-11R-A32Q-07	TCGA-P5-A735-01A	NA	non-codel	PN	female	NA	Methylated	IDHmut-non-cod	Mutant
TCGA-FG-A6IZ-01A-11R-A31N-07	TCGA-FG-A6IZ-01A	oligodendroglioma	codel	PN	male	NA	Methylated	IDHmut-codel	Mutant
TCGA-S9-A6U1-01A-21R-A33Z-07	TCGA-S9-A6U1-01A	astrocytoma	non-codel	PN	female	NA	Methylated	IDHmut-non-cod	Mutant
TCGA-E1-5302-01A-01R-1470-07	TCGA-E1-5302-01A	astrocytoma	non-codel	PN	male	ATRX	Methylated	IDHmut-non-cod	Mutant
TCGA-TM-A84Q-01A-11R-A36H-07	TCGA-TM-A84Q-01A	astrocytoma	non-codel	NA	male	NA	Methylated	IDHmut-non-cod	Mutant
TCGA-DU-6395-01A-13R-1708-07	TCGA-DU-6395-01A	oligoastrocytoma	non-codel	PN	male	NA	Methylated	IDHmut-non-cod	Mutant
TCGA-R8-A73M-01A-11R-A32Q-07	TCGA-R8-A73M-01A	oligodendroglioma	codel	PN	female	NA	Methylated	IDHmut-codel	Mutant
TCGA-DH-A66D-01A-11R-A31N-07	TCGA-DH-A66D-01A	astrocytoma	non-codel	PN	female	NA	Methylated	IDHmut-non-cod	Mutant
TCGA-HT-8104-01A-11R-2404-07	TCGA-HT-8104-01A	astrocytoma	non-codel	CL	female	TERT	Unmethylate	IDHwt	WT
TCGA-HT-8012-01A-11R-2403-07	TCGA-HT-8012-01A	oligodendroglioma	codel	PN	female	TERT	Methylated	IDHmut-codel	Mutant
TCGA-TM-A84G-01A-11R-A36H-07	TCGA-TM-A84G-01A	oligodendroglioma	codel	NA	female	NA	Methylated	IDHmut-codel	Mutant
TCGA-P5-A72X-01A-11R-A32Q-07	TCGA-P5-A72X-01A	astrocytoma	non-codel	NA	male	NA	Methylated	IDHmut-non-cod	Mutant
TCGA-HW-7489-01A-11R-2027-07	TCGA-HW-7489-01A	oligoastrocytoma	non-codel	NE	male	ATRX	Methylated	IDHmut-non-cod	Mutant
TCGA-KT-A7W1-01A-11R-A34F-07	TCGA-KT-A7W1-01A	astrocytoma	non-codel	CL	female	NA	Methylated	IDHwt	WT
TCGA-DU-7015-01A-11R-2027-07	TCGA-DU-7015-01A	oligodendroglioma	non-codel	PN	female	ATRX	Methylated	IDHmut-non-cod	Mutant
TCGA-HT-7686-01A-11R-2256-07	TCGA-HT-7686-01A	astrocytoma	non-codel	NA	female	ATRX	Methylated	IDHmut-non-cod	Mutant
TCGA-VW-A8FI-01A-11R-A36H-07	TCGA-VW-A8FI-01A	astrocytoma	non-codel	NA	male	NA	Unmethylate	IDHwt	WT
TCGA-DU-7304-01A-12R-2090-07	TCGA-DU-7304-01A	oligoastrocytoma	non-codel	NE	male	ATRX	Methylated	IDHmut-non-cod	Mutant
TCGA-S9-A6U6-01A-12R-A33Z-07	TCGA-S9-A6U6-01A	astrocytoma	non-codel	NE	male	NA	Methylated	IDHmut-non-cod	Mutant
TCGA-DB-5279-01A-03R-2347-07	TCGA-DB-5279-01A	oligodendroglioma	codel	NE	male	TERT	Methylated	IDHmut-codel	Mutant
TCGA-VM-A8CE-01A-11R-A36H-07	TCGA-VM-A8CE-01A	oligodendroglioma	codel	NA	male	NA	Methylated	IDHmut-codel	Mutant
TCGA-DU-A5TU-01A-11R-A28M-07	TCGA-DU-A5TU-01A	astrocytoma	non-codel	PN	female	ATRX	Methylated	IDHmut-non-cod	Mutant
TCGA-HT-A618-01A-11R-A29R-07	TCGA-HT-A618-01A	astrocytoma	non-codel	NA	female	ATRX	Methylated	IDHmut-non-cod	Mutant
TCGA-P5-A5F2-01A-11R-A28M-07	TCGA-P5-A5F2-01A	astrocytoma	non-codel	NE	female	ATRX	Methylated	IDHmut-non-cod	Mutant

TCGA-FG-A70Y-01A-12R-A34R-07	TCGA-FG-A70Y-01A	oligodendroglioma	non-codel	NA	female	NA	Methylated	IDHmut-non-cod	Mutant
TCGA-QH-A6CY-01A-11R-A32Q-07	TCGA-QH-A6CY-01A	oligoastrocytoma	codel	NE	male	NA	Methylated	IDHmut-codel	Mutant
TCGA-WY-A85A-01A-21R-A36H-07	TCGA-WY-A85A-01A	NA	non-codel	NA	male	NA	Methylated	IDHmut-non-cod	Mutant
TCGA-VM-A8CD-01A-11R-A36H-07	TCGA-VM-A8CD-01A	astrocytoma	non-codel	NA	male	NA	Unmethylate	IDHwt	WT
TCGA-E1-5303-01A-01R-1470-07	TCGA-E1-5303-01A	astrocytoma	non-codel	NA	male	ATRX	Methylated	IDHmut-non-cod	Mutant
TCGA-HT-7482-01A-11R-2027-07	TCGA-HT-7482-01A	oligoastrocytoma	non-codel	PN	female	ATRX	Methylated	IDHmut-non-cod	Mutant
TCGA-QH-A6CV-01A-11R-A31N-07	TCGA-QH-A6CV-01A	oligoastrocytoma	non-codel	CL	male	NA	Unmethylate	IDHwt	WT
TCGA-TM-A7CA-01A-21R-A33Z-07	TCGA-TM-A7CA-01A	astrocytoma	non-codel	PN	male	NA	Methylated	IDHmut-non-cod	Mutant
TCGA-HT-7620-01A-11R-2256-07	TCGA-HT-7620-01A	oligodendroglioma	codel	PN	male	TERT	Methylated	IDHmut-codel	Mutant
TCGA-HT-7471-01A-11R-2256-07	TCGA-HT-7471-01A	oligodendroglioma	codel	PN	female	TERT	Methylated	IDHmut-codel	Mutant
TCGA-FG-5964-01A-11R-1708-07	TCGA-FG-5964-01A	oligodendroglioma	codel	PN	male	TERT	Methylated	IDHmut-codel	Mutant
TCGA-FG-5965-02B-11R-A29R-07	TCGA-FG-5965-02B	NA	NA	NA	female	NA	NA	NA	NA
TCGA-DB-5270-01A-02R-1470-07	TCGA-DB-5270-01A	oligoastrocytoma	non-codel	NE	female	NA	Methylated	IDHmut-non-cod	Mutant
TCGA-P5-A5ET-01A-11R-A27Q-07	TCGA-P5-A5ET-01A	oligodendroglioma	codel	PN	male	TERT	Methylated	IDHmut-codel	Mutant
TCGA-CS-4944-01A-01R-1470-07	TCGA-CS-4944-01A	astrocytoma	non-codel	NA	male	TERT	Methylated	IDHmut-non-cod	Mutant
TCGA-R8-A6MO-01A-11R-A33Z-07	TCGA-R8-A6MO-01A	oligodendroglioma	codel	PN	female	NA	Methylated	IDHmut-codel	Mutant
TCGA-VM-A8C8-01A-11R-A36H-07	TCGA-VM-A8C8-01A	oligodendroglioma	non-codel	NA	female	NA	Unmethylate	IDHmut-non-cod	Mutant
TCGA-HT-A74L-01A-11R-A32Q-07	TCGA-HT-A74L-01A	NA	codel	PN	female	NA	Methylated	IDHmut-codel	Mutant
TCGA-FG-8187-01A-11R-2256-07	TCGA-FG-8187-01A	oligoastrocytoma	codel	NE	male	TERT	Methylated	IDHmut-codel	Mutant
TCGA-E1-A7YE-01A-11R-A34F-07	TCGA-E1-A7YE-01A	NA	non-codel	ME	female	NA	Unmethylate	IDHmut-non-cod	Mutant
TCGA-DU-5855-01A-11R-1708-07	TCGA-DU-5855-01A	oligoastrocytoma	non-codel	PN	female	ATRX	Methylated	IDHmut-non-cod	Mutant
TCGA-E1-5319-01A-01R-1896-07	TCGA-E1-5319-01A	oligodendroglioma	codel	PN	female	TERT	Methylated	IDHmut-codel	Mutant
TCGA-DU-A5TW-01A-11R-A28M-07	TCGA-DU-A5TW-01A	astrocytoma	non-codel	PN	female	ATRX	Methylated	IDHmut-non-cod	Mutant
TCGA-E1-A7YO-01A-11R-A34F-07	TCGA-E1-A7YO-01A	oligodendroglioma	codel	PN	male	NA	Methylated	IDHmut-codel	Mutant
TCGA-FG-7638-01B-12R-2090-07	TCGA-FG-7638-01B	oligodendroglioma	codel	NE	female	NA	Methylated	IDHmut-codel	Mutant
TCGA-HT-A5RA-01A-11R-A28M-07	TCGA-HT-A5RA-01A	astrocytoma	non-codel	CL	female	TERT	Unmethylate	IDHwt	WT
TCGA-DB-A4XD-01A-11R-A27Q-07	TCGA-DB-A4XD-01A	astrocytoma	non-codel	PN	male	ATRX	Methylated	IDHmut-non-cod	Mutant
TCGA-HT-8013-01A-11R-2403-07	TCGA-HT-8013-01A	oligoastrocytoma	non-codel	PN	female	ATRX	Methylated	IDHmut-non-cod	Mutant
TCGA-DU-6401-01A-11R-1708-07	TCGA-DU-6401-01A	oligodendroglioma	non-codel	PN	female	ATRX	Methylated	IDHmut-non-cod	Mutant
TCGA-DH-5141-01A-01R-1470-07	TCGA-DH-5141-01A	oligodendroglioma	codel	PN	male	TERT	Methylated	IDHmut-codel	Mutant
TCGA-HT-7477-01B-11R-A28M-07	TCGA-HT-7477-01B	astrocytoma	non-codel	PN	male	ATRX	Methylated	IDHmut-non-cod	Mutant
TCGA-HW-A5KJ-01A-12R-A27Q-07	TCGA-HW-A5KJ-01A	oligodendroglioma	codel	PN	male	TERT	Methylated	IDHmut-codel	Mutant
TCGA-P5-A780-01A-12R-A32Q-07	TCGA-P5-A780-01A	NA	non-codel	PN	female	NA	Methylated	IDHmut-non-cod	Mutant

TCGA-E1-A7YM-01A-11R-A34F-07	TCGA-E1-A7YM-01A	NA	non-codel	NE	male	NA	Unmethylate	IDHwt	WT
TCGA-DU-5870-01A-11R-1708-07	TCGA-DU-5870-01A	oligodendroglioma	codel	PN	female	TERT	Methylated	IDHmut-codel	Mutant
TCGA-FG-8188-01A-11R-2256-07	TCGA-FG-8188-01A	oligoastrocytoma	non-codel	NA	male	ATRX	Methylated	IDHmut-non-cod	Mutant
TCGA-S9-A6TV-01A-12R-A34R-07	TCGA-S9-A6TV-01A	oligoastrocytoma	non-codel	ME	male	NA	Methylated	IDHmut-non-cod	Mutant
TCGA-HT-A617-01A-11R-A29R-07	TCGA-HT-A617-01A	oligodendroglioma	non-codel	CL	male	TERT	Unmethylate	IDHwt	WT
TCGA-CS-4938-01B-11R-1896-07	TCGA-CS-4938-01B	astrocytoma	non-codel	NA	female	ATRX	Unmethylate	IDHmut-non-cod	Mutant
TCGA-E1-5311-01A-01R-1470-07	TCGA-E1-5311-01A	oligodendroglioma	codel	PN	male	TERT	Methylated	IDHmut-codel	Mutant
TCGA-DB-A4XH-01A-11R-A27Q-07	TCGA-DB-A4XH-01A	oligoastrocytoma	codel	NE	female	TERT	Methylated	IDHmut-codel	Mutant
TCGA-E1-A7YH-01A-11R-A34F-07	TCGA-E1-A7YH-01A	NA	non-codel	NA	female	NA	Methylated	IDHmut-non-cod	Mutant
TCGA-DH-A7UU-01A-12R-A34F-07	TCGA-DH-A7UU-01A	astrocytoma	non-codel	PN	male	NA	Methylated	IDHmut-non-cod	Mutant
TCGA-HW-7493-01A-11R-2027-07	TCGA-HW-7493-01A	astrocytoma	non-codel	NE	female	NA	Methylated	NA	NA
TCGA-P5-A5EY-01A-11R-A27Q-07	TCGA-P5-A5EY-01A	astrocytoma	non-codel	NE	male	-/-	Unmethylate	IDHwt	WT
TCGA-HT-8107-01A-13R-2404-07	TCGA-HT-8107-01A	oligodendroglioma	non-codel	NE	male	-/-	Methylated	IDHwt	WT
TCGA-FG-6692-01A-11R-1896-07	TCGA-FG-6692-01A	oligodendroglioma	non-codel	CL	male	TERT	Methylated	IDHwt	WT
TCGA-HW-7491-01A-11R-2027-07	TCGA-HW-7491-01A	oligodendroglioma	codel	PN	male	TERT	Unmethylate	IDHmut-codel	Mutant
TCGA-HW-7486-01A-11R-2027-07	TCGA-HW-7486-01A	oligodendroglioma	codel	PN	male	TERT	Methylated	IDHmut-codel	Mutant
TCGA-P5-A5EU-01A-11R-A27Q-07	TCGA-P5-A5EU-01A	astrocytoma	non-codel	NA	male	ATRX	Unmethylate	IDHmut-non-cod	Mutant
TCGA-DB-A4XF-01A-11R-A27Q-07	TCGA-DB-A4XF-01A	astrocytoma	non-codel	PN	female	-/-	Methylated	IDHmut-non-cod	Mutant
TCGA-HT-7691-01A-11R-2256-07	TCGA-HT-7691-01A	astrocytoma	non-codel	NA	female	-/-	Unmethylate	IDHwt	WT
TCGA-TM-A84O-01A-11R-A36H-07	TCGA-TM-A84O-01A	oligodendroglioma	codel	NA	female	NA	Methylated	IDHmut-codel	Mutant
TCGA-S9-A7R8-01A-11R-A34R-07	TCGA-S9-A7R8-01A	astrocytoma	non-codel	PN	female	NA	Methylated	IDHmut-non-cod	Mutant
TCGA-FG-A4MU-01B-11R-A28M-07	TCGA-FG-A4MU-01B	oligoastrocytoma	non-codel	CL	male	TERT	Methylated	IDHwt	WT
TCGA-CS-5393-01A-01R-1470-07	TCGA-CS-5393-01A	astrocytoma	non-codel	PN	male	-/-	Methylated	IDHmut-non-cod	Mutant
TCGA-VV-A829-01A-21R-A36H-07	TCGA-VV-A829-01A	oligoastrocytoma	codel	NA	male	NA	Methylated	IDHmut-codel	Mutant
TCGA-FG-8182-01A-11R-2256-07	TCGA-FG-8182-01A	oligodendroglioma	non-codel	PN	male	ATRX	Methylated	IDHmut-non-cod	Mutant
TCGA-HT-7881-01A-11R-2403-07	TCGA-HT-7881-01A	oligodendroglioma	codel	NE	male	TERT	Methylated	IDHmut-codel	Mutant
TCGA-DB-5277-01A-01R-1470-07	TCGA-DB-5277-01A	astrocytoma	non-codel	PN	male	-/-	Methylated	IDHmut-non-cod	Mutant
TCGA-S9-A7QZ-01A-12R-A34R-07	TCGA-S9-A7QZ-01A	oligodendroglioma	codel	PN	male	NA	Methylated	IDHmut-codel	Mutant
TCGA-DU-6542-01A-11R-1896-07	TCGA-DU-6542-01A	oligoastrocytoma	non-codel	PN	male	-/-	Methylated	IDHmut-non-cod	Mutant
TCGA-DU-6404-02B-11R-A36H-07	TCGA-DU-6404-02B	NA	NA	NA	female	NA	NA	NA	NA
TCGA-VW-A7QS-01A-12R-A33Z-07	TCGA-VW-A7QS-01A	oligodendroglioma	codel	NA	female	NA	Methylated	IDHmut-codel	Mutant
TCGA-DU-7018-01A-11R-2027-07	TCGA-DU-7018-01A	oligodendroglioma	codel	PN	female	TERT	Methylated	IDHmut-codel	Mutant
TCGA-QH-A65V-01A-11R-A29R-07	TCGA-QH-A65V-01A	oligodendroglioma	codel	NE	female	TERT	Methylated	IDHmut-codel	Mutant

TCGA-DU-A6S3-01A-12R-A32Q-07	TCGA-DU-A6S3-01A	oligodendroglioma	codel	PN	male	NA	Methylated	IDHmut-codel	Mutant
TCGA-DU-5872-01A-11R-1708-07	TCGA-DU-5872-01A	oligoastrocytoma	non-codel	PN	female	ATRX	Methylated	IDHmut-non-cod	Mutant
TCGA-S9-A7R3-01A-11R-A34R-07	TCGA-S9-A7R3-01A	astrocytoma	non-codel	NA	female	NA	Methylated	IDHmut-non-cod	Mutant
TCGA-S9-A6TW-01A-12R-A32Q-07	TCGA-S9-A6TW-01A	oligodendroglioma	codel	NA	male	NA	Methylated	IDHmut-codel	Mutant
TCGA-S9-A7QY-01A-11R-A34F-07	TCGA-S9-A7QY-01A	oligoastrocytoma	codel	PN	female	NA	Methylated	IDHmut-codel	Mutant
TCGA-DU-7008-01A-11R-2027-07	TCGA-DU-7008-01A	oligodendroglioma	non-codel	PN	female	ATRX	Methylated	IDHmut-non-cod	Mutant
TCGA-DH-5140-01A-01R-1470-07	TCGA-DH-5140-01A	oligoastrocytoma	non-codel	PN	female	-/-	Unmethylate	IDHwt	WT
TCGA-HT-7601-01A-11R-2090-07	TCGA-HT-7601-01A	astrocytoma	non-codel	NA	female	-/-	Methylated	IDHmut-non-cod	Mutant
TCGA-DU-7298-01A-11R-2027-07	TCGA-DU-7298-01A	astrocytoma	non-codel	PN	female	ATRX	Methylated	IDHmut-non-cod	Mutant
TCGA-S9-A7QX-01A-11R-A34F-07	TCGA-S9-A7QX-01A	astrocytoma	non-codel	PN	female	NA	Methylated	IDHmut-non-cod	Mutant
TCGA-HT-A4DV-01A-11R-A26U-07	TCGA-HT-A4DV-01A	oligodendroglioma	codel	NE	female	TERT	Methylated	IDHmut-codel	Mutant
TCGA-FG-A60J-01A-11R-A28M-07	TCGA-FG-A60J-01A	oligoastrocytoma	non-codel	PN	female	TERT	Methylated	IDHmut-non-cod	Mutant
TCGA-HT-7693-01A-11R-2256-07	TCGA-HT-7693-01A	oligodendroglioma	non-codel	NA	female	ATRX	Methylated	IDHmut-non-cod	Mutant
TCGA-DU-6407-02B-11R-A36H-07	TCGA-DU-6407-02B	NA	NA	NA	female	NA	NA	NA	NA
TCGA-S9-A7R2-01A-21R-A34R-07	TCGA-S9-A7R2-01A	astrocytoma	non-codel	NA	male	NA	Unmethylate	IDHwt	WT
TCGA-DU-8162-01A-21R-2256-07	TCGA-DU-8162-01A	oligoastrocytoma	non-codel	NE	female	-/-	Unmethylate	IDHwt	WT
TCGA-HT-A5RC-01A-11R-A28M-07	TCGA-HT-A5RC-01A	astrocytoma	non-codel	CL	female	-/-	Unmethylate	IDHwt	WT
TCGA-FG-A710-01A-12R-A33Z-07	TCGA-FG-A710-01A	oligodendroglioma	codel	PN	female	NA	Methylated	IDHmut-codel	Mutant
TCGA-HT-7467-01A-11R-2027-07	TCGA-HT-7467-01A	oligodendroglioma	codel	NE	male	TERT	Methylated	IDHmut-codel	Mutant
TCGA-HT-7605-01A-11R-2090-07	TCGA-HT-7605-01A	oligodendroglioma	codel	NE	male	TERT	Methylated	IDHmut-codel	Mutant
TCGA-DB-A75P-01A-11R-A32Q-07	TCGA-DB-A75P-01A	astrocytoma	non-codel	NE	female	NA	Unmethylate	IDHwt	WT
TCGA-TQ-A8XE-01A-11R-A36H-07	TCGA-TQ-A8XE-01A	NA	non-codel	NA	female	-/-	Methylated	IDHmut-non-cod	Mutant
TCGA-DU-7299-01A-21R-2027-07	TCGA-DU-7299-01A	astrocytoma	non-codel	PN	male	-/-	Methylated	IDHmut-non-cod	Mutant
TCGA-DU-7006-01A-11R-2027-07	TCGA-DU-7006-01A	astrocytoma	non-codel	ME	female	TERT	Methylated	IDHwt	WT
TCGA-HT-A4DS-01A-11R-A26U-07	TCGA-HT-A4DS-01A	astrocytoma	non-codel	CL	female	TERT	Unmethylate	IDHwt	WT
TCGA-DU-6405-01A-11R-1708-07	TCGA-DU-6405-01A	astrocytoma	non-codel	CL	female	TERT	Methylated	IDHwt	WT
TCGA-S9-A7J0-01A-11R-A34F-07	TCGA-S9-A7J0-01A	oligodendroglioma	non-codel	PN	female	NA	Methylated	IDHmut-non-cod	Mutant
TCGA-CS-6669-01A-11R-1896-07	TCGA-CS-6669-01A	oligodendroglioma	non-codel	NE	female	-/-	Unmethylate	IDHwt	WT
TCGA-TQ-A7RQ-01A-11R-A33Z-07	TCGA-TQ-A7RQ-01A	NA	codel	PN	female	NA	Methylated	IDHmut-codel	Mutant
TCGA-EZ-7264-01A-11R-2027-07	TCGA-EZ-7264-01A	oligodendroglioma	codel	PN	female	TERT	Methylated	IDHmut-codel	Mutant
TCGA-CS-5397-01A-01R-1896-07	TCGA-CS-5397-01A	astrocytoma	non-codel	NE	female	TERT	Unmethylate	IDHwt	WT
TCGA-DU-8163-01A-11R-2256-07	TCGA-DU-8163-01A	oligoastrocytoma	non-codel	PN	male	ATRX	Unmethylate	IDHmut-non-cod	Mutant
TCGA-TQ-A7RK-01A-11R-A33Z-07	TCGA-TQ-A7RK-01A	NA	non-codel	PN	male	ATRX	Methylated	IDHmut-non-cod	Mutant

TCGA-DU-A76R-01A-11R-A32Q-07	TCGA-DU-A76R-01A	oligodendroglioma	codel	NE	male	NA	Methylated	IDHmut-codel	Mutant
TCGA-QH-A65Z-01A-11R-A29R-07	TCGA-QH-A65Z-01A	oligodendroglioma	codel	PN	male	TERT	Methylated	IDHmut-codel	Mutant
TCGA-DU-6396-01A-11R-1708-07	TCGA-DU-6396-01A	oligoastrocytoma	non-codel	PN	female	ATRX	Methylated	IDHmut-non-cod	Mutant
TCGA-RY-A840-01A-11R-A36H-07	TCGA-RY-A840-01A	NA	codel	NA	male	NA	Methylated	IDHmut-codel	Mutant
TCGA-P5-A5F4-01A-11R-A28M-07	TCGA-P5-A5F4-01A	oligodendroglioma	non-codel	NE	female	-/-	Methylated	IDHmut-non-cod	Mutant
TCGA-DB-5281-01A-01R-1470-07	TCGA-DB-5281-01A	oligoastrocytoma	non-codel	NE	male	ATRX	Methylated	IDHmut-non-cod	Mutant
TCGA-DB-5275-01A-01R-1470-07	TCGA-DB-5275-01A	oligoastrocytoma	non-codel	PN	male	ATRX	Methylated	IDHmut-non-cod	Mutant
TCGA-IK-8125-01A-11R-2256-07	TCGA-IK-8125-01A	oligoastrocytoma	codel	NE	male	TERT	Methylated	IDHmut-codel	Mutant
TCGA-HT-A615-01A-11R-A29R-07	TCGA-HT-A615-01A	oligodendroglioma	codel	PN	female	TERT	Methylated	IDHmut-codel	Mutant
TCGA-FG-8181-01A-11R-2256-07	TCGA-FG-8181-01A	oligoastrocytoma	non-codel	NE	male	-/-	Unmethylate	IDHwt	WT
TCGA-HT-8114-01A-11R-2404-07	TCGA-HT-8114-01A	oligoastrocytoma	non-codel	NA	male	ATRX	Methylated	IDHmut-non-cod	Mutant
TCGA-DB-A64O-01A-11R-A29R-07	TCGA-DB-A64O-01A	oligoastrocytoma	non-codel	NE	male	TERT	Unmethylate	IDHwt	WT
TCGA-DU-5849-01A-11R-1708-07	TCGA-DU-5849-01A	oligodendroglioma	codel	NE	male	TERT	Methylated	IDHmut-codel	Mutant
TCGA-DH-5144-01A-01R-1470-07	TCGA-DH-5144-01A	oligodendroglioma	codel	PN	female	NA	Methylated	IDHmut-codel	Mutant
TCGA-FG-8189-01B-11R-A28M-07	TCGA-FG-8189-01B	oligodendroglioma	non-codel	NE	female	-/-	Methylated	IDHmut-non-cod	Mutant
TCGA-S9-A6WH-01A-12R-A33Z-07	TCGA-S9-A6WH-01A	oligoastrocytoma	codel	PN	female	NA	Methylated	IDHmut-codel	Mutant
TCGA-WY-A85B-01A-11R-A36H-07	TCGA-WY-A85B-01A	NA	non-codel	NA	male	NA	Methylated	IDHmut-non-cod	Mutant
TCGA-TM-A84R-01A-21R-A36H-07	TCGA-TM-A84R-01A	oligodendroglioma	codel	NA	male	NA	Methylated	IDHmut-codel	Mutant
TCGA-DU-A76L-01A-11R-A32Q-07	TCGA-DU-A76L-01A	oligodendroglioma	non-codel	ME	male	NA	Methylated	IDHwt	WT
TCGA-CS-4942-01A-01R-1470-07	TCGA-CS-4942-01A	astrocytoma	non-codel	PN	female	ATRX	Unmethylate	IDHmut-non-cod	Mutant
TCGA-S9-A6TY-01A-12R-A32Q-07	TCGA-S9-A6TY-01A	oligodendroglioma	codel	PN	male	NA	Methylated	IDHmut-codel	Mutant
TCGA-QH-A86X-01A-11R-A36H-07	TCGA-QH-A86X-01A	oligodendroglioma	codel	NA	male	NA	Methylated	IDHmut-codel	Mutant
TCGA-DU-7290-01A-11R-2027-07	TCGA-DU-7290-01A	astrocytoma	non-codel	ME	female	TERT	Unmethylate	IDHwt	WT
TCGA-QH-A6XC-01A-12R-A32Q-07	TCGA-QH-A6XC-01A	astrocytoma	non-codel	CL	male	NA	Methylated	IDHwt	WT
TCGA-TM-A84T-01A-11R-A36H-07	TCGA-TM-A84T-01A	oligoastrocytoma	non-codel	NA	male	NA	Methylated	IDHmut-non-cod	Mutant
TCGA-HT-8010-01A-11R-2403-07	TCGA-HT-8010-01A	oligodendroglioma	codel	NE	female	-/-	Methylated	IDHmut-codel	Mutant
TCGA-DU-A5TS-01A-11R-A28M-07	TCGA-DU-A5TS-01A	oligodendroglioma	non-codel	NA	male	ATRX	Methylated	IDHmut-non-cod	Mutant
TCGA-DU-7010-01A-11R-2027-07	TCGA-DU-7010-01A	astrocytoma	non-codel	PN	female	-/-	Methylated	IDHmut-non-cod	Mutant
TCGA-DU-A6S7-01A-21R-A32Q-07	TCGA-DU-A6S7-01A	astrocytoma	non-codel	PN	female	NA	Methylated	IDHmut-non-cod	Mutant
TCGA-HT-8109-01A-11R-2404-07	TCGA-HT-8109-01A	oligodendroglioma	codel	NE	male	TERT	Methylated	IDHmut-codel	Mutant
TCGA-FG-A4MY-01A-11R-A26U-07	TCGA-FG-A4MY-01A	oligoastrocytoma	non-codel	NA	female	ATRX	Methylated	IDHmut-non-cod	Mutant
TCGA-HT-A61B-01A-11R-A29R-07	TCGA-HT-A61B-01A	NA	non-codel	NA	male	-/-	Methylated	IDHmut-non-cod	Mutant
TCGA-QH-A65X-01A-11R-A32Q-07	TCGA-QH-A65X-01A	oligoastrocytoma	codel	NA	female	NA	Methylated	IDHmut-codel	Mutant

TCGA-HT-8019-01A-21R-2404-07	TCGA-HT-8019-01A	oligodendroglioma	non-codel	NE	female	-/-	Unmethylate	IDHwt	WT
TCGA-DU-5854-01A-11R-1708-07	TCGA-DU-5854-01A	astrocytoma	non-codel	CL	female	TERT	Unmethylate	IDHwt	WT
TCGA-DU-6408-01A-11R-1708-07	TCGA-DU-6408-01A	oligodendroglioma	non-codel	PN	female	ATRX	Methylated	IDHmut-non-cod	Mutant
TCGA-S9-A7IZ-01A-11R-A34F-07	TCGA-S9-A7IZ-01A	astrocytoma	non-codel	NE	female	NA	Methylated	IDHmut-non-cod	Mutant
TCGA-P5-A5EX-01A-12R-A28M-07	TCGA-P5-A5EX-01A	oligodendroglioma	codel	ME	female	TERT	Methylated	IDHmut-codel	Mutant
TCGA-E1-A7YL-01A-11R-A34F-07	TCGA-E1-A7YL-01A	NA	non-codel	ME	male	NA	Unmethylate	IDHwt	WT
TCGA-DU-8164-01A-11R-2256-07	TCGA-DU-8164-01A	oligodendroglioma	codel	PN	male	TERT	Methylated	IDHmut-codel	Mutant
TCGA-FG-A711-01A-21R-A33Z-07	TCGA-FG-A711-01A	oligodendroglioma	non-codel	NE	female	NA	Unmethylate	IDHmut-non-cod	Mutant
TCGA-TQ-A7RV-02A-11R-A36H-07	TCGA-TQ-A7RV-02A	NA	NA	NA	male	NA	NA	NA	NA
TCGA-HW-7495-01A-11R-2027-07	TCGA-HW-7495-01A	oligodendroglioma	codel	NE	female	TERT	Methylated	IDHmut-codel	Mutant
TCGA-HT-7860-01A-11R-2403-07	TCGA-HT-7860-01A	astrocytoma	non-codel	CL	female	TERT	Methylated	IDHwt	WT
TCGA-TM-A84C-01A-11R-A36H-07	TCGA-TM-A84C-01A	astrocytoma	non-codel	NA	male	NA	Unmethylate	IDHwt	WT
TCGA-DU-A76K-01A-11R-A33Z-07	TCGA-DU-A76K-01A	oligodendroglioma	non-codel	NE	male	NA	Unmethylate	IDHwt	WT
TCGA-FG-5965-01B-11R-1896-07	TCGA-FG-5965-01B	oligoastrocytoma	non-codel	PN	female	ATRX	Methylated	IDHmut-non-cod	Mutant
TCGA-S9-A6U8-01A-21R-A33Z-07	TCGA-S9-A6U8-01A	astrocytoma	non-codel	PN	male	NA	Methylated	IDHmut-non-cod	Mutant
TCGA-S9-A7IQ-01A-21R-A34F-07	TCGA-S9-A7IQ-01A	oligoastrocytoma	codel	NE	female	NA	Methylated	IDHmut-codel	Mutant
TCGA-S9-A6TZ-01A-21R-A32Q-07	TCGA-S9-A6TZ-01A	astrocytoma	non-codel	PN	female	NA	Methylated	IDHmut-non-cod	Mutant
TCGA-DU-7300-01A-21R-2090-07	TCGA-DU-7300-01A	oligodendroglioma	codel	NE	female	TERT	Methylated	IDHmut-codel	Mutant
TCGA-E1-A7YJ-01A-11R-A34F-07	TCGA-E1-A7YJ-01A	NA	non-codel	CL	male	NA	Unmethylate	IDHwt	WT
TCGA-DU-A6S2-01A-21R-A32Q-07	TCGA-DU-A6S2-01A	oligodendroglioma	codel	NE	female	NA	Methylated	IDHmut-codel	Mutant
TCGA-HT-A619-01A-11R-A29R-07	TCGA-HT-A619-01A	oligodendroglioma	codel	PN	female	TERT	Methylated	IDHmut-codel	Mutant
TCGA-RY-A847-01A-11R-A36H-07	TCGA-RY-A847-01A	NA	codel	NA	male	NA	Methylated	IDHmut-codel	Mutant
TCGA-S9-A6U5-01A-12R-A33Z-07	TCGA-S9-A6U5-01A	astrocytoma	codel	PN	male	NA	Methylated	IDHmut-codel	Mutant
TCGA-HT-8011-01A-11R-2403-07	TCGA-HT-8011-01A	astrocytoma	non-codel	NA	male	TERT	Unmethylate	IDHwt	WT
TCGA-DB-A75O-01A-11R-A32Q-07	TCGA-DB-A75O-01A	astrocytoma	non-codel	NA	male	NA	Methylated	IDHmut-non-cod	Mutant
TCGA-DB-A64U-01A-11R-A29R-07	TCGA-DB-A64U-01A	oligoastrocytoma	codel	PN	female	TERT	Methylated	IDHmut-codel	Mutant
TCGA-WY-A858-01A-11R-A36H-07	TCGA-WY-A858-01A	astrocytoma	non-codel	NA	female	NA	Methylated	IDHmut-non-cod	Mutant
TCGA-P5-A5EZ-01A-11R-A27Q-07	TCGA-P5-A5EZ-01A	astrocytoma	non-codel	PN	male	-/-	Methylated	IDHmut-non-cod	Mutant
TCGA-E1-A7YK-01A-11R-A34F-07	TCGA-E1-A7YK-01A	NA	non-codel	ME	male	NA	Methylated	IDHmut-non-cod	Mutant
TCGA-CS-5390-01A-02R-1470-07	TCGA-CS-5390-01A	oligodendroglioma	codel	PN	female	TERT	Methylated	IDHmut-codel	Mutant
TCGA-HT-7475-01A-11R-2027-07	TCGA-HT-7475-01A	oligoastrocytoma	non-codel	NE	male	ATRX	Methylated	IDHmut-non-cod	Mutant
TCGA-DU-7302-01A-11R-2090-07	TCGA-DU-7302-01A	oligodendroglioma	codel	NE	female	TERT	Methylated	IDHmut-codel	Mutant
TCGA-S9-A6TX-01A-21R-A32Q-07	TCGA-S9-A6TX-01A	oligodendroglioma	codel	PN	male	NA	Methylated	IDHmut-codel	Mutant

TCGA-S9-A7J1-01A-21R-A34R-07	TCGA-S9-A7J1-01A	oligodendroglioma	codel	PN	male	NA	Methylated	IDHmut-codel	Mutant
TCGA-WH-A86K-01A-11R-A36H-07	TCGA-WH-A86K-01A	astrocytoma	non-codel	NA	male	NA	Methylated	IDHmut-non-cod	Mutant
TCGA-FG-A60K-01A-11R-A29R-07	TCGA-FG-A60K-01A	oligoastrocytoma	codel	PN	female	TERT	Methylated	IDHmut-codel	Mutant
TCGA-CS-6290-01A-11R-1708-07	TCGA-CS-6290-01A	astrocytoma	non-codel	NA	male	-/-	Methylated	IDHmut-non-cod	Mutant
TCGA-HW-8319-01A-11R-2404-07	TCGA-HW-8319-01A	astrocytoma	non-codel	PN	female	ATRX	Unmethylate	IDHmut-non-cod	Mutant
TCGA-HT-7688-01A-11R-2256-07	TCGA-HT-7688-01A	oligodendroglioma	non-codel	NE	male	ATRX	Methylated	IDHmut-non-cod	Mutant
TCGA-HT-7479-01A-11R-2027-07	TCGA-HT-7479-01A	astrocytoma	non-codel	NA	male	TERT	Methylated	IDHmut-non-cod	Mutant
TCGA-DB-5278-01A-01R-1470-07	TCGA-DB-5278-01A	oligodendroglioma	codel	PN	male	-/-	Methylated	IDHmut-codel	Mutant
TCGA-FG-7634-01A-11R-2090-07	TCGA-FG-7634-01A	oligodendroglioma	codel	PN	male	TERT	Methylated	IDHmut-codel	Mutant
TCGA-S9-A7J3-01A-21R-A34R-07	TCGA-S9-A7J3-01A	oligodendroglioma	codel	PN	female	NA	Methylated	IDHmut-codel	Mutant
TCGA-TQ-A7RS-01A-12R-A33Z-07	TCGA-TQ-A7RS-01A	NA	codel	PN	female	NA	Methylated	IDHmut-codel	Mutant
TCGA-TM-A7CF-01A-11R-A32Q-07	TCGA-TM-A7CF-01A	astrocytoma	non-codel	NE	female	-/-	Methylated	IDHmut-non-cod	Mutant
TCGA-S9-A6WN-01A-12R-A33Z-07	TCGA-S9-A6WN-01A	astrocytoma	codel	ME	female	NA	Methylated	IDHmut-codel	Mutant
TCGA-DU-8165-01A-11R-2256-07	TCGA-DU-8165-01A	oligodendroglioma	non-codel	NA	female	TERT	Unmethylate	IDHwt	WT
TCGA-S9-A6U0-01A-12R-A32Q-07	TCGA-S9-A6U0-01A	astrocytoma	non-codel	ME	male	NA	Methylated	IDHwt	WT
TCGA-P5-A5EV-01A-11R-A27Q-07	TCGA-P5-A5EV-01A	astrocytoma	non-codel	PN	male	ATRX	Methylated	IDHmut-non-cod	Mutant
TCGA-HW-8322-01A-11R-2404-07	TCGA-HW-8322-01A	oligodendroglioma	codel	PN	male	TERT	Methylated	IDHmut-codel	Mutant
TCGA-QH-A65S-01A-11R-A29R-07	TCGA-QH-A65S-01A	oligoastrocytoma	non-codel	PN	female	ATRX	Methylated	IDHmut-non-cod	Mutant
TCGA-E1-A7Z4-01A-11R-A34R-07	TCGA-E1-A7Z4-01A	NA	non-codel	NA	male	NA	Methylated	IDHmut-non-cod	Mutant
TCGA-TQ-A8XE-02A-11R-A36H-07	TCGA-TQ-A8XE-02A	NA	NA	NA	female	NA	NA	NA	NA
TCGA-HT-A61C-01A-11R-A29R-07	TCGA-HT-A61C-01A	oligodendroglioma	non-codel	NA	male	TERT	Unmethylate	IDHwt	WT
TCGA-HW-7487-01A-11R-2027-07	TCGA-HW-7487-01A	oligodendroglioma	codel	NE	male	TERT	Methylated	IDHmut-codel	Mutant
TCGA-QH-A870-01A-11R-A36H-07	TCGA-QH-A870-01A	oligoastrocytoma	non-codel	NA	female	NA	Methylated	IDHmut-non-cod	Mutant
TCGA-CS-5394-01A-01R-1470-07	TCGA-CS-5394-01A	astrocytoma	non-codel	PN	male	-/-	Methylated	IDHmut-non-cod	Mutant
TCGA-DU-A7T8-01A-21R-A34R-07	TCGA-DU-A7T8-01A	oligoastrocytoma	non-codel	PN	male	NA	Methylated	IDHmut-non-cod	Mutant
TCGA-TM-A84J-01A-11R-A36H-07	TCGA-TM-A84J-01A	oligodendroglioma	non-codel	NA	male	NA	Unmethylate	IDHwt	WT
TCGA-HT-7603-01A-21R-2090-07	TCGA-HT-7603-01A	oligodendroglioma	non-codel	NE	male	ATRX	Methylated	IDHmut-non-cod	Mutant
TCGA-E1-5305-01A-01R-1896-07	TCGA-E1-5305-01A	astrocytoma	non-codel	NE	male	ATRX	Methylated	IDHmut-non-cod	Mutant
TCGA-CS-5395-01A-01R-1470-07	TCGA-CS-5395-01A	oligodendroglioma	non-codel	CL	male	TERT	Unmethylate	IDHwt	WT
TCGA-TQ-A7RK-02B-11R-A40A-07	TCGA-TQ-A7RK-02B	NA	NA	NA	male	NA	NA	NA	NA
TCGA-DU-8158-01A-11R-2256-07	TCGA-DU-8158-01A	astrocytoma	non-codel	NA	female	TERT	Unmethylate	IDHwt	WT
TCGA-E1-A7YN-01A-11R-A34F-07	TCGA-E1-A7YN-01A	NA	non-codel	ME	female	NA	Methylated	IDHwt	WT
TCGA-S9-A7R4-01A-12R-A34R-07	TCGA-S9-A7R4-01A	astrocytoma	non-codel	PN	male	NA	Methylated	IDHmut-non-cod	Mutant

TCGA-W9-A837-01A-11R-A36H-07	TCGA-W9-A837-01A	oligodendroglioma	codel	NA	male	NA	Methylated	IDHmut-codel	Mutant
TCGA-P5-A77X-01A-11R-A32Q-07	TCGA-P5-A77X-01A	NA	codel	NE	female	NA	Methylated	IDHmut-codel	Mutant
TCGA-FG-6689-01A-11R-1896-07	TCGA-FG-6689-01A	astrocytoma	non-codel	NE	male	NA	Methylated	IDHmut-non-cod	Mutant
TCGA-HT-7469-01A-11R-2256-07	TCGA-HT-7469-01A	oligodendroglioma	non-codel	CL	male	ATRX	Methylated	IDHwt	WT
TCGA-DU-6404-02A-21R-A36H-07	TCGA-DU-6404-02A	NA	NA	NA	female	NA	NA	NA	NA
TCGA-HT-7687-01A-11R-2256-07	TCGA-HT-7687-01A	oligodendroglioma	codel	PN	male	TERT	Methylated	IDHmut-codel	Mutant
TCGA-DU-7009-01A-11R-2027-07	TCGA-DU-7009-01A	oligodendroglioma	codel	PN	female	TERT	Methylated	IDHmut-codel	Mutant
TCGA-HT-7677-01A-11R-2256-07	TCGA-HT-7677-01A	oligodendroglioma	codel	PN	male	TERT	Methylated	IDHmut-codel	Mutant
TCGA-HT-7684-01A-11R-2256-07	TCGA-HT-7684-01A	oligoastrocytoma	non-codel	NA	male	TERT	Methylated	IDHmut-non-cod	Mutant
TCGA-DU-A6S6-01A-21R-A32Q-07	TCGA-DU-A6S6-01A	oligoastrocytoma	codel	NE	female	NA	Methylated	IDHmut-codel	Mutant
TCGA-R8-A6YH-01A-21R-A32Q-07	TCGA-R8-A6YH-01A	NA	non-codel	PN	NA	NA	Unmethylate	IDHmut-non-cod	Mutant
TCGA-HT-8111-01A-11R-2404-07	TCGA-HT-8111-01A	oligoastrocytoma	non-codel	PN	male	-/-	Methylated	IDHmut-non-cod	Mutant
TCGA-HT-7681-01A-11R-2403-07	TCGA-HT-7681-01A	oligoastrocytoma	codel	NE	female	TERT	Methylated	IDHmut-codel	Mutant
TCGA-HT-A616-01A-11R-A29R-07	TCGA-HT-A616-01A	astrocytoma	non-codel	NE	female	ATRX	Methylated	IDHmut-non-cod	Mutant
TCGA-DU-7019-01A-11R-2027-07	TCGA-DU-7019-01A	oligoastrocytoma	non-codel	NA	male	-/-	Methylated	IDHmut-non-cod	Mutant
TCGA-HT-8113-01A-11R-2404-07	TCGA-HT-8113-01A	oligodendroglioma	non-codel	NE	female	TERT	Methylated	IDHmut-non-cod	Mutant
TCGA-HT-7873-01B-11R-2403-07	TCGA-HT-7873-01B	oligoastrocytoma	non-codel	PN	male	ATRX	Methylated	IDHmut-non-cod	Mutant
TCGA-HW-8320-01A-11R-2404-07	TCGA-HW-8320-01A	astrocytoma	non-codel	PN	male	-/-	Methylated	IDHmut-non-cod	Mutant
TCGA-HT-A74J-01A-12R-A32Q-07	TCGA-HT-A74J-01A	oligoastrocytoma	non-codel	PN	male	NA	Unmethylate	IDHmut-non-cod	Mutant
TCGA-DU-5872-02A-21R-A36H-07	TCGA-DU-5872-02A	NA	NA	NA	female	NA	NA	NA	NA
TCGA-RY-A845-01A-11R-A36H-07	TCGA-RY-A845-01A	NA	non-codel	NA	female	NA	Methylated	IDHmut-non-cod	Mutant
TCGA-E1-A7YW-01A-11R-A34R-07	TCGA-E1-A7YW-01A	oligoastrocytoma	non-codel	PN	male	NA	Methylated	IDHmut-non-cod	Mutant
TCGA-DU-A76O-01A-11R-A32Q-07	TCGA-DU-A76O-01A	astrocytoma	non-codel	PN	male	NA	Methylated	IDHmut-non-cod	Mutant
TCGA-S9-A6UA-01A-12R-A33Z-07	TCGA-S9-A6UA-01A	astrocytoma	non-codel	ME	male	NA	Methylated	IDHwt	WT
TCGA-HT-A74H-01A-11R-A32Q-07	TCGA-HT-A74H-01A	astrocytoma	non-codel	NE	male	NA	Unmethylate	IDHwt	WT
TCGA-S9-A6WE-01A-12R-A33Z-07	TCGA-S9-A6WE-01A	oligodendroglioma	codel	PN	male	NA	Methylated	IDHmut-codel	Mutant
TCGA-S9-A6WO-01A-21R-A34F-07	TCGA-S9-A6WO-01A	astrocytoma	non-codel	PN	male	NA	Methylated	IDHmut-non-cod	Mutant
TCGA-DU-7306-01A-11R-2090-07	TCGA-DU-7306-01A	oligoastrocytoma	non-codel	NA	male	ATRX	Methylated	IDHmut-non-cod	Mutant
TCGA-HW-A5KK-01A-11R-A27Q-07	TCGA-HW-A5KK-01A	astrocytoma	non-codel	CL	male	TERT	Methylated	IDHwt	WT
TCGA-HT-A5RB-01A-11R-A28M-07	TCGA-HT-A5RB-01A	astrocytoma	non-codel	PN	male	ATRX	Methylated	IDHmut-non-cod	Mutant
TCGA-WY-A85C-01A-11R-A36H-07	TCGA-WY-A85C-01A	NA	non-codel	NA	male	NA	Methylated	IDHmut-non-cod	Mutant
TCGA-HT-7676-01A-11R-2403-07	TCGA-HT-7676-01A	oligodendroglioma	non-codel	PN	male	ATRX	Unmethylate	IDHmut-non-cod	Mutant
TCGA-CS-4941-01A-01R-1470-07	TCGA-CS-4941-01A	astrocytoma	non-codel	CL	male	TERT	Methylated	IDHwt	WT

TCGA-E1-A7YV-01A-11R-A34R-07	TCGA-E1-A7YV-01A	oligoastrocytoma	non-codel	PN	female	NA	Methylated	IDHmut-non-cod	Mutant
TCGA-HT-7884-01B-11R-2403-07	TCGA-HT-7884-01B	astrocytoma	non-codel	PN	female	ATRX	Methylated	IDHmut-non-cod	Mutant
TCGA-VM-A8CH-01A-12R-A36H-07	TCGA-VM-A8CH-01A	astrocytoma	non-codel	NA	female	NA	Unmethylate	IDHmut-non-cod	Mutant
TCGA-P5-A72Z-01A-11R-A32Q-07	TCGA-P5-A72Z-01A	oligodendroglioma	codel	PN	female	NA	Methylated	IDHmut-codel	Mutant
TCGA-FG-8191-01A-11R-2256-07	TCGA-FG-8191-01A	oligodendroglioma	non-codel	PN	male	ATRX	Unmethylate	IDHmut-non-cod	Mutant
TCGA-FG-7641-01B-11R-2256-07	TCGA-FG-7641-01B	oligodendroglioma	codel	NE	male	TERT	Methylated	IDHmut-codel	Mutant
TCGA-S9-A6WI-01A-21R-A33Z-07	TCGA-S9-A6WI-01A	oligoastrocytoma	non-codel	NE	female	NA	Methylated	IDHmut-non-cod	Mutant
TCGA-06-0675-11A	TCGA-06-0675-11A	non-tumor							
TCGA-06-0678-11A	TCGA-06-0678-11A	non-tumor							
TCGA-06-0680-11A	TCGA-06-0680-11A	non-tumor							
TCGA-06-0681-11A	TCGA-06-0681-11A	non-tumor							
TCGA-06-AABW-11A	TCGA-06-AABW-11A	non-tumor							
