# Supplementary material for: The tRNA-Cys-GCA Derived tsRNAs Suppress Tumor Progression of Gliomas via Regulating VAV2
Source: Dis Markers. 2022 Nov 15;2022:8708312. doi: 10.1155/2022/8708312 (PMC9681550; doi:10.1155/2022/8708312)
Supplement: Supplementary Materials — Figure S1: a summary of tsRNAs identification and characterization. (A) the flow chart of data processing and tsRNAs identification pipeline. (C) The characterization of tsRNA corresponding derived tRNA gene sources and (B) the chromosome locations statistical analysis. Figure S2: the enrichment analyses of tRFdb-3003b -related genes within TCGA-LGG datasets. (A) The top gene ontology (GO) terms, including biological process, CC (cellular component) and MF (molecular function), as well as the top KEGG pathway for tRFdb-3003b-related genes. (B) The correlation scatter-plots of tRFdb-3003b and its correlated-genes (ATG4B, LUC7L, D2HGDH, and HDAC10). (C-left) GSEA (gene set enrichment analysis) plots of three molecular signatures (chr6p21, microglia, and STIM treatment response signature), (C-right) the scatter plots for tRFdb-3003b and its correlated-genes (CRIP3 and ANKRD13B). Table S1: the primary clinical and molecular pathology characteristics parameters in glioma samples. Table S2: the primers used in quantitative real-time PCR assay. Table S3: a summary of the identified tsRNAs with available expression abundance in glioma datasets. Table S4: the expression profiles of tRNA-Cys-GCA derived tsRNAs with in glioma samples. [file 8708312.f1.zip › Supplemental Table S3.pdf]

Table S3. A summary of the identified tsRNAs with available expression abundance in glioma datasets.

tsRNA	types	median	mean	min	max
tRFdb-3003a	3‘-tRF	14.289	13.877	7.7285	18.602
tRFdb-3003b	3‘-tRF	13.917	13.506	7.3585	18.23
tRFdb-3020a	3‘-tRF	14.574	14.707	9.9196	18.193
tRFdb-3012a	3‘-tRF	15.872	15.318	7.8317	17.984
tRFdb-3012b	3‘-tRF	15.632	15.134	8.2924	17.694
tRFdb-3029a	3‘-tRF	13.76	13.731	8.207	17.833
tRFdb-3029b	3‘-tRF	12.435	12.383	7.2217	17.34
tRFdb-5005b	5‘-tRF	12.039	11.909	7.0386	17.829
tRFdb-5005c	5‘-tRF	11.729	11.598	6.7309	17.519
tRFdb-5026a	5‘-tRF	12.039	11.976	7.2074	18.27
5P_tRNA-Lys-TTT-3-1	5‘U-tRF	12.157	12.06	7.3584	18.142
tRFdb-3030a	3‘-tRF	17.162	17.173	9.1163	18.878
tRFdb-3030b	3‘-tRF	16.872	16.884	8.8274	18.589
tRFdb-3033a	3‘-tRF	12.217	12.074	7.5631	18.41
tRFdb-3006a	3‘-tRF	12.478	12.355	7.0734	17.775
tRFdb-3006b	3‘-tRF	12.106	11.983	6.7046	17.403
tRFdb-3016a	3‘-tRF	11.708	11.654	6.8395	16.209
tRFdb-3016b	3‘-tRF	11.497	11.442	6.63	15.997
ts-96	tRF-1	13.33	13.175	5.5544	17.608
tRFdb-5002a	5‘-tRF	15.189	15.176	8.5685	18.608
tRFdb-5002b	5‘-tRF	14.749	14.735	8.1293	18.167
tRFdb-5002c	5‘-tRF	14.043	14.029	7.4263	17.461
tRFdb-3001a	3‘-tRF	11.955	11.89	7.2823	18.193
tRFdb-3013a	3‘-tRF	11.417	11.343	6.662	16.41
tRFdb-3013b	3‘-tRF	11.045	10.971	6.2942	16.039
tRFdb-3021a	3‘-tRF	11.84	11.739	6.1712	15.3
ts-26	tRF-1	15.246	14.389	5.3882	17.346
tRFdb-3015a	3‘-tRF	13.386	13.202	6.7256	16.171
tRFdb-3015b	3‘-tRF	13.174	12.99	6.5163	15.959
tRFdb-5027b	5‘-tRF	10.364	10.27	6.2337	15.968
tRFdb-5027c	5‘-tRF	9.9282	9.8448	5.8097	15.537
tRFdb-5017a	5‘-tRF	12.049	11.929	6.8707	17.183
tRFdb-5017b	5‘-tRF	11.165	11.048	5.9966	16.298
tRFdb-3024a	3‘-tRF	11.316	11.384	6.5174	15.44
tRFdb-3025a	3‘-tRF	11.316	11.384	6.5174	15.44
ts-58	tRF-1	10.5	10.427	4.4122	14.612
tRFdb-3024b	3‘-tRF	10.812	10.846	6.2314	15.119
tRFdb-3025b	3‘-tRF	10.812	10.846	6.2314	15.119
tRFdb-3002b	3‘-tRF	10.222	10.192	4.7455	14.543
tRFdb-3002a	3‘-tRF	10.513	10.485	5.0252	14.833
ts-42	tRF-1	10.812	10.789	6.5465	14.817
tRFdb-5026b	5‘-tRF	10.96	10.945	4.6838	17.915
tRFdb-5026c	5‘-tRF	10.529	10.514	4.2726	17.485
tRFdb-1024	tRF-1	11.178	11.204	6.3523	14.775
tRFdb-1036	tRF-1	11.343	11.253	6.3523	14.715
ts-50	tRF-1	10.349	10.323	5.4953	17.505
tRFdb-5024a	5‘-tRF	11.846	11.784	6.5754	17.398
5P_tRNA-Val-CAC-1-1	5‘U-tRF	10.167	10.017	5.4457	14.999
tRFdb-3008a	3‘-tRF	10.939	10.983	6.6031	17.1
tRFdb-3008b	3‘-tRF	10.567	10.611	6.2355	16.729
tRFdb-5024b	5‘-tRF	11.287	11.3	5.1605	16.928

tRFdb-5009a	5‘-tRF	10.985	11.213	6.6766	17.637
tRFdb-5009b	5‘-tRF	10.369	10.597	6.0674	17.02
tRFdb-5009c	5‘-tRF	9.9389	10.167	5.6443	16.589
ts-110	tRF-1	10.403	10.383	4.8341	14.93
tRFdb-5012b	5‘-tRF	9.7315	9.7027	6.0164	13.164
tRFdb-5013b	5‘-tRF	9.8459	9.8271	6.1457	13.238
tRFdb-5012a	5‘-tRF	10.04	10.075	6.4467	13.286
tRFdb-3004a	3‘-tRF	10.712	10.714	6.0841	13.863
ts-1	tRF-1	9.567	9.5756	4.4045	14.824
tRFdb-5001a	5‘-tRF	10.258	10.216	6.2622	14.599
tRFdb-5011a	5‘-tRF	10.182	10.179	6.2674	13.557
ts-6	tRF-1	9.69	9.6345	5.6743	12.731
5P_tRNA-Tyr-GTA-2-1	5‘U-tRF	9.7797	9.7497	5.8534	14.184
tRFdb-3011a	3‘-tRF	10.034	9.9601	5.0252	14.102
tRFdb-3011b	3‘-tRF	9.745	9.6711	4.7455	13.813
tRFdb-3026a	3‘-tRF	10.086	10.013	5.5944	15.847
tRFdb-3026b	3‘-tRF	9.7964	9.7243	5.3115	15.557
tRFdb-3007a	3‘-tRF	11.286	11.264	6.7325	16.689
ts-30	tRF-1	9.9619	9.9113	5.6371	13.627
5P_tRNA-Arg-ACG-2-1	5‘U-tRF	10.009	9.998	5.8038	14.576
tRFdb-5013c	5‘-tRF	9.1837	9.1507	5.6803	12.318
tRFdb-5014a	5‘-tRF	10.171	10.162	5.9532	14.728
ts-75	tRF-1	10.737	10.684	4.9681	16.07
5P_tRNA-Gly-CCC-2-1	5‘U-tRF	9.4499	9.3518	4.8167	12.029
tRFdb-5007a	5‘-tRF	9.8585	9.7597	5.2189	12.444
tRFdb-5007c	5‘-tRF	8.8601	8.7618	4.2571	11.444
tRFdb-3027a	3‘-tRF	9.5616	9.5401	5.1052	14.248
tRFdb-5004b	5‘-tRF	9.6525	9.4623	4.6843	13.421
tRFdb-5004c	5‘-tRF	9.1584	8.9688	4.2123	12.927
ts-88	tRF-1	8.8334	8.7758	4.3935	15.217
tRFdb-3027b	3‘-tRF	9.0684	9.0274	4.7455	12.236
5P_tRNA-Pro-AGG-2-1	5‘U-tRF	9.3642	9.3303	5.336	12.826
tRFdb-3014a	3‘-tRF	10.099	10.101	6.1712	14.058
tRFdb-3014b	3‘-tRF	9.8102	9.8121	5.8861	13.769
tRFdb-5016a	5‘-tRF	9.9352	9.9739	4.7688	14.769
tRFdb-1030	tRF-1	10.386	10.343	6.4309	14.299
ts-81	tRF-1	9.234	9.2251	4.5075	14.269
5P_tRNA-Leu-CAA-4-1	5‘U-tRF	9.2282	9.1928	4.3712	12.446
ts-38	tRF-1	9.0999	9.0145	4.0666	12.801
tRFdb-3009a	3‘-tRF	10.577	10.52	7.0142	13.459
tRFdb-3009b	3‘-tRF	10.298	10.254	6.7272	13.17
tRFdb-5015a	5‘-tRF	10.102	10.126	4.9681	14.976
tRFdb-5005a	5‘-tRF	9.6388	9.6158	5.5495	14.643
ts-55	tRF-1	9.2521	9.2813	4.6092	13.253
5P_tRNA-Lys-CTT-2-1	5‘U-tRF	9.3013	9.2306	4.9561	14.228
5P_tRNA-Val-AAC-1-1	5‘U-tRF	9.1167	9.0206	4.3712	11.897
tRFdb-3017a	3‘-tRF	10.784	10.85	6.4444	14.008
tRFdb-5008b	5‘-tRF	8.907	8.9358	4.6838	13.165
tRFdb-5003b	5‘-tRF	9.6314	9.7253	4.6843	14.447
tRFdb-5003c	5‘-tRF	9.1374	9.2316	4.2123	13.952
tRFdb-3017b	3‘-tRF	10.635	10.658	6.2355	13.78
ts-105	tRF-1	10.355	10.357	5.9444	13.484
tRFdb-3005a	3‘-tRF	10.175	10.391	6.0841	13.456
tRFdb-5008c	5‘-tRF	8.4198	8.4984	5.2912	12.782
ts-7	tRF-1	8.6798	8.7005	4.6092	11.497

tRFdb-3004b	3‘-tRF	9.8097	10.027	5.7184	13.084
tRFdb-3005b	3‘-tRF	9.8097	10.027	5.7184	13.084
ts-9	tRF-1	8.9967	9.1093	5.2613	15.541
tRFdb-3022a	3‘-tRF	9.4654	9.6361	5.7246	16.015
tRFdb-3022b	3‘-tRF	9.1764	9.3472	5.4412	15.725
5P_tRNA-Gly-GCC-2-1	5‘U-tRF	9.4724	9.3014	4.3712	12.608
5P_tRNA-Pro-CGG-1-1	5‘U-tRF	8.8465	8.9272	5.0434	12.352
5P_tRNA-Gly-GCC-2-2	5‘U-tRF	9.3159	9.1779	4.3712	12.781
tRFdb-3023a	3‘-tRF	9.223	9.1239	6.6404	13.343
ts-36	tRF-1	8.6915	8.6762	5.6192	13.689
ts-48	tRF-1	8.9926	9.0153	5.6057	13.744
tRFdb-3023b	3‘-tRF	8.8522	8.7474	6.2726	12.971
5P_tRNA-Ala-AGC-8-1	5‘U-tRF	9.2922	9.2335	5.9201	12.286
tRFdb-5023a	5‘-tRF	9.2351	9.083	4.6792	13.462
ts-60	tRF-1	8.8628	8.7587	5.3064	12.098
5P_tRNA-Gly-TCC-2-1	5‘U-tRF	8.853	8.8991	4.3712	13.038
tRFdb-5020a	5‘-tRF	9.5852	9.5467	4.8648	13.623
5P_tRNA-Lys-TTT-3-2	5‘U-tRF	9.1669	9.0843	6.0445	12.21
5P_tRNA-Val-CAC-4-1	5‘U-tRF	9.2205	9.1197	5.0434	15.321
5P_tRNA-His-GTG-1-1	5‘U-tRF	8.9397	8.8139	4.3712	13.89
ts-74	tRF-1	8.3921	8.2928	5.4844	12.033
5P_tRNA-Gly-TCC-2-6	5‘U-tRF	8.7462	8.677	4.9193	12.852
tRFdb-3031a	3‘-tRF	9.2626	9.7787	5.1052	16.055
tRFdb-3031b	3‘-tRF	8.445	8.9629	4.3175	15.236
tRFdb-1032	tRF-1	9.0899	8.889	5.3595	13.079
tRFdb-5023b	5‘-tRF	8.453	8.3676	4.18	12.939
5P_tRNA-Arg-TCT-1-1	5‘U-tRF	8.8883	8.7658	4.3712	13.55
ts-65	tRF-1	8.5976	8.5792	5.2062	11.564
ts-68	tRF-1	8.3901	8.3875	5.6743	11.063
tRFdb-5010a	5‘-tRF	10.242	10.308	5.9831	15.283
ts-89	tRF-1	8.3331	8.3557	4.8329	11.366
tRFdb-1040	tRF-1	8.8222	8.7908	5.3224	11.044
ts-12	tRF-1	8.6215	8.5103	4.555	13.501
5P_tRNA-His-GTG-1-9	5‘U-tRF	8.3617	8.383	4.9193	11.855
ts-67	tRF-1	8.046	7.9664	5.4875	10.433
ts-47	tRF-1	7.6406	7.6289	3.8207	11.629
tRFdb-3021b	3‘-tRF	8.5908	8.6888	4.9103	12.738
ts-43	tRF-1	7.654	7.6284	3.578	11.015
ts-80	tRF-1	7.1167	6.9856	3.4393	9.3207
5P_tRNA-His-GTG-1-7	5‘U-tRF	8.4476	8.3936	4.3712	12.775
ts-109	tRF-1	8.1126	7.9469	4.0666	10.517
ts-53	tRF-1	7.8624	7.85	4.4891	10.262
ts-108	tRF-1	6.9849	6.8941	3.8339	12.514
5P_tRNA-Leu-CAA-1-1	5‘U-tRF	8.6533	8.5797	4.3712	11.943
tRFdb-1031	tRF-1	8.5129	8.4614	4.6792	11.257
5P_tRNA-Asn-GTT-1-1	5‘U-tRF	8.619	8.5367	5.0434	12.999
tRFdb-3019a	3‘-tRF	8.277	8.2112	5.9532	10.432
tRFdb-3019b	3‘-tRF	7.9885	7.9229	5.6689	10.143
tRFdb-5021b	5‘-tRF	8.7084	8.6404	5.7089	13.151
ts-104	tRF-1	7.8698	7.8629	4.8898	11.425
5P_tRNA-Glu-TTC-1-1	5‘U-tRF	8.8479	8.8233	4.9561	11.688
ts-63	tRF-1	7.9866	7.9879	5.249	11.8
tRFdb-5021a	5‘-tRF	9.1148	9.0064	6.0653	11.655
ts-51	tRF-1	7.8389	7.8288	4.9902	11.444
tRFdb-5029a	5‘-tRF	8.9211	8.9891	5.1034	11.84

tRFdb-5029b	5‘-tRF	8.5683	8.6365	4.7614	11.486
ts-23	tRF-1	9.1616	9.065	4.5639	12.39
