# Supplementary material for: The tRNA-Cys-GCA Derived tsRNAs Suppress Tumor Progression of Gliomas via Regulating VAV2
Source: Dis Markers. 2022 Nov 15;2022:8708312. doi: 10.1155/2022/8708312 (PMC9681550; doi:10.1155/2022/8708312)
Supplement: Supplementary Materials — Figure S1: a summary of tsRNAs identification and characterization. (A) the flow chart of data processing and tsRNAs identification pipeline. (C) The characterization of tsRNA corresponding derived tRNA gene sources and (B) the chromosome locations statistical analysis. Figure S2: the enrichment analyses of tRFdb-3003b -related genes within TCGA-LGG datasets. (A) The top gene ontology (GO) terms, including biological process, CC (cellular component) and MF (molecular function), as well as the top KEGG pathway for tRFdb-3003b-related genes. (B) The correlation scatter-plots of tRFdb-3003b and its correlated-genes (ATG4B, LUC7L, D2HGDH, and HDAC10). (C-left) GSEA (gene set enrichment analysis) plots of three molecular signatures (chr6p21, microglia, and STIM treatment response signature), (C-right) the scatter plots for tRFdb-3003b and its correlated-genes (CRIP3 and ANKRD13B). Table S1: the primary clinical and molecular pathology characteristics parameters in glioma samples. Table S2: the primers used in quantitative real-time PCR assay. Table S3: a summary of the identified tsRNAs with available expression abundance in glioma datasets. Table S4: the expression profiles of tRNA-Cys-GCA derived tsRNAs with in glioma samples. [file 8708312.f1.zip › Supplemental Table S4.pdf]

Table S4. The expression profiles of tRNA-Cys-GCA derived tsRNAs with in glioma samples.

barcode	tRFdb-3003a	tRFdb-3003b	tRFdb-5015a	tRFdb-5016a	tRFdb-5017a	tRFdb-5017b	ts-55	ts-56	ts-60
TCGA-CS-4938-01B	15.879	15.507	NA	NA	NA	NA	NA	NA	7.848
TCGA-CS-4941-01A	12.342	11.971	10.923	10.716	13.381	12.497	10.98	9.101	NA
TCGA-CS-4942-01A	10.402	10.031	NA	NA	12.111	11.226	8.848	NA	NA
TCGA-CS-4943-01A	10.384	10.012	12.092	11.886	15.294	14.409	9.828	NA	NA
TCGA-CS-4944-01A	12.561	12.189	11.507	11.301	13.607	12.722	7.763	NA	NA
TCGA-CS-5390-01A	11.29	10.918	11.677	11.47	13.551	12.666	9.15	NA	9.209
TCGA-CS-5393-01A	11.497	11.125	11.562	11.355	14.021	13.136	10.62	NA	NA
TCGA-CS-5394-01A	11.752	11.38	9.5556	9.3495	9.5556	8.6727	NA	NA	10.99
TCGA-CS-5395-01A	13.698	13.326	NA	NA	NA	NA	9.821	NA	8.882
TCGA-CS-5396-01A	10.428	10.056	11.814	11.93	12.622	11.737	9.872	NA	NA
TCGA-CS-5397-01A	8.5781	8.2073	7.1641	6.9592	8.48	7.5989	NA	NA	5.306
TCGA-CS-6186-01A	11.594	11.222	NA	NA	8.9835	8.1014	9.04	NA	NA
TCGA-CS-6188-01A	14.196	13.824	8.4784	8.2726	9.0621	8.5936	NA	NA	NA
TCGA-CS-6290-01A	11.336	10.964	NA	NA	10.986	10.102	6.884	7.998	NA
TCGA-CS-6666-01A	14.426	14.054	NA	NA	12.533	11.649	NA	NA	NA
TCGA-CS-6667-01A	14.992	14.62	NA	NA	12.54	11.656	9.898	NA	8.958
TCGA-CS-6668-01A	15.593	15.221	11.2	10.993	NA	NA	NA	NA	NA
TCGA-CS-6669-01A	9.9454	9.5738	NA	NA	11.069	10.185	7.809	NA	8.865
TCGA-CS-6670-01A	13.114	12.742	NA	NA	12.049	11.165	NA	NA	NA
TCGA-DB-5270-01A	11.714	11.342	10.102	9.8955	14.27	13.386	10.16	NA	NA
TCGA-DB-5273-01A	9.8428	9.4713	NA	NA	14.688	13.803	NA	NA	NA
TCGA-DB-5274-01A	15.865	15.493	10.166	9.96	14.413	13.528	10.81	NA	9.283
TCGA-DB-5275-01A	15.266	14.894	NA	NA	11.953	11.069	12.01	NA	NA
TCGA-DB-5277-01A	11.209	10.837	10.596	10.39	12.596	11.711	NA	NA	NA
TCGA-DB-5278-01A	16.296	15.924	NA	NA	11.246	10.362	10.3	NA	NA
TCGA-DB-5279-01A	15.647	15.275	NA	NA	9.7872	8.9041	8.845	NA	NA
TCGA-DB-5280-01A	NA	NA	10.68	10.474	13.68	12.795	NA	NA	NA
TCGA-DB-5281-01A	16.355	15.983	NA	NA	12.835	11.951	10.31	NA	10.37
TCGA-DB-A4X9-01A	13.595	13.223	9.983	9.7768	13.069	12.185	NA	NA	NA
TCGA-DB-A4XA-01A	13.769	13.397	11.069	10.863	12.876	11.992	9.128	NA	NA
TCGA-DB-A4XB-01A	15.38	15.008	NA	10.861	NA	NA	NA	NA	NA
TCGA-DB-A4XC-01A	12.8	12.429	NA	NA	12.772	11.888	NA	NA	NA
TCGA-DB-A4XD-01A	14.965	14.593	10.652	10.446	11.652	10.768	9.71	NA	NA
TCGA-DB-A4XE-01A	15.31	14.938	11.79	11.584	15.249	14.365	9.848	NA	NA
TCGA-DB-A4XF-01A	15.072	14.7	12.186	11.98	15.06	14.176	NA	NA	NA
TCGA-DB-A4XG-01A	15.042	14.67	10.845	10.638	15.429	14.544	NA	NA	NA
TCGA-DB-A4XH-01A	12.583	12.212	13.633	13.427	16.385	15.501	NA	NA	NA
TCGA-DB-A64L-01A	13.485	13.114	NA	NA	13.065	12.181	NA	NA	NA
TCGA-DB-A64O-01A	12.246	11.875	NA	NA	10.634	9.7503	NA	NA	NA
TCGA-DB-A64P-01A	13.424	13.052	NA	NA	12.489	11.605	NA	NA	NA
TCGA-DB-A64Q-01A	13.735	13.363	NA	NA	11.315	10.431	10.37	NA	NA
TCGA-DB-A64R-01A	12.96	12.588	NA	NA	NA	NA	NA	NA	NA
TCGA-DB-A64S-01A	18.055	17.683	NA	NA	NA	NA	NA	NA	NA
TCGA-DB-A64U-01A	13.808	13.436	NA	NA	14.78	13.895	9.446	NA	NA
TCGA-DB-A64V-01A	16.626	16.254	12.621	12.415	NA	NA	NA	NA	NA
TCGA-DB-A64W-01A	14.257	13.886	11.323	11.117	12.908	12.023	10.38	NA	NA
TCGA-DB-A64X-01A	13.491	13.119	11.071	10.865	12.071	11.187	10.13	NA	NA
TCGA-DB-A75K-01A	14.851	14.479	NA	NA	12.473	11.589	NA	NA	8.271
TCGA-DB-A75L-01A	17.068	16.696	NA	NA	NA	NA	NA	NA	NA

TCGA-DB-A75O-01A	15.135	14.763	8.7312	8.5253	12.188	11.303	NA	NA	NA
TCGA-DB-A75P-01A	13.752	13.38	NA	NA	12.955	12.07	NA	NA	NA
TCGA-DH-5140-01A	15.109	14.737	12.497	12.29	12.497	11.612	12.67	NA	NA
TCGA-DH-5141-01A	16.002	15.63	NA	NA	14.296	13.411	13.25	NA	NA
TCGA-DH-5142-01A	10.881	10.509	11.683	11.477	14.683	13.798	8.742	NA	NA
TCGA-DH-5143-01A	11.754	11.382	12.141	11.935	12.878	11.994	NA	9.734	NA
TCGA-DH-5144-01A	11.92	11.548	NA	NA	10.401	9.5174	10.63	8.581	8.519
TCGA-DH-A669-01A	15.097	14.725	8.746	8.5401	13.471	12.586	8.389	NA	NA
TCGA-DH-A66B-01A	14.835	14.463	11.316	11.109	14.315	13.431	NA	NA	NA
TCGA-DH-A66D-01A	14.026	13.654	NA	NA	15.041	14.156	10.01	NA	NA
TCGA-DH-A66F-01A	12.758	12.386	10.824	10.617	13.145	12.261	NA	NA	NA
TCGA-DH-A66G-01A	14.875	14.503	NA	NA	12.94	12.056	12	NA	NA
TCGA-DH-A7UR-01A	14.031	13.659	10.718	10.512	13.525	12.641	9.776	NA	9.835
TCGA-DH-A7US-01A	13.536	13.164	10.465	10.258	12.049	11.165	NA	NA	NA
TCGA-DH-A7UT-01A	14.416	14.044	10.281	10.075	14.28	13.395	10.34	NA	9.397
TCGA-DH-A7UU-01A	14.444	14.072	NA	NA	15.831	14.946	NA	NA	NA
TCGA-DH-A7UV-01A	13.026	12.654	NA	NA	13.501	12.616	10.06	NA	NA
TCGA-DU-5847-01A	14.72	14.348	9.2764	9.3917	9.2764	8.3938	NA	NA	NA
TCGA-DU-5849-01A	9.5972	9.2258	NA	NA	8.9853	8.1031	NA	7.169	7.108
TCGA-DU-5852-01A	12.941	12.569	5.6495	5.4475	7.2152	6.3389	NA	4.848	NA
TCGA-DU-5853-01A	15.533	15.161	9.655	9.4489	NA	NA	7.717	NA	7.775
TCGA-DU-5854-01A	11.66	11.288	6.5082	6.3042	8.9546	8.0726	4.609	NA	NA
TCGA-DU-5855-01A	11.495	11.124	NA	NA	12.158	11.274	NA	7.817	7.756
TCGA-DU-5870-01A	18.199	17.827	NA	NA	NA	NA	NA	NA	NA
TCGA-DU-5871-01A	11.595	11.223	NA	NA	8.9846	8.1025	8.044	NA	7.108
TCGA-DU-5872-01A	11.338	10.967	NA	NA	9.5891	8.7062	8.647	7.45	8.706
TCGA-DU-5874-01A	9.8686	9.4971	7.7582	7.5528	8.7549	7.8732	6.242	6.94	5.319
TCGA-DU-6392-01A	9.4274	9.056	5.7518	5.5495	7.7316	6.8528	5.807	4.949	7.172
TCGA-DU-6393-01A	15.858	15.486	8.4937	8.2879	8.4937	7.6126	11.01	NA	11.69
TCGA-DU-6394-01A	8.7429	8.3719	7.9947	7.7891	7.2615	6.3849	4.777	4.893	7.115
TCGA-DU-6395-01A	11.677	11.305	NA	NA	9.329	8.4464	6.811	NA	6.87
TCGA-DU-6396-01A	11.473	11.102	NA	NA	11.745	10.861	NA	8.341	NA
TCGA-DU-6397-01A	9.9206	9.5491	7.4078	7.2026	7.4078	6.5304	NA	7.584	6.53
TCGA-DU-6399-01A	10.931	10.56	NA	NA	6.8707	5.9966	6.927	7.627	NA
TCGA-DU-6400-01A	11.502	11.13	NA	NA	9.0167	8.1345	9.488	6.621	9.132
TCGA-DU-6401-01A	11.841	11.469	NA	NA	NA	NA	NA	NA	NA
TCGA-DU-6402-01A	9.7537	9.3822	10.988	10.781	10.403	9.6348	NA	7.005	5.956
TCGA-DU-6403-01A	9.6152	9.2438	8.1311	7.9254	9.8645	8.9812	NA	5.745	6.671
TCGA-DU-6404-01A	10.899	10.527	7.0484	6.8437	NA	NA	7.686	7.806	7.163
TCGA-DU-6405-01A	10.157	9.7851	NA	NA	8.4591	7.8985	5.543	7.227	5.601
TCGA-DU-6406-01A	9.8586	9.4871	7.7149	7.7712	9.4756	8.8267	7.771	7.379	NA
TCGA-DU-6407-01A	16.421	16.049	9.0292	8.8232	9.6133	8.7303	NA	7.213	NA
TCGA-DU-6408-01A	10.607	10.236	8.6752	8.4692	NA	NA	NA	NA	NA
TCGA-DU-6410-01A	15.693	15.321	8.1078	8.6367	9.1052	8.2229	7.582	5.722	7.64
TCGA-DU-6542-01A	10.041	9.6693	NA	NA	9.4286	8.5459	8.487	8.607	NA
TCGA-DU-7006-01A	13.792	13.42	8.0453	8.8365	10.211	9.3274	7.688	NA	6.175
TCGA-DU-7007-01A	14.57	14.198	7.8444	8.6353	10.646	9.7626	NA	7.026	NA
TCGA-DU-7008-01A	10.575	10.204	NA	NA	NA	NA	8.437	NA	NA
TCGA-DU-7009-01A	11.248	10.876	NA	NA	13.094	12.209	NA	NA	NA
TCGA-DU-7010-01A	11.425	11.053	12.549	12.343	14.436	13.552	10.28	NA	NA
TCGA-DU-7012-01A	15.356	14.984	8.5371	8.3313	11.341	10.457	8.594	7.717	NA
TCGA-DU-7013-01A	15.917	15.546	NA	NA	9.2844	8.4019	NA	8.463	NA
TCGA-DU-7014-01A	11.59	11.218	NA	NA	12.562	11.678	8.231	7.355	7.294

TCGA-DU-7015-01A	13.963	13.591	NA	NA	NA	NA	NA	NA	NA
TCGA-DU-7018-01A	8.8238	8.4528	11.208	11.324	12.667	11.783	NA	NA	NA
TCGA-DU-7019-01A	12.202	11.83	NA	NA	13.266	12.382	10.91	NA	NA
TCGA-DU-7290-01A	10.947	10.575	10.334	10.128	10.334	9.4507	8.808	NA	7.87
TCGA-DU-7292-01A	15.523	15.151	NA	NA	9.3578	8.4752	NA	NA	8.475
TCGA-DU-7294-01A	15.509	15.137	10.027	9.8212	11.834	10.95	9.085	NA	NA
TCGA-DU-7298-01A	15.137	14.765	NA	NA	12.592	11.707	9.775	8.313	NA
TCGA-DU-7299-01A	10.573	10.201	7.9644	8.7555	9.5455	8.6626	NA	8.141	NA
TCGA-DU-7300-01A	10.859	10.487	9.925	9.7187	12.094	11.209	9.567	NA	NA
TCGA-DU-7301-01A	9.9544	9.5829	NA	8.1387	10.341	9.4575	8.401	NA	NA
TCGA-DU-7302-01A	14.805	14.433	9.6089	9.4028	14.308	13.423	NA	NA	NA
TCGA-DU-7304-01A	10.36	9.9882	9.426	9.2198	11.232	10.348	7.488	7.608	NA
TCGA-DU-7306-01A	13.707	13.335	NA	NA	9.7842	8.901	NA	NA	NA
TCGA-DU-7309-01A	15.72	15.348	NA	NA	12.522	11.638	NA	NA	NA
TCGA-DU-8158-01A	9.1398	8.7686	NA	7.3268	11.11	10.226	7.588	NA	NA
TCGA-DU-8161-01A	9.9295	9.558	8.3196	8.1138	11.316	10.432	NA	NA	NA
TCGA-DU-8162-01A	15.664	15.292	9.2209	10.013	13.026	12.142	NA	NA	8.338
TCGA-DU-8163-01A	15.051	14.679	NA	NA	12.718	11.834	7.694	NA	NA
TCGA-DU-8164-01A	16.394	16.022	8.9443	8.7383	10.527	9.6436	8.004	8.123	NA
TCGA-DU-8165-01A	14.779	14.407	8.5554	8.3495	11.289	10.542	NA	NA	6.103
TCGA-DU-8166-01A	10.666	10.294	NA	NA	9.7321	8.849	8.376	7.913	NA
TCGA-DU-8167-01A	15.031	14.659	6.9828	6.7781	12.181	11.297	7.039	NA	NA
TCGA-DU-8168-01A	15.479	15.107	10.167	10.545	11.751	10.867	9.809	NA	9.284
TCGA-DU-A5TP-01A	13.362	12.99	10.106	9.9002	12.629	11.745	NA	NA	NA
TCGA-DU-A5TR-01A	11.349	10.978	NA	NA	13.151	12.267	NA	NA	NA
TCGA-DU-A5TS-01A	16.911	16.539	14.976	14.769	NA	NA	NA	NA	NA
TCGA-DU-A5TT-01A	12.848	12.476	10.651	10.444	14.82	13.935	NA	NA	NA
TCGA-DU-A5TU-01A	12.782	12.41	12.17	11.963	12.17	11.285	NA	NA	NA
TCGA-DU-A5TW-01A	12.782	12.41	NA	NA	15.24	14.355	NA	NA	NA
TCGA-DU-A5TY-01A	12.711	12.339	12.291	12.085	14.291	13.406	NA	NA	NA
TCGA-DU-A6S2-01A	14.294	13.922	NA	NA	14.096	13.212	10.57	NA	NA
TCGA-DU-A6S3-01A	15.153	14.781	9.5537	9.3475	12.459	11.575	8.612	NA	8.671
TCGA-DU-A6S6-01A	15.597	15.225	10.756	10.55	NA	NA	11.62	NA	10.87
TCGA-DU-A6S7-01A	15.264	14.892	NA	NA	12.442	11.557	NA	NA	NA
TCGA-DU-A6S8-01A	13.961	13.589	9.7057	9.4995	13.874	12.99	NA	NA	8.823
TCGA-DU-A76K-01A	14.821	14.449	NA	NA	10.624	9.7403	NA	NA	9.74
TCGA-DU-A76L-01A	14.913	14.541	11.104	10.898	12.519	11.634	NA	NA	8.638
TCGA-DU-A76O-01A	16.891	16.519	13.717	13.511	NA	NA	NA	NA	NA
TCGA-DU-A76R-01A	15.047	14.675	NA	NA	11.225	10.341	NA	NA	9.342
TCGA-DU-A7T6-01A	15.329	14.957	NA	NA	12.073	11.188	NA	NA	NA
TCGA-DU-A7T8-01A	14.328	13.957	10.716	10.51	13.038	12.153	NA	NA	NA
TCGA-DU-A7TA-01A	15.167	14.795	NA	NA	NA	NA	NA	NA	NA
TCGA-DU-A7TB-01A	13.491	13.119	NA	NA	12.293	11.409	NA	NA	NA
TCGA-DU-A7TC-01A	16.224	15.852	NA	12.704	NA	NA	NA	NA	NA
TCGA-DU-A7TD-01A	13.838	13.466	9.3939	9.1878	10.715	9.8308	NA	NA	NA
TCGA-DU-A7TG-01A	13.367	12.995	NA	NA	13.206	12.322	NA	NA	10.21
TCGA-DU-A7TI-01A	15.222	14.85	10.025	9.8192	12.024	11.14	NA	NA	NA
TCGA-DU-A7TJ-01A	16.446	16.074	NA	NA	11.279	10.395	NA	NA	NA
TCGA-E1-5302-01A	14.71	14.338	13.512	13.305	NA	NA	NA	NA	NA
TCGA-E1-5303-01A	11.139	10.767	11.356	11.32	15.464	14.58	NA	NA	NA
TCGA-E1-5304-01A	14.006	13.635	9.81	9.6038	15.13	14.246	8.868	NA	NA
TCGA-E1-5305-01A	11.556	11.184	NA	NA	10.721	9.8374	9.194	NA	NA
TCGA-E1-5307-01A	13.956	13.585	NA	NA	10.537	9.6533	NA	NA	NA

TCGA-E1-5311-01A	12.624	12.252	NA	NA	10.427	9.5429	11.07	9.604	9.543
TCGA-E1-5318-01A	13.633	13.261	NA	NA	13.02	12.136	13.08	NA	NA
TCGA-E1-5319-01A	14.73	14.358	NA	NA	13.03	12.146	NA	9.209	NA
TCGA-E1-5322-01A	14.001	13.629	NA	NA	12.71	11.825	NA	NA	NA
TCGA-E1-A7YD-01A	12.948	12.576	7.1163	6.9114	8.1111	7.231	NA	NA	NA
TCGA-E1-A7YE-01A	16.105	15.733	NA	NA	NA	NA	NA	NA	NA
TCGA-E1-A7YH-01A	15.983	15.611	10.727	10.52	12.726	11.842	9.784	NA	NA
TCGA-E1-A7YI-01A	15.673	15.301	NA	NA	NA	NA	NA	NA	NA
TCGA-E1-A7YJ-01A	13.051	12.679	NA	11.232	13.023	12.139	NA	NA	NA
TCGA-E1-A7YK-01A	13.837	13.466	10.766	10.559	15.157	14.273	NA	NA	NA
TCGA-E1-A7YL-01A	13.322	12.95	11.387	12.18	13.387	12.502	NA	10.56	NA
TCGA-E1-A7YM-01A	14.72	14.348	9.4655	9.2593	14.593	13.708	NA	NA	NA
TCGA-E1-A7YN-01A	13.083	12.711	NA	NA	11.77	10.886	7.251	NA	8.305
TCGA-E1-A7YO-01A	16.52	16.148	10.4	10.194	13.858	12.974	NA	NA	9.516
TCGA-E1-A7YQ-01A	12.759	12.387	9.6239	9.4177	13.48	12.596	NA	NA	NA
TCGA-E1-A7YS-01A	16.197	15.825	11.912	11.706	13.719	12.835	9.97	NA	NA
TCGA-E1-A7YU-01A	13.007	12.635	11.658	11.451	11.658	10.773	NA	NA	NA
TCGA-E1-A7YV-01A	14.157	13.785	NA	NA	12.606	11.722	NA	NA	NA
TCGA-E1-A7YW-01A	14.747	14.375	NA	NA	12.075	11.191	NA	NA	NA
TCGA-E1-A7YY-01A	15.301	14.929	11.519	11.313	13.104	12.219	11.58	NA	10.63
TCGA-E1-A7Z2-01A	15.863	15.491	NA	NA	NA	NA	NA	NA	10.2
TCGA-E1-A7Z3-01A	14.413	14.041	NA	NA	12.479	11.594	NA	NA	NA
TCGA-E1-A7Z4-01A	12.772	12.4	NA	NA	11.574	10.69	NA	NA	NA
TCGA-E1-A7Z6-01A	14.61	14.238	NA	NA	11.91	11.026	NA	NA	NA
TCGA-EZ-7264-01A	8.8864	8.5153	NA	NA	10.272	9.388	8.331	NA	NA
TCGA-F6-A8O3-01A	14.921	14.549	NA	NA	9.2661	8.3836	NA	NA	NA
TCGA-F6-A8O4-01A	11.237	10.866	7.875	7.6695	6.8812	6.007	NA	NA	NA
TCGA-FG-5962-01B	10.88	10.508	NA	NA	9.0985	8.2162	NA	NA	8.8
TCGA-FG-5963-01A	14.294	13.922	4.9681	4.7688	7.9272	7.5042	8.304	5.14	5.651
TCGA-FG-5964-01A	15.291	14.919	NA	NA	NA	NA	NA	NA	8.819
TCGA-FG-5965-01B	16.028	15.657	NA	NA	8.9427	8.0607	8.999	NA	8.061
TCGA-FG-6688-01A	8.249	7.8785	NA	NA	9.6331	8.7501	NA	NA	NA
TCGA-FG-6689-01A	10.446	10.074	10.418	10.211	9.8332	8.9501	8.891	NA	NA
TCGA-FG-6690-01A	10.737	10.366	NA	NA	NA	NA	9.183	NA	9.241
TCGA-FG-6691-01A	10.075	9.703	9.8768	10.67	12.046	11.161	7.938	NA	NA
TCGA-FG-6692-01A	10.062	9.6906	8.4519	8.8295	9.0355	8.1533	8.829	NA	6.578
TCGA-FG-7634-01A	7.7285	7.3585	NA	NA	8.1139	7.2338	NA	NA	NA
TCGA-FG-7636-01A	15.466	15.094	9.4623	9.2561	11.783	10.898	8.521	NA	NA
TCGA-FG-7637-01A	16.147	15.775	NA	NA	13.693	12.808	NA	NA	NA
TCGA-FG-7638-01B	10.251	9.8793	NA	NA	9.0545	8.1723	NA	NA	NA
TCGA-FG-7641-01B	10.612	10.241	NA	NA	8.6799	7.7984	NA	NA	7.798
TCGA-FG-7643-01A	15.297	14.925	NA	NA	12.225	11.341	9.283	NA	NA
TCGA-FG-8181-01A	10.044	9.6721	8.2641	8.0584	10.068	9.1847	7.325	NA	7.384
TCGA-FG-8182-01A	10.542	10.17	10.665	10.459	11.343	10.459	NA	NA	7.466
TCGA-FG-8185-01A	11.019	10.647	NA	NA	NA	NA	NA	NA	NA
TCGA-FG-8186-01A	8.491	8.1202	7.8801	7.6746	8.877	7.9951	NA	NA	NA
TCGA-FG-8187-01A	10.317	9.9454	8.1231	7.9175	11.288	10.404	7.184	NA	NA
TCGA-FG-8188-01A	15.821	15.449	9.9802	9.774	10.98	10.096	NA	8.161	NA
TCGA-FG-8189-01B	11.922	11.55	13.309	13.102	13.894	13.009	NA	NA	NA
TCGA-FG-8191-01A	8.9664	8.5952	9.3527	9.8825	10.352	9.468	NA	7.948	NA
TCGA-FG-A4MU-01B	13.718	13.346	11.427	11.221	11.105	10.221	NA	NA	NA
TCGA-FG-A4MW-01A	13.792	13.42	10.957	10.751	13.693	12.809	NA	NA	8.491
TCGA-FG-A4MX-01A	13.599	13.227	9.5953	9.3892	15.559	14.675	NA	NA	NA

TCGA-FG-A4MY-01A	14.222	13.85	12.439	12.233	16.024	15.139	NA	NA	NA
TCGA-FG-A60J-01A	15.269	14.897	NA	NA	13.112	12.227	9.585	NA	9.644
TCGA-FG-A60K-01A	13.67	13.298	NA	NA	12.182	11.298	NA	NA	NA
TCGA-FG-A60L-01A	13.825	13.453	NA	NA	14.424	13.539	NA	NA	NA
TCGA-FG-A6IZ-01A	13.542	13.17	10.708	10.501	11.122	10.238	8.183	NA	NA
TCGA-FG-A6J1-01A	13.277	12.906	NA	NA	14.13	13.246	NA	NA	8.392
TCGA-FG-A6J3-01A	12.899	12.527	9.2012	9.9937	11.521	10.637	NA	NA	NA
TCGA-FG-A70Y-01A	16.249	15.877	NA	NA	12.408	11.523	11.46	NA	NA
TCGA-FG-A70Z-01A	15.336	14.964	9.7025	9.4963	10.702	9.8179	NA	NA	NA
TCGA-FG-A710-01A	15.286	14.914	10.182	9.9757	10.182	9.2985	NA	NA	NA
TCGA-FG-A711-01A	15.644	15.272	NA	NA	11.173	10.289	10.23	NA	NA
TCGA-FG-A713-01A	15.055	14.683	NA	NA	13.725	12.841	NA	10.1	NA
TCGA-FG-A87N-01A	16.079	15.707	NA	NA	10.338	9.4544	10.39	NA	NA
TCGA-FG-A87Q-01A	14.479	14.107	9.9603	9.754	11.959	11.075	NA	NA	NA
TCGA-FN-7833-01A	16.077	15.705	8.9912	8.7852	8.9912	8.1091	NA	8.17	NA
TCGA-HT-7467-01A	13.879	13.507	11.588	11.382	12.725	11.841	9.324	NA	8.385
TCGA-HT-7468-01A	9.1348	8.7636	NA	NA	10.106	9.2221	NA	NA	NA
TCGA-HT-7469-01A	15.25	14.878	NA	NA	8.8583	7.9764	10.72	NA	7.976
TCGA-HT-7470-01A	15.694	15.322	NA	NA	11.159	10.275	8.633	NA	NA
TCGA-HT-7471-01A	16.203	15.831	NA	NA	11.029	10.145	8.09	NA	NA
TCGA-HT-7472-01A	11.81	11.438	9.8759	9.6697	10.875	9.9913	8.934	NA	NA
TCGA-HT-7473-01A	14.742	14.37	NA	NA	9.3761	8.4934	10.43	NA	NA
TCGA-HT-7474-01A	10.778	10.406	NA	NA	10.58	9.6965	8.639	NA	NA
TCGA-HT-7475-01A	12.773	12.401	NA	NA	10.353	9.4698	10.87	NA	11.28
TCGA-HT-7476-01A	15.498	15.126	10.176	9.97	12.497	11.613	10.82	NA	12.1
TCGA-HT-7477-01B	12.981	12.609	NA	NA	12.046	11.162	NA	NA	NA
TCGA-HT-7478-01A	11.054	10.682	NA	NA	12.44	11.556	NA	NA	NA
TCGA-HT-7479-01A	13.875	13.503	10.356	10.15	12.814	11.93	8.416	NA	NA
TCGA-HT-7480-01A	17.501	17.129	12.397	12.19	NA	NA	NA	NA	NA
TCGA-HT-7481-01A	14.347	13.975	10.565	10.359	14.319	13.435	8.624	NA	8.683
TCGA-HT-7482-01A	11.612	11.24	NA	NA	10.263	9.3792	9.735	NA	NA
TCGA-HT-7483-01A	11.455	11.083	NA	NA	10.62	9.7359	9.677	NA	8.154
TCGA-HT-7485-01A	15.395	15.023	9.4444	10.237	10.443	9.5597	8.503	NA	8.562
TCGA-HT-7601-01A	15.715	15.343	10.984	11.1	13.739	12.854	NA	NA	NA
TCGA-HT-7602-01A	9.1229	8.7517	NA	NA	12.093	11.208	NA	NA	NA
TCGA-HT-7603-01A	16.175	15.803	NA	NA	11.289	10.405	NA	9.882	9.821
TCGA-HT-7604-01A	10.494	10.122	NA	NA	NA	NA	NA	NA	NA
TCGA-HT-7605-01A	16.3	15.928	9.0599	9.8523	9.0599	8.1777	8.119	8.239	9.175
TCGA-HT-7606-01A	9.3174	8.9461	NA	NA	NA	NA	NA	NA	NA
TCGA-HT-7607-01A	10.706	10.334	NA	NA	9.509	8.6261	NA	NA	NA
TCGA-HT-7608-01A	9.9821	9.6105	NA	NA	10.369	9.4852	NA	NA	NA
TCGA-HT-7609-01A	10.134	9.7628	8.9381	8.7321	10.521	9.6374	NA	NA	NA
TCGA-HT-7610-01A	15.066	14.694	9.3677	9.1616	11.688	10.804	8.426	NA	9.483
TCGA-HT-7611-01A	15.065	14.693	9.3247	9.1186	12.782	11.898	9.381	NA	NA
TCGA-HT-7616-01A	8.845	8.474	6.6583	6.454	10.452	9.5687	7.707	NA	NA
TCGA-HT-7620-01A	11.36	10.988	8.051	7.8454	8.051	7.1711	9.105	NA	NA
TCGA-HT-7676-01A	11.15	10.778	NA	NA	9.337	8.4544	9.772	NA	6.149
TCGA-HT-7677-01A	16.565	16.193	NA	NA	8.9273	8.0453	10.79	NA	8.045
TCGA-HT-7680-01A	14.135	13.764	9.3414	9.1353	10.755	9.8712	7.817	NA	NA
TCGA-HT-7681-01A	10.902	10.531	9.9683	9.762	13.137	12.253	9.026	NA	8.088
TCGA-HT-7684-01A	9.7395	9.368	NA	8.3379	10.541	9.6571	NA	NA	NA
TCGA-HT-7686-01A	11.143	10.771	9.6567	9.865	13.355	12.471	8.715	NA	NA
TCGA-HT-7687-01A	9.1537	8.7825	9.5401	9.6555	11.626	10.742	NA	NA	7.661

TCGA-HT-7688-01A	16.106	15.734	10.295	10.089	11.616	10.732	8.355	NA	9.411
TCGA-HT-7689-01A	10.818	10.446	8.2093	8.0036	9.791	8.9079	8.266	NA	NA
TCGA-HT-7690-01A	11.152	10.781	NA	NA	8.0847	7.2047	8.724	NA	NA
TCGA-HT-7691-01A	10.437	10.066	8.5053	8.2994	9.0889	8.2066	9.56	NA	NA
TCGA-HT-7692-01A	14.831	14.459	NA	NA	11.089	10.205	7.568	NA	NA
TCGA-HT-7693-01A	15.058	14.686	NA	NA	10.861	9.977	8.599	NA	7.661
TCGA-HT-7694-01A	14.665	14.293	7.7189	7.5135	9.2993	8.4167	7.775	NA	9.415
TCGA-HT-7695-01A	15.762	15.39	9.1073	8.9012	12.427	11.543	8.166	NA	10.22
TCGA-HT-7854-01A	15.887	15.515	9.1788	8.9728	11.762	10.878	8.238	NA	9.878
TCGA-HT-7855-01A	15.092	14.72	NA	NA	13.374	12.49	9.112	NA	NA
TCGA-HT-7856-01A	11.374	11.002	7.7669	7.5615	11.346	10.462	8.82	NA	7.882
TCGA-HT-7857-01A	12.42	12.048	9.6196	9.9103	12.16	11.29	4.646	NA	NA
TCGA-HT-7858-01A	9.3973	9.026	NA	6.2726	10.047	9.1633	NA	NA	5.606
TCGA-HT-7860-01A	15.752	15.38	9.6328	9.4267	8.6346	7.7532	NA	8.811	7.753
TCGA-HT-7873-01B	14.955	14.583	NA	8.7635	NA	NA	11.02	NA	9.085
TCGA-HT-7874-01A	11.603	11.231	9.9907	9.7844	11.312	10.428	NA	NA	9.107
TCGA-HT-7875-01A	16.615	16.243	9.3464	9.1403	10.93	10.046	NA	NA	NA
TCGA-HT-7877-01A	15.542	15.17	NA	NA	11.305	10.421	9.778	NA	10.42
TCGA-HT-7879-01A	15.707	15.335	8.4044	8.1986	8.4044	7.5234	11.04	NA	7.523
TCGA-HT-7880-01A	15.516	15.144	NA	NA	11.788	10.904	11.13	NA	10.73
TCGA-HT-7881-01A	11.47	11.098	NA	7.6575	9.8583	8.9751	9.5	NA	7.978
TCGA-HT-7882-01A	15.964	15.592	NA	10.907	9.1152	8.2329	10.49	NA	NA
TCGA-HT-7884-01B	14.591	14.219	NA	NA	8.8516	7.9697	11.71	NA	7.97
TCGA-HT-7902-01A	10.689	10.317	8.9622	8.7562	10.545	9.6616	8.021	NA	6.506
TCGA-HT-8010-01A	15.327	14.955	9.4493	9.2432	11.77	10.885	NA	NA	9.565
TCGA-HT-8011-01A	15.456	15.085	11.181	11.39	9.5974	8.7144	NA	NA	NA
TCGA-HT-8012-01A	16.473	16.101	9.1898	8.9837	12.358	11.473	9.83	NA	8.307
TCGA-HT-8013-01A	11.238	10.867	NA	NA	11.532	10.648	8.271	NA	NA
TCGA-HT-8015-01B	13.897	13.525	NA	NA	15.073	14.188	NA	NA	NA
TCGA-HT-8018-01A	9.54	9.1686	8.514	8.7221	10.511	9.6274	NA	NA	6.062
TCGA-HT-8019-01A	14.987	14.615	NA	9.4413	12.346	11.462	7.709	NA	11.76
TCGA-HT-8104-01A	14.163	13.791	10.743	10.537	10.743	9.8595	10.8	8.922	8.861
TCGA-HT-8105-01A	10.333	9.9615	NA	NA	11.135	10.251	NA	NA	9.252
TCGA-HT-8107-01A	12.234	11.862	8.9235	8.7175	11.243	10.359	9.979	NA	10.84
TCGA-HT-8108-01A	15.406	15.034	8.4658	9.2576	11.047	10.163	11.1	NA	7.585
TCGA-HT-8109-01A	16.191	15.819	NA	NA	10.636	9.7525	9.694	NA	10.56
TCGA-HT-8110-01A	12.647	12.275	6.0555	5.8524	7.0447	6.1694	NA	NA	NA
TCGA-HT-8111-01A	11.05	10.678	NA	6.8502	9.853	8.9698	8.911	NA	7.17
TCGA-HT-8113-01A	10.853	10.482	8.9206	8.7146	11.918	11.034	NA	NA	9.62
TCGA-HT-8114-01A	15.842	15.47	9.9085	9.7023	12.229	11.345	11.55	NA	NA
TCGA-HT-8558-01A	10.776	10.405	NA	NA	NA	NA	7.641	NA	8.697
TCGA-HT-8563-01A	13.505	13.133	9.3227	9.4057	12.034	11.149	8.381	NA	7.857
TCGA-HT-8564-01A	10.43	10.058	NA	NA	8.7038	7.8222	8.177	NA	7.24
TCGA-HT-A4DS-01A	14.298	13.926	NA	NA	11.948	11.064	10.42	NA	NA
TCGA-HT-A4DV-01A	14.143	13.771	8.9482	8.7422	13.193	12.309	NA	NA	NA
TCGA-HT-A5R5-01A	14.288	13.917	NA	NA	12.506	11.621	8.98	NA	NA
TCGA-HT-A5R7-01A	13.922	13.55	11.724	11.518	13.531	12.647	NA	NA	11.42
TCGA-HT-A5R9-01A	12.608	12.236	12.995	12.788	NA	NA	NA	NA	NA
TCGA-HT-A5RA-01A	13.347	12.975	10.413	10.207	11.412	10.528	9.471	NA	NA
TCGA-HT-A5RB-01A	13.63	13.258	NA	NA	13.21	12.325	NA	NA	NA
TCGA-HT-A5RC-01A	13.238	12.866	11.626	11.419	13.947	13.063	NA	NA	NA
TCGA-HT-A614-01A	15.678	15.306	NA	NA	NA	NA	NA	NA	NA
TCGA-HT-A615-01A	13.197	12.825	11	10.793	NA	NA	10.06	NA	NA

TCGA-HT-A616-01A	12.999	12.627	13.386	13.179	14.971	14.086	NA	NA	NA
TCGA-HT-A617-01A	12.646	12.274	NA	NA	14.518	13.634	9.769	NA	NA
TCGA-HT-A618-01A	11.392	11.021	9.4589	9.2528	11.779	10.895	8.517	NA	NA
TCGA-HT-A619-01A	14.306	13.934	NA	NA	11.787	10.903	10.84	NA	10.9
TCGA-HT-A61A-01A	12.648	12.277	NA	NA	13.62	12.736	NA	NA	11.74
TCGA-HT-A61B-01A	12.225	11.853	NA	NA	13.612	12.727	8.864	NA	NA
TCGA-HT-A61C-01A	14.035	13.663	12.007	12.215	13.229	12.345	NA	NA	NA
TCGA-HT-A74H-01A	14.567	14.195	10.049	9.8424	10.049	9.1653	9.107	NA	NA
TCGA-HT-A74K-01A	15.275	14.903	10.171	9.9648	14.418	13.533	9.229	NA	9.288
TCGA-HT-A74L-01A	15.462	15.09	NA	NA	10.873	9.989	NA	NA	NA
TCGA-HT-A74O-01A	14.868	14.496	NA	NA	14.151	13.267	9.887	NA	NA
TCGA-HW-7486-01A	10.867	10.495	8.9341	9.4637	11.517	10.633	7.411	6.539	6.478
TCGA-HW-7487-01A	12.204	11.832	NA	NA	NA	NA	11.33	NA	NA
TCGA-HW-7489-01A	14.172	13.8	10.036	9.8302	11.036	10.152	9.094	NA	9.153
TCGA-HW-7490-01A	13.784	13.412	NA	NA	NA	NA	NA	NA	NA
TCGA-HW-7491-01A	7.7406	7.3706	NA	NA	10.444	9.5602	NA	NA	NA
TCGA-HW-7493-01A	14.541	14.169	8.4242	8.2184	10.421	9.859	NA	NA	NA
TCGA-HW-7495-01A	9.6875	9.3161	NA	NA	9.0755	8.1932	8.135	NA	NA
TCGA-HW-8319-01A	14.938	14.566	NA	NA	10.607	9.7231	11.08	NA	9.723
TCGA-HW-8320-01A	10.483	10.111	NA	NA	NA	NA	NA	6.888	NA
TCGA-HW-8321-01A	16.349	15.977	NA	8.6085	11.397	10.513	8.871	NA	7.933
TCGA-HW-8322-01A	10.132	9.7607	7.939	8.73	10.934	10.05	8.578	NA	8.054
TCGA-HW-A5KJ-01A	12.82	12.448	9.886	9.6798	15.344	14.459	9.943	NA	NA
TCGA-HW-A5KK-01A	13.891	13.519	11.279	11.072	15.366	14.481	NA	NA	NA
TCGA-HW-A5KL-01A	14.573	14.201	12.79	12.806	NA	NA	NA	NA	NA
TCGA-HW-A5KM-01A	14.04	13.668	11.956	11.749	16.502	15.617	NA	NA	NA
TCGA-IK-7675-01A	10.679	10.307	8.7465	8.5405	8.7465	7.8648	9.801	NA	NA
TCGA-IK-8125-01A	10.291	9.919	8.68	8.4741	9.6783	8.7953	7.74	NA	NA
TCGA-KT-A74X-01A	14.943	14.571	10.687	10.481	11.102	10.218	11.16	NA	NA
TCGA-KT-A7W1-01A	14.4	14.028	13.929	13.81	13.836	12.952	NA	NA	NA
TCGA-P5-A5ET-01A	14.18	13.808	11.508	11.302	14.567	13.682	8.982	NA	9.041
TCGA-P5-A5EU-01A	13.672	13.3	11.557	11.351	15.557	14.672	NA	NA	NA
TCGA-P5-A5EV-01A	14.299	13.927	11.317	11.333	14.776	13.892	7.795	NA	NA
TCGA-P5-A5EW-01A	13.547	13.175	NA	NA	14.403	13.519	NA	NA	NA
TCGA-P5-A5EX-01A	12.786	12.414	9.0602	8.8541	12.058	11.174	NA	NA	6.603
TCGA-P5-A5EY-01A	12.96	12.588	9.8892	9.6829	16.154	15.27	8.947	NA	9.006
TCGA-P5-A5EZ-01A	11.582	11.21	11.121	10.915	14.753	13.869	NA	NA	NA
TCGA-P5-A5F0-01A	14.397	14.025	11.562	11.355	13.677	12.792	9.036	NA	10.09
TCGA-P5-A5F1-01A	17.272	16.9	NA	NA	NA	NA	NA	NA	NA
TCGA-P5-A5F2-01A	12.645	12.273	NA	NA	14.032	13.148	NA	NA	NA
TCGA-P5-A5F4-01A	16.826	16.454	NA	NA	NA	NA	NA	NA	NA
TCGA-P5-A5F6-01A	10.556	10.184	11.205	10.999	11.62	10.906	NA	NA	NA
TCGA-P5-A72U-01A	14.601	14.229	10.902	10.695	NA	NA	9.959	NA	NA
TCGA-P5-A72W-01A	13.6	13.228	NA	NA	12.987	12.103	8.463	NA	NA
TCGA-P5-A72X-01A	15.351	14.979	NA	NA	11.057	10.173	8.532	NA	10.17
TCGA-P5-A72Z-01A	15.385	15.013	NA	NA	10.729	9.8449	9.786	NA	NA
TCGA-P5-A730-01A	15.568	15.196	NA	NA	13.613	12.728	9.863	NA	8.924
TCGA-P5-A731-01A	13.999	13.627	10.579	11.373	NA	NA	NA	NA	NA
TCGA-P5-A733-01A	15.824	15.452	8.7303	8.5243	10.728	9.8439	NA	NA	NA
TCGA-P5-A735-01A	17.629	17.257	NA	NA	NA	NA	NA	NA	NA
TCGA-P5-A736-01A	14.267	13.895	NA	NA	12.484	11.6	10.54	NA	11.6
TCGA-P5-A737-01A	12.163	11.791	8.7459	8.54	12.743	11.858	NA	NA	NA
TCGA-P5-A77W-01A	15.386	15.014	NA	NA	12.826	11.941	10.4	NA	9.136

TCGA-P5-A77X-01A	14.721	14.349	11.234	11.028	13.556	12.672	9.707	NA	10.35
TCGA-P5-A780-01A	15.259	14.887	NA	9.7406	14.945	14.061	9.005	NA	9.063
TCGA-P5-A781-01A	14.791	14.419	11.534	11.328	13.704	12.82	9.592	NA	NA
TCGA-QH-A65R-01A	13.786	13.414	11.851	11.645	14.253	13.369	NA	NA	NA
TCGA-QH-A65S-01A	13.577	13.205	NA	NA	13.812	12.927	NA	NA	NA
TCGA-QH-A65V-01A	14.749	14.377	NA	NA	14.037	13.152	NA	NA	NA
TCGA-QH-A65X-01A	16.914	16.542	NA	NA	12.842	11.958	9.316	NA	9.375
TCGA-QH-A65Z-01A	13.698	13.326	NA	NA	14.223	13.338	9.821	NA	NA
TCGA-QH-A6CS-01A	14.237	13.865	12.039	11.833	15.498	14.614	NA	NA	NA
TCGA-QH-A6CU-01A	14.551	14.179	11.238	11.616	11.238	10.354	NA	NA	NA
TCGA-QH-A6CV-01A	13.67	13.298	11.969	11.763	15.014	14.129	NA	NA	NA
TCGA-QH-A6CX-01A	12.877	12.505	7.5436	7.3383	10.859	9.9747	NA	NA	NA
TCGA-QH-A6CY-01A	14.296	13.924	NA	NA	12.277	11.393	NA	NA	NA
TCGA-QH-A6CZ-01A	18.387	18.016	12.527	12.32	NA	NA	NA	NA	NA
TCGA-QH-A6X3-01A	16.509	16.137	NA	8.9709	10.76	9.8765	NA	8.356	NA
TCGA-QH-A6X4-01A	17.048	16.676	11.188	10.981	NA	NA	NA	NA	NA
TCGA-QH-A6X5-01A	14.34	13.968	10.955	11.164	12.37	11.486	NA	NA	NA
TCGA-QH-A6X8-01A	15.838	15.466	10.295	10.089	12.294	11.41	NA	NA	NA
TCGA-QH-A6X9-01A	14.093	13.721	10.574	10.367	12.573	11.689	NA	NA	NA
TCGA-QH-A6XA-01A	14.486	14.114	NA	NA	10.873	9.9895	NA	NA	NA
TCGA-QH-A6XC-01A	15.258	14.886	9.5384	9.3323	12.707	11.822	NA	NA	NA
TCGA-QH-A86X-01A	15.222	14.85	9.2535	9.0474	9.2535	8.371	NA	NA	NA
TCGA-QH-A870-01A	15.565	15.193	NA	NA	10.239	9.3558	NA	NA	8.358
TCGA-R8-A6MK-01A	15.505	15.133	10.192	9.9862	11.192	10.308	NA	NA	NA
TCGA-R8-A6ML-01A	15.649	15.277	NA	NA	10.545	9.6613	NA	NA	9.661
TCGA-R8-A6MO-01A	16.139	15.767	NA	NA	NA	NA	NA	NA	NA
TCGA-R8-A6YH-01A	15.578	15.206	NA	NA	11.011	10.127	NA	NA	NA
TCGA-R8-A73M-01A	15.399	15.028	NA	NA	11.202	10.318	NA	9.381	NA
TCGA-RY-A83X-01A	16.005	15.633	NA	10.017	NA	NA	9.281	NA	10.34
TCGA-RY-A83Y-01A	14.617	14.245	NA	NA	9.6311	8.7481	9.273	NA	7.751
TCGA-RY-A83Z-01A	15.821	15.449	NA	NA	11.609	10.725	11.25	NA	9.726
TCGA-RY-A840-01A	15.943	15.571	NA	NA	11.63	10.746	NA	NA	8.165
TCGA-RY-A843-01A	16.661	16.289	NA	NA	NA	NA	NA	NA	NA
TCGA-RY-A845-01A	14.909	14.537	NA	NA	10.542	9.6582	9.599	NA	NA
TCGA-RY-A847-01A	10.556	10.184	7.7782	7.8346	9.4518	8.569	NA	NA	NA
TCGA-S9-A6TS-01A	16.928	16.556	NA	NA	NA	NA	NA	NA	NA
TCGA-S9-A6TU-01A	14.045	13.673	10.389	10.182	13.973	13.088	NA	NA	NA
TCGA-S9-A6TV-01A	15.484	15.112	NA	NA	10.989	10.105	10.63	NA	NA
TCGA-S9-A6TW-01A	15.377	15.005	NA	NA	12.35	11.465	11.14	NA	NA
TCGA-S9-A6TX-01A	15.528	15.156	10.425	10.218	13.424	12.539	NA	NA	NA
TCGA-S9-A6TY-01A	15.167	14.795	NA	NA	12.467	11.583	NA	NA	NA
TCGA-S9-A6TZ-01A	15.153	14.781	NA	NA	12.84	11.955	NA	NA	NA
TCGA-S9-A6U0-01A	14.277	13.905	11.416	11.532	12.875	11.991	NA	NA	NA
TCGA-S9-A6U1-01A	18.121	17.749	NA	NA	NA	NA	NA	NA	NA
TCGA-S9-A6U2-01A	16.044	15.672	NA	NA	10.788	9.9045	NA	NA	NA
TCGA-S9-A6U5-01A	18.602	18.23	NA	NA	NA	NA	NA	NA	NA
TCGA-S9-A6U6-01A	14.558	14.186	NA	NA	12.576	11.691	NA	NA	NA
TCGA-S9-A6U8-01A	15.341	14.969	NA	NA	12.028	11.144	NA	NA	NA
TCGA-S9-A6U9-01A	15.263	14.891	NA	NA	NA	NA	NA	NA	NA
TCGA-S9-A6UA-01A	13.538	13.166	NA	NA	14.588	13.703	9.399	NA	9.457
TCGA-S9-A6UB-01A	16.237	15.865	11.303	11.096	11.303	10.419	NA	NA	NA
TCGA-S9-A6WD-01A	15.145	14.773	10.626	10.419	13.21	12.326	NA	NA	NA
TCGA-S9-A6WE-01A	14.715	14.343	NA	NA	13.365	12.481	NA	NA	NA

TCGA-S9-A6WG-01A	13.974	13.602	NA	NA	11.859	10.974	9.332	NA	NA
TCGA-S9-A6WH-01A	15.595	15.223	NA	NA	11.523	10.639	NA	NA	10.64
TCGA-S9-A6WI-01A	14.88	14.508	NA	NA	13.228	12.344	NA	NA	9.176
TCGA-S9-A6WL-01A	14.032	13.66	11.42	11.213	15.12	14.235	NA	NA	NA
TCGA-S9-A6WM-01A	15.62	15.248	NA	10.894	NA	NA	NA	NA	NA
TCGA-S9-A6WN-01A	12.972	12.6	10.553	10.346	9.5537	8.6708	NA	NA	NA
TCGA-S9-A6WO-01A	12.923	12.551	NA	NA	13.75	12.866	8.563	NA	NA
TCGA-S9-A6WP-01A	15.054	14.682	NA	NA	14.257	13.372	NA	NA	NA
TCGA-S9-A6WQ-01A	14.948	14.576	10.978	10.772	14.785	13.9	9.037	NA	NA
TCGA-S9-A7IQ-01A	16.023	15.651	11.323	11.116	12.907	12.023	NA	NA	NA
TCGA-S9-A7IS-01A	16.727	16.355	NA	NA	12.084	11.2	9.558	NA	NA
TCGA-S9-A7IX-01A	14.912	14.54	13.051	12.845	15.695	14.811	NA	NA	8.585
TCGA-S9-A7IY-01A	15.414	15.042	NA	NA	14.399	13.514	9.65	NA	NA
TCGA-S9-A7IZ-01A	15.572	15.201	12.712	12.505	14.034	13.149	NA	NA	NA
TCGA-S9-A7J0-01A	15.044	14.672	NA	NA	12.062	11.178	9.535	NA	NA
TCGA-S9-A7J1-01A	15.786	15.414	NA	NA	12.267	11.382	NA	NA	NA
TCGA-S9-A7J2-01A	15.928	15.556	NA	NA	13.882	12.998	NA	NA	9.677
TCGA-S9-A7J3-01A	16.089	15.717	14.061	13.855	NA	NA	NA	NA	NA
TCGA-S9-A7QW-01A	16.686	16.314	NA	NA	NA	NA	11.37	NA	NA
TCGA-S9-A7QX-01A	18.498	18.126	NA	NA	NA	NA	NA	NA	NA
TCGA-S9-A7QY-01A	16.575	16.203	11.105	10.898	11.105	10.221	11.16	NA	NA
TCGA-S9-A7QZ-01A	15.28	14.908	9.3836	9.1775	13.469	12.585	10.02	NA	11.08
TCGA-S9-A7R1-01A	16.059	15.687	11.692	11.485	14.013	13.129	NA	NA	NA
TCGA-S9-A7R2-01A	13.339	12.967	NA	NA	12.938	12.053	8.539	NA	NA
TCGA-S9-A7R3-01A	14.816	14.444	NA	NA	12.68	11.795	8.739	NA	NA
TCGA-S9-A7R4-01A	13.213	12.841	9.5147	9.3086	14.557	13.673	NA	NA	9.63
TCGA-S9-A7R7-01A	14.87	14.498	10.351	12.728	17.183	16.298	NA	NA	9.467
TCGA-S9-A7R8-01A	14.164	13.792	NA	NA	14.21	13.326	NA	NA	NA
TCGA-S9-A89V-01A	13.069	12.697	10.896	10.689	12.217	11.333	6.644	NA	NA
TCGA-S9-A89Z-01A	14.535	14.163	9.1303	8.9243	14.636	13.751	NA	NA	NA
TCGA-TM-A7C3-01A	14.03	13.658	NA	NA	9.1338	8.2515	9.19	NA	NA
TCGA-TM-A7C4-01A	14.716	14.344	NA	NA	13.058	12.174	10.7	NA	10.76
TCGA-TM-A7C5-01A	16.171	15.799	NA	NA	11.929	11.045	11.31	NA	NA
TCGA-TM-A7CA-01A	14.334	13.962	NA	NA	12.721	11.837	NA	NA	NA
TCGA-TM-A7CF-01A	14.261	13.889	10.649	10.443	13.456	12.571	NA	NA	NA
TCGA-TM-A84B-01A	15.569	15.197	NA	NA	NA	NA	NA	NA	NA
TCGA-TM-A84C-01A	15.067	14.695	NA	8.7915	11.995	11.111	NA	NA	NA
TCGA-TM-A84F-01A	15.371	14.999	9.4385	9.2323	11.437	10.553	NA	NA	NA
TCGA-TM-A84G-01A	15.132	14.76	NA	NA	NA	NA	9.186	NA	NA
TCGA-TM-A84H-01A	14.742	14.37	NA	NA	NA	NA	10.66	NA	9.722
TCGA-TM-A84I-01A	14.984	14.612	9.3504	9.1443	10.349	9.4657	NA	NA	NA
TCGA-TM-A84J-01A	15.169	14.797	NA	NA	12.235	11.35	9.418	NA	9.477
TCGA-TM-A84L-01A	14.882	14.51	NA	NA	12.441	11.557	9.178	NA	NA
TCGA-TM-A84M-01A	15.937	15.565	NA	NA	12.324	11.44	8.577	NA	NA
TCGA-TM-A84O-01A	13.505	13.133	NA	NA	12.67	11.785	NA	NA	NA
TCGA-TM-A84Q-01A	17.408	17.036	NA	NA	NA	NA	NA	NA	NA
TCGA-TM-A84R-01A	14.131	13.759	NA	NA	13.381	12.496	NA	NA	10.18
TCGA-TM-A84S-01A	15.542	15.17	NA	NA	12.081	11.197	NA	NA	NA
TCGA-TM-A84T-01A	14.065	13.693	NA	NA	13.172	12.287	NA	NA	NA
TCGA-TQ-A7RF-01A	14.409	14.037	NA	NA	NA	NA	NA	NA	NA
TCGA-TQ-A7RG-01A	15.678	15.306	NA	NA	12.191	11.307	9.664	NA	NA
TCGA-TQ-A7RH-01A	15.409	15.037	NA	NA	10.647	9.763	8.706	NA	8.765
TCGA-TQ-A7RI-01A	15.164	14.792	NA	NA	11.645	10.761	9.703	NA	NA

TCGA-TQ-A7RJ-01A	14.156	13.784	10.736	10.53	12.735	11.851	NA	NA	NA
TCGA-TQ-A7RK-01A	14.713	14.341	NA	NA	13.1	12.216	NA	NA	NA
TCGA-TQ-A7RM-01A	14.243	13.871	NA	NA	10.108	9.2244	10.16	NA	NA
TCGA-TQ-A7RN-01A	15.162	14.79	NA	NA	NA	NA	9.749	NA	NA
TCGA-TQ-A7RO-01A	15.806	15.434	NA	NA	11.439	10.555	11.5	NA	NA
TCGA-TQ-A7RP-01A	13.544	13.173	10.54	10.334	11.861	10.977	NA	NA	NA
TCGA-TQ-A7RQ-01A	15.418	15.046	NA	NA	11.718	10.833	NA	NA	NA
TCGA-TQ-A7RR-01A	15.095	14.723	NA	NA	10.529	9.645	9.586	NA	9.645
TCGA-TQ-A7RS-01A	16.906	16.534	NA	NA	10.936	10.052	10.99	NA	NA
TCGA-TQ-A7RV-01A	16.833	16.461	12.697	12.49	NA	NA	NA	NA	NA
TCGA-TQ-A7RW-01A	13.991	13.62	NA	NA	11.472	10.588	NA	NA	9.589
TCGA-TQ-A8XE-01A	15.724	15.352	NA	NA	NA	NA	NA	NA	NA
TCGA-VM-A8C8-01A	13.91	13.538	NA	NA	12.004	11.12	9.74	NA	7.804
TCGA-VM-A8C9-01A	12.406	12.035	NA	8.5905	10.964	10.08	7.856	NA	NA
TCGA-VM-A8CA-01A	13.603	13.231	10.183	9.9772	12.352	11.468	8.244	NA	NA
TCGA-VM-A8CB-01A	15.094	14.722	NA	NA	12.674	11.789	10.73	NA	NA
TCGA-VM-A8CD-01A	12.514	12.142	9.902	9.6957	11.486	10.602	NA	NA	NA
TCGA-VM-A8CE-01A	15.376	15.004	10.787	10.58	11.371	10.487	10.43	NA	NA
TCGA-VM-A8CF-01A	14.266	13.894	NA	NA	12.22	11.336	8.958	NA	NA
TCGA-VM-A8CH-01A	16.716	16.344	NA	NA	NA	NA	9.231	NA	NA
TCGA-VV-A829-01A	14.478	14.106	NA	NA	11.502	10.618	NA	NA	NA
TCGA-VV-A86M-01A	14.676	14.304	NA	10.103	13.893	13.008	9.367	NA	NA
TCGA-VW-A7QS-01A	16.631	16.259	NA	NA	11.869	10.985	NA	NA	NA
TCGA-VW-A8FI-01A	14.43	14.058	8.5172	8.3114	8.5172	7.636	NA	NA	NA
TCGA-W9-A837-01A	14.192	13.82	10.057	9.8503	10.057	9.1732	NA	NA	NA
TCGA-WH-A86K-01A	14.301	13.929	NA	NA	12.689	11.804	9.872	NA	8.349
TCGA-WY-A858-01A	14.117	13.745	8.7264	9.5185	13.581	12.697	NA	NA	NA
TCGA-WY-A859-01A	15.212	14.84	10.077	9.8704	13.397	12.513	NA	NA	NA
TCGA-WY-A85A-01A	15.49	15.118	NA	NA	13.499	12.614	NA	NA	9.295
TCGA-WY-A85B-01A	16.637	16.265	NA	10.461	NA	NA	NA	NA	NA
TCGA-WY-A85C-01A	15.017	14.645	NA	NA	11.945	11.061	NA	NA	NA
TCGA-WY-A85D-01A	14.825	14.453	NA	NA	10.599	9.7149	NA	NA	NA
TCGA-WY-A85E-01A	13.78	13.408	NA	NA	10.92	10.036	NA	NA	NA
TCGA-06-0675-11A	15.599	15.227	10.777	11.57	12.776	11.892	10.83	NA	11.89
TCGA-06-0678-11A	15.296	14.924	NA	NA	10.684	9.8	9.741	NA	11.38
TCGA-06-0680-11A	15.068	14.696	NA	NA	11.7	10.816	11.34	NA	9.817
TCGA-06-0681-11A	15.172	14.8	NA	NA	12.804	11.92	NA	9.982	9.921
TCGA-06-AABW-11A	16.034	15.662	NA	NA	12.778	11.893	9.836	NA	NA
